# Activin receptor-like kinase5 inhibition suppresses mouse melanoma by ubiquitin degradation of Smad4, thereby derepressing eomesodermin in cytotoxic T lymphocytes

**DOI:** 10.1002/emmm.201302524

**Published:** 2013-10-11

**Authors:** Jeong-Hwan Yoon, Su Myung Jung, Seok Hee Park, Mitsuyasu Kato, Tadashi Yamashita, In-Kyu Lee, Katsuko Sudo, Susumu Nakae, Jin Soo Han, Ok-Hee Kim, Byung-Chul Oh, Takayuki Sumida, Masahiko Kuroda, Ji-Hyeon Ju, Kyeong Cheon Jung, Seong Hoe Park, Dae-Kee Kim, Mizuko Mamura

**Affiliations:** 1 Department of Experimental Pathology, Graduate School of Comprehensive Human Science, University of TsukubaTsukuba, Japan; 2 Department of Internal Medicine, Research Institute of Aging and Metabolism, Kyungpook National University School of MedicineDaegu, Korea; 3 Department of Molecular Pathology, Tokyo Medical UniversityTokyo, Japan; 4 Department of Microbiology, CHA UniversitySeoul, Korea; 5 Department of Biological Sciences, Sungkyunkwan UniversitySuwon, Korea; 6 Laboratory of Veterinary Biochemistry, Azabu University School of Veterinary MedicineSagamihara, Japan; 7 Animal Research Center, Tokyo Medical UniversityTokyo, Japan; 8 Laboratory of Systems Biology, Center for Experimental Medicine and Systems Biology, The Institute of Medical Science, The University of TokyoTokyo, Japan; 9 Department of Laboratory Animal Medicine, Institute for the 3Rs, College of Veterinary Medicine, Konkuk UniversitySeoul, Korea; 10 Laboratory of Molecular and Cellular Biochemistry, Gachon UniversityIncheon, Korea; 11 Division of Clinical Immunology, Major of Advanced Biomedical Applications, Graduate School of Comprehensive Human Science, University of TsukubaTsukuba, Japan; 12 Department of Rheumatology, Catholic University of KoreaSeoul, Korea; 13 Department of Pathology, College of Medicine, Seoul National UniversitySeoul, Korea; 14 College of Pharmacy, Ewha Womans UniversitySeoul, Korea; 15 Department of Internal Medicine, Bundang CHA HospitalKyeonggi-do, Korea

**Keywords:** ALK5 inhibitor, Eomes, melanoma, Smad4, TGF-β

## Abstract

Varieties of transforming growth factor-β (TGF-β) antagonists have been developed to intervene with excessive TGF-β signalling activity in cancer. Activin receptor-like kinase5 (ALK5) inhibitors antagonize TGF-β signalling by blocking TGF-β receptor-activated Smad (R-Smad) phosphorylation. Here we report the novel mechanisms how ALK5 inhibitors exert a therapeutic effect on a mouse B16 melanoma model. Oral treatment with a novel ALK5 inhibitor, EW-7197 (2.5 mg/kg daily) or a representative ALK5 inhibitor, LY-2157299 (75 mg/kg bid) suppressed the progression of melanoma with enhanced cytotoxic T-lymphocyte (CTL) responses. Notably, ALK5 inhibitors not only blocked R-Smad phosphorylation, but also induced ubiquitin-mediated degradation of the common Smad, Smad4 mainly in CD8^+^ T cells in melanoma-bearing mice. Accordingly, T-cell-specific deletion of Smad4 was sufficient to suppress the progression of melanoma. We further identified eomesodermin (Eomes), the T-box transcription factor regulating CTL functions, as a specific target repressed by TGF-β via Smad4 and Smad3 in CD8^+^ T cells. Thus, ALK5 inhibition enhances anti-melanoma CTL responses through ubiquitin-mediated degradation of Smad4 in addition to the direct inhibitory effect on R-Smad phosphorylation.

## INTRODUCTION

TGF-β is the most potent immunosuppressive cytokine, which is abundantly produced and activated in the tumour microenvironment (Bierie and Moses, 2006; Flavell et al, 2010). TGF-β suppresses anti-tumour immunity by directly inhibiting the differentiation and functions of various effector cells, such as NK cells, Th1 cells and cytotoxic T lymphocytes (CTLs; Li et al, 2006). In addition to direct immune suppression, TGF-β indirectly suppresses anti-tumour immunity by inducing suppressor immune cell subsets, such as Foxp3^+^ regulatory T cells (Treg) and myeloid-derived suppressor cells (Flavell et al, 2010). To intervene with excessive TGF-β signalling activity to enhance anti-tumour immunity, varieties of TGF-β antagonists have been developed (Akhurst & Hata, 2012; Flavell et al, 2010; Hawinkels & ten Dijke, 2011). TGF-β type I receptor (TβRI) phosphorylates TGF-β receptor-activated Smads (R-Smads), Smad2 and Smad3, which form heteromeric complexes with the common Smad, Smad4, to translocate into the nuclei, where they regulate the target gene transcription (Massague et al, 2005). Activin receptor-like kinase5 (ALK5) inhibitors are the small molecule inhibitors, which block phosphorylation of R-Smads by occupying the ATP binding site of TβRI domain (Jin et al, 2011). On the basis of a selective, imidazole-based ALK5 inhibitor, 4-(4-(benzo[*d*][1,3]dioxol-5-yl)-5-(pyridin-2-yl)-1*H*-imidazol-2-yl)benzamide, SB-431542 (Callahan et al, 2002) as a lead compound, we designed and synthesized an orally bioavailable ALK5 inhibitor, *N*-((4-([1,2,4]triazolo[1,5-*a*]pyridin-6-yl)-5-(6-methylpyridin-2-yl)-1*H*-imidazol-2-yl)methyl)-2-fluoroaniline, EW-7197 (Kim et al, 2011).

Melanoma is a prototypical immunogenic tumour expressing melanoma-associated antigens, which are targeted by CTLs (Thomson et al, 1988). CTLs lyse the target tumour cells with the cytolytic molecules (Russell & Ley, 2002). The T-box transcription factors, T-bet and Eomes are crucial for the differentiation and effector functions of CTLs (Glimcher et al, 2004; Intlekofer et al, 2005; Pearce et al, 2003), which are required for anti-tumour immune responses (Zhu et al, 2010). Thus, intensive efforts have focused on developing immunotherapies to activate anti-melanoma T-cell responses (Kirkwood et al, 2008). However, melanoma cells produce high amounts of TGF-β, which limit the success of immunotherapy by rendering the host immune response tolerant to tumour-associated antigens (Javelaud et al, 2008).

In this study, we report the cellular and molecular mechanisms how EW-7197 and a representative ALK5 inhibitor, 4-(2-(6-methylpyridin-2-yl)-5,6-dihydro-4*H*-pyrrolo[1,2-b]pyrazol-3-yl)quinoline-6-carboxamide, LY-2157299 (Calvo-Aller et al, 2008) exert a therapeutic effect on a mouse model of B16 melanoma. ALK5 inhibition induced ubiquitin-mediated degradation of Smad4 in CD8^+^ T cells in addition to the direct inhibition of R-Smad phosphorylation to enhance anti-melanoma CTL responses through derepressing Eomes.

## RESULTS

### Selective inhibition of ALK5 suppresses the progression of melanoma with enhanced CTL activity

**Figure 1:**
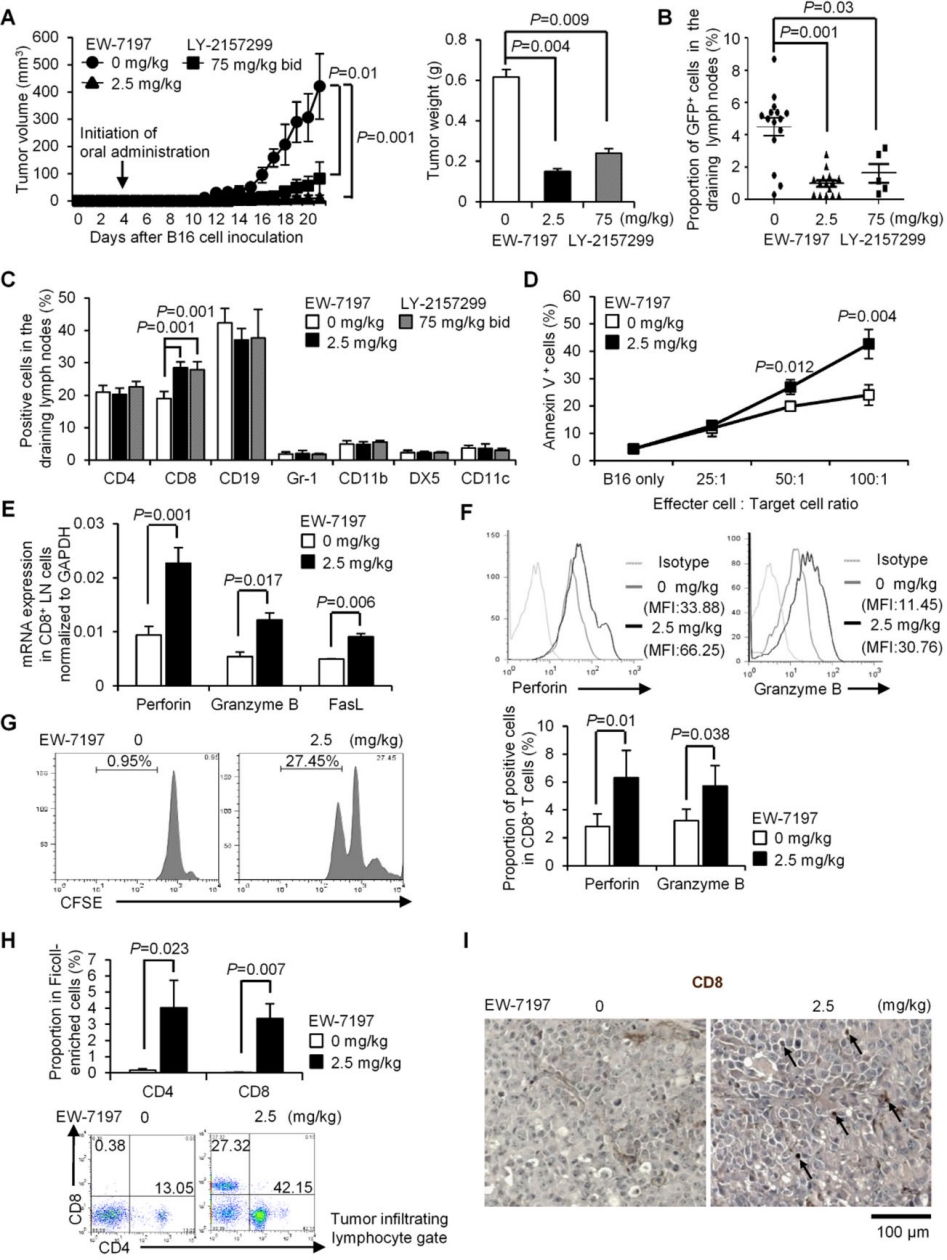
Oral administration of ALK5 inhibitors suppresses melanoma and LN metastases with enhanced CTL activity C57BL/6 mice were treated with vehicle or EW-7197 (2.5 mg/kg daily) (*n* = 15/group)/LY-2157299 (75 mg/kg bid) (*n* = 5) from 4 days after inoculation of GFP-expressing B16 cells (4 × 10^4^) into the left footpads. Data are shown as mean ± SEM. *P* values were calculated by 2-tailed unpaired Student's *t*-test or by two-way ANOVA test for (A). **A.** Chronological tumour volumes (left), tumour weights on Day 21 (right).**B,C.** The % of GFP^+^ B16 cells (medians ± interquartile) and immune cell subsets in dLNs were determined by flowcytometry.**D.** Target cytolysis at the indicated ratios of effector CD8^+^ T cells: target B16 cells was evaluated by annexin V/PI.**E.** qPCR analyses for mRNA levels of the cytolytic molecules in CD8^+^ dLN cells (*n* = 5/group).**F.** Histograms show CD8^+^ gate with MFI. Graphs show the % of positive cells in CD8^+^ gate (*n* = 10/group).**G.** Proliferation of CD8^+^ dLN cells stimulated with gp100 peptide was assessed by CFSE dilution.**H.** Representative CD4/8 dot plots of TILs. Graphs show the % of CD4^+^ or CD8^+^ cells in the Ficoll-enriched cells (*n* = 8/group).**I.** Representative immunohistochemistry sections of inoculated melanomas (scale bar: 100 μm). Arrows indicate CD8^+^ cells.

To examine the therapeutic efficacy of EW-7197 for melanoma in comparison with LY-2157299 for eventual use in a Phase 2 clinical trial (Akhurst & Hata, 2012; Calvo-Aller et al, 2008; Hawinkels & ten Dijke, 2011), C57BL/6 mice were orally administered with vehicle or vehicle containing EW-7197 (2.5 mg/kg daily) or LY-2157299 (75 mg/kg bid) starting from 4 days after inoculation of GFP-expressing B16 cells (4 × 10^4^) into the left footpads. Low-dose EW-7197 was more efficient than high-dose LY-2157299 in suppressing the growth of transplanted tumours (Fig 1A). Treatment with EW-7197 and LY-2157299 efficiently suppressed the lymph node (LN) metastases, which were detected by CD11c^−^CD11b^−^B220^−^GFP^+^ cells in the draining lymph nodes (dLNs; Fig 1B and Supporting Information Fig S1).

Because TGF-β and EW-7197 showed no direct effects on apoptosis and cell cycle of B16 cells *in vitro* (Supporting Information Fig S2) and TGF-β antagonism mainly targets the immune system rather than the cancer cells (Donkor et al, 2011; Nam et al, 2008), we evaluated the effect of EW-7197 on immunophenotypes of melanoma-bearing mice. Treatment with EW-7197 increased the proportions and numbers of CD8^+^ T cells significantly in the dLNs (Fig 1C and Supporting Information Fig S3A), non-dLNs and spleens (Supporting Information Fig S3B). Other effector T-cell subsets were unaltered (Supporting Information Fig S3C). Splenic CD8^+^ T cells as effector cells were prepared from vehicle- or EW-7197-treated mice for co-culture with target B16 cells to examine CTL function. CD8^+^ T cells from EW-7197-treated mice induced significantly more apoptosis of target B16 cells (Fig 1D). The mRNA expression of the cytolytic molecules, perforin, granzyme B and FasL in whole dLNs and CD8^+^ dLN cells and protein expression of perforin and granzyme B in dLN CD8^+^ T cells of EW-7197-treated mice increased significantly (Fig 1E, F and Supporting Information Fig S3D and E).

To confirm whether enhanced CD8^+^ T-cell responses by EW-7197 are antigen-specific, we stimulated the carboxyfluorescein diacetate succinmidyl ester (CFSE)-labelled dLN cells with gp100 peptide, a melanosomal differentiation Ag expressed by melanomas and melanocytes (Thomson et al, 1988) and determined CFSE dilution of CD8^+^ gate by flowcytometry. CD8^+^ cells from EW-7197-treated mice showed significantly enhanced proliferation compared with CD8^+^ cells from vehicle-treated mice (Fig 1G). Tumour-infiltrating lymphocytes (TILs) increased significantly in the melanomas of EW-7197-treated mice, which were rarely observed in those of vehicle-treated mice (Fig 1H and Supporting Information Fig S3F). Especially, CD8^+^ cell infiltration was remarkable in the melanomas of EW-7197-treated mice, which was absent in those of vehicle-treated mice (Fig 1H and I). These data show that oral administration of a novel ALK5 inhibitor, EW-7197 has a potent therapeutic effect on B16 melanoma by upregulating CTL activities.

### ALK5 inhibition downregulates Smad4 in melanoma-bearing mice

**Figure 2:**
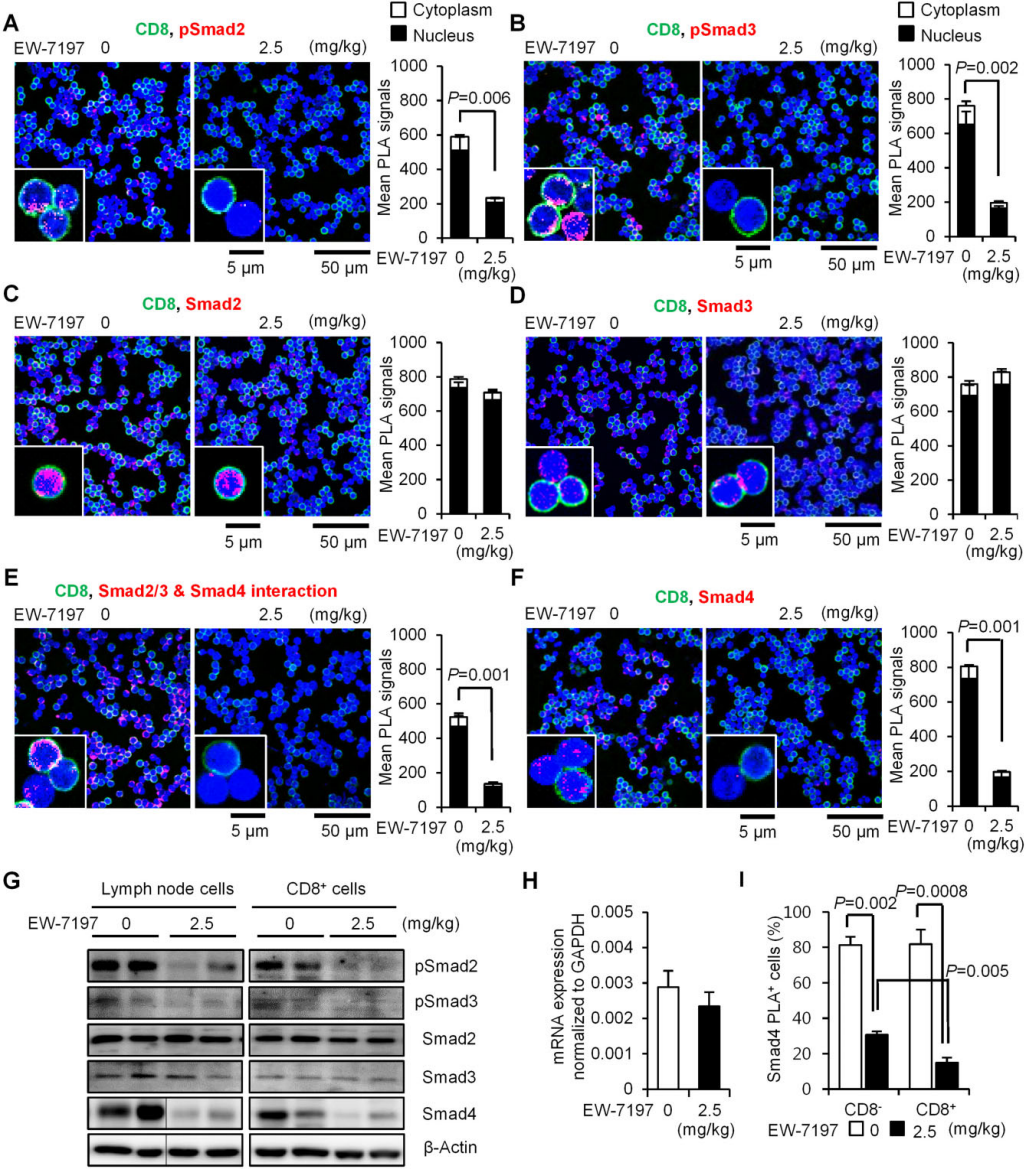
EW-7197 downregulates Smad4 and blocks R-Smad phosphorylation in melanoma-bearing mice Source data is available for this figure in the Supporting Information. Data are shown as mean + SEM (*n* = 5/group). *P* values were calculated by 2-tailed unpaired Student's *t*-test. **A–F.** PLA (red) show the expression of phosho-Smad2, phosho-Smad3, Smad2, Smad3, Smad4 and the close proximity between Smad2/3 and Smad4 in dLN cells co-stained with anti-CD8 (green; scale bars: 5 µm, 50 µm). Graphs show mean PLA signals in nuclei (black) and cytoplasms (white) quantified using BlobFinder software.**G.** Western blots show Smads in whole or CD8^+^ dLN cells from EW-7197-treated or vehicle-treated melanoma-bearing mice (two to three mice/group).**H.** qPCR analyses for Smad4 mRNA levels of dLN cells.**I.** Graph shows the % of the Smad4 PLA^+^ cells in CD8^−^ and CD8^+^ dLN cells.

We next confirmed the blockade of TGF-β signalling by EW-7197 *in vivo*. Cells of dLNs and spleens from melanoma-bearing mice were immediately fixed for proximity ligation assay (PLA) to quantify endogenous Smad protein levels by single recognitions or close proximity of two proteins within 40 nm by double recognitions. EW-7197 blocked phosphorylation of Smad2 and Smad3 in dLN cells, while the expression levels of Smad2 and Smad3 were intact (Fig 2A–D). Although phosphorylation of R-Smads is often monitored to confirm the efficacy of TGF-β antagonists (Donkor et al, 2011), their effect on Smad4 has not been evaluated. Treatment with EW-7197 abolished close proximity between Smad2/3 and Smad4 (Fig 2E). Moreover, we found that EW-7197 significantly reduced Smad4 protein in both nucleus and cytoplasm of dLN cells (Fig 2F). The same pattern was confirmed in spleens of EW-7197-treated mice and dLNs of LY-2157299-treated mice (Supporting Information Fig S4 and S5A–D). Western blot analysis confirmed the reduction in Smad4 protein and R-Smad phosphorylation with intact R-Smad expression in dLNs and CD8^+^ dLN cells by ALK5 inhibitors (Fig 2G and Supporting Information Fig S5E). However, EW-7197 did not affect Smad4 mRNA (Fig 2H), indicating that EW-7197 did not downregulate Smad4 at the transcriptional level. Reduction in Smad4 protein was most remarkable in CD8^+^ T cells (Fig 2G and I).

### ALK5 inhibition induces ubiquitin-mediated degradation of Smad4 in melanoma-bearing mice

**Figure 3:**
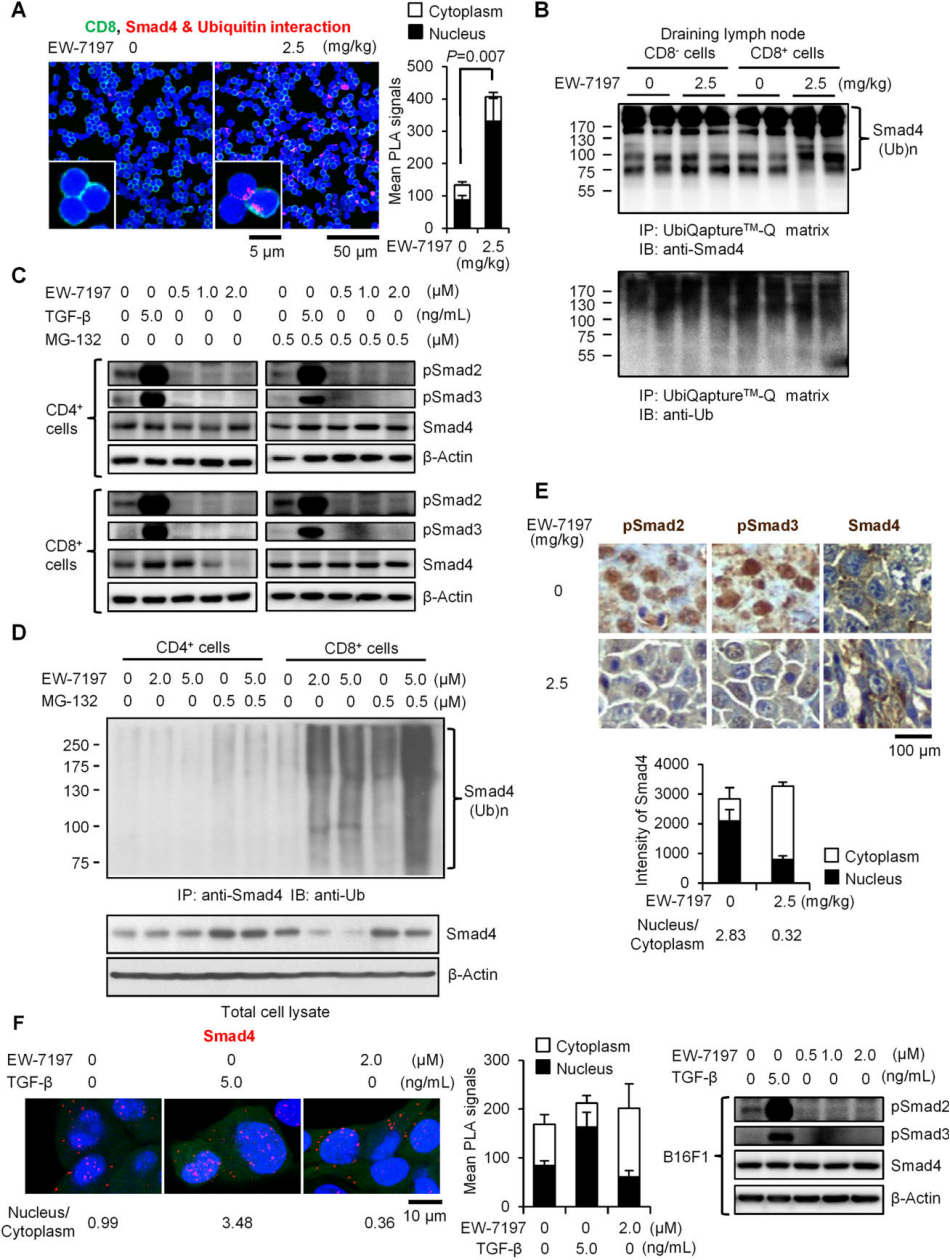
ALK5 inhibition induces ubiquitin-mediated degradation of Smad4 in CD8^+^ T cells in melanoma-bearing mice Source data is available for this figure in the Supporting Information. PLA (red) show the close proximity between ubiquitin and Smad4 in the dLN cells co-stained with anti-CD8 (green) (scale bars: 5 µm, 50 µm). Graphs show mean PLA signals in nuclei (black) and cytoplasms (white) quantified using BlobFinder software.Upper panel shows endogenous ubiquitinated Smad4 and lower panel shows ubiquitinated proteins in CD8^+^ and CD8^−^ dLN cells. Ubiquitinated proteins were captured using an UbiQapture™-Q kit and blotted with anti-Smad4 or anti-ubiquitin antibody. Molecular weight of Smad4 is 70 kD.Western blots show Smads in CD4^+^ and CD8^+^ cells stimulated with anti-CD3/CD28 with/without EW-7197 and/or MG-132 for 3 days.IP-Western blot shows endogenous ubiquitinated Smad4 in CD4^+^ and CD8^+^ cells stimulated with anti-CD3/CD28 with/without EW-7197 and/or MG-132 for 3 days.Representative immunohistochemistry sections of inoculated melanomas (scale bar: 100 µm). Graph shows the subcellular distributions of Smad4 expression in melanoma cells calculated by ImageJ software. The expression ratios of nucleus to cytoplasm are shown.Smad4 protein in B16 cells was detected by PLA (red; scale bars: 10 µm; left). The expression ratios of nucleus to cytoplasm are shown. Graph shows the subcellular distributions of Smad4 in B16 cells. Western blots show Smads in B16 cells cultured with EW-7197 with or without TGF-β1 (right).

Ubiquitination is a post-translational modification of proteins, which plays a key role in TGF-β signal transduction by regulating Smad protein levels (De Boeck & ten Dijke, 2012; Izzi & Attisano, 2004). PLA detected the significantly increased close proximity between ubiquitin and Smad4 in dLN cells, especially in CD8^+^ T cells of the melanoma-bearing mice treated with EW-7197 or LY-2157299 (Fig 3A, Supporting Information Fig S6). By contrast, neither Smad2 nor Smad3 showed close proximity with ubiquitin in dLN cells of both vehicle- and EW-7197-treated mice (Supporting Information Fig S7). To confirm whether Smad4 in close proximity with ubiquitin by the treatment with EW-7197 is ubiquitinated, endogenous ubiquitinated Smad4 was captured by UbiQapture matrices. Ubiquitination of Smad4 was enhanced significantly in CD8^+^ dLN cells by the treatment with EW-7197, whereas it was not altered in CD8^−^ dLN cells (Fig 3B). Consistently, EW-7197 also induced downregulation of Smad4 protein in CD8^+^ T cells stimulated with anti-CD3/CD28 antibodies *in vitro*, but not in CD4^+^ T cells, although it inhibited R-Smad phosphorylation in both CD4^+^ and CD8^+^ T cells (Fig 3C). A proteasome inhibitor, MG-132 abolished EW-7197-induced downregulation of Smad4 in CD8^+^ T cells (Fig 3C), indicating that the ubiquitin-proteasomal system is responsible for Smad4 protein degradation. EW-7197 induced ubiquitination of Smad4 accompanied with protein downregulation in activated CD8^+^ T cells, but not CD4^+^ T cells in a dose dependent manner (Fig 3D). Unlike CD8^+^ T cells, treatment with EW-7197 did not affect the expression levels of total Smad4 protein in both transplanted B16 melanomas *in vivo* and B16 melanoma cells *in vitro* (Fig 3E and F). Oral treatment with EW-7197 suppressed R-Smad phosphorylation in B16 melanomas (Fig 3E). Consistently, EW-7197 exerted the reverse effect of TGF-β on Smad4 subcellular localization: increases in the cytoplasms and decreases in the nuclei of B16 melanoma cells both *in vivo* and *in vitro* (Fig 3E and F).

Among the E3 ubiquitin ligases, which modulate TGF-β signalling, Smurf2 is upregulated by IL-7 in CD8^+^ T cells (Pellegrini et al, 2009). However, knockdown of Smurf1 and/or Smurf2 by shRNA did not affect Smad4 downregulation by EW-7197 in CD8^+^ T cells (Supporting Information Fig S8).

Taken together, systemic ALK5 inhibition in melanoma-bearing mice blocks TGF-β signalling by not only inhibiting R-Smad phosphorylation, but also inducing ubiquitin-mediated degradation of Smad4 protein in immune cells, especially in CD8^+^ T cells, whereas ALK5 inhibition suppresses intact Smad4-mediated TGF-β signalling in B16 melanoma cells.

### T-cell-specific Smad4 deletion suppresses the progression of melanoma with enhanced CTL activity

**Figure 4:**
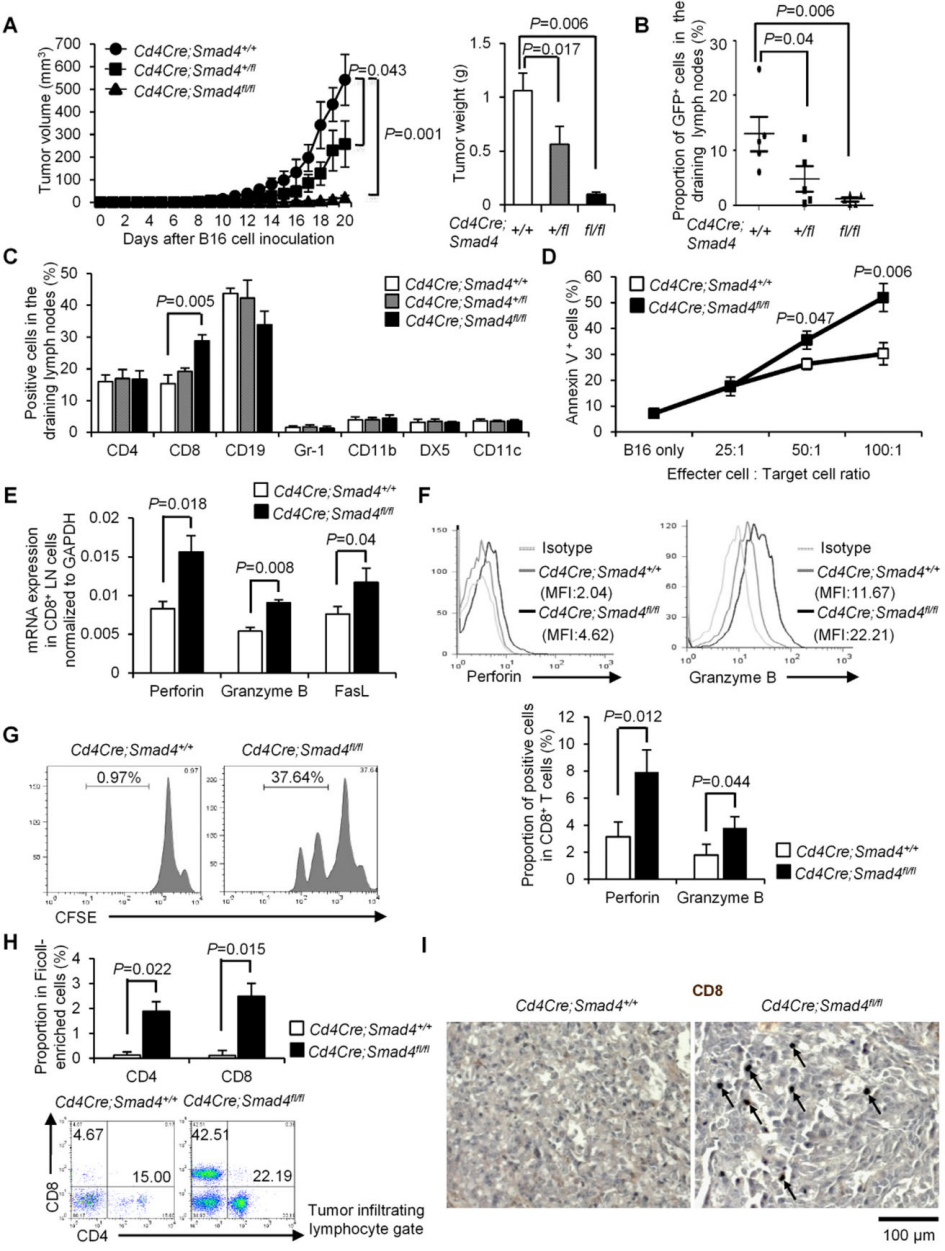
T-cell-specific Smad4 deletion suppresses melanoma and LN metastases with enhanced CTL activity GFP-expressing B16 cells were inoculated into the left footpads of *Cd4Cre;Smad4*^*+/+*^, *Cd4Cre;Smad4*^*+/fl*^ and *Cd4Cre;Smad4*^*fl/fl*^ mice. Data are shown as mean ± SEM (*n* = 5–8/genotype). *P* values were calculated by 2-tailed unpaired Student's *t*-test or by two-way ANOVA test for (A). **A.** Chronological tumour volumes (left), tumour weights on Day 21 (right).**B,C.** The % of GFP^+^ B16 cells (medians ± interquartile) and immune cell subsets in dLNs were determined by flowcytometry. (*n* = 25/genotype).**D.** Cytolysis at the indicated ratios of effector CD8^+^ T cells: target B16 cells.**E.** qPCR analyses for mRNA levels of the cytolytic molecules in CD8^+^ dLN cells (*n* = 5/genotype).**F.** Histograms show CD8^+^ gate with MFI. Graphs show the % of positive cells in CD8^+^ gate (*n* = 25/genotype).**G.** Proliferation of CD8^+^ dLN cells stimulated with gp100 peptide was assessed by CFSE dilution.**H.** Representative CD4/8 dot plots of TILs. Graphs show the % of CD4^+^ or CD8^+^ cells in the Ficoll-enriched cells (*n* = 5/genotype).**I.** Representative immunohistochemistry sections of inoculated melanomas (scale bar: 100 μm). Arrows indicate CD8^+^ cells.

Similarly with Smad4 downregulation by EW-7197 treatment, the orthotopic B16 melanoma model using T-cell-specific Smad4 knockout mice (Kim et al, 2006) showed significant suppression of melanoma growth and LN metastases (Fig 4A and B). CD8^+^ T cells increased significantly in the dLNs (Fig 4C), non-dLNs and spleens (Supporting Information Fig S9A) of *Cd4Cre;Smad4*^*fl/fl*^ (*Smad4*^*−/−*^) mice. Other effector T-cell subsets were unaltered by the *Smad4* genotypes (Supporting Information Fig S9B). The cytotoxicity assay showed significantly more B16 lysis by *Smad4*^*−/−*^ CD8^+^ T cells (Fig 4D). The mRNA and protein expression of cytolytic molecules increased significantly in whole dLNs and CD8^+^ dLN cells of *Smad4*^*−/−*^ mice, as in EW-7197-treated mice (Fig 4E, F and Supporting Information Fig S9C).

Stimulation with gp100 peptide induced significantly more proliferation of CD8^+^ dLN cells from *Smad4*^*−/−*^ mice compared with *Cd4Cre;Smad4*^*+/+*^ (*Smad4*^*+/+*^) mice (Fig 4G). TILs increased significantly in the melanomas of *Smad4*^*−/−*^ mice, which were rarely observed in those of *Smad4*^*+/+*^ mice (Fig 4H and Supporting Information Fig S9D). Especially, CD8^+^ cell infiltration was remarkable in the melanomas of *Smad4*^*−/−*^ mice, which was absent in those of *Smad4*^*+/+*^ mice (Fig 4H and I). These data are essentially identical to those obtained from EW-7197-treated mice, suggesting that TGF-β suppresses antigen-specific CTL functions via Smad4 without affecting other effector T-cell subsets, and that treatment with EW-7197 phenocopies the effect of T-cell specific Smad4 knockout.

### ALK5 inhibition and T-cell-specific Smad4 deletion upregulate Eomes in CD8^+^ T cells of melanoma-bearing mice

**Figure 5:**
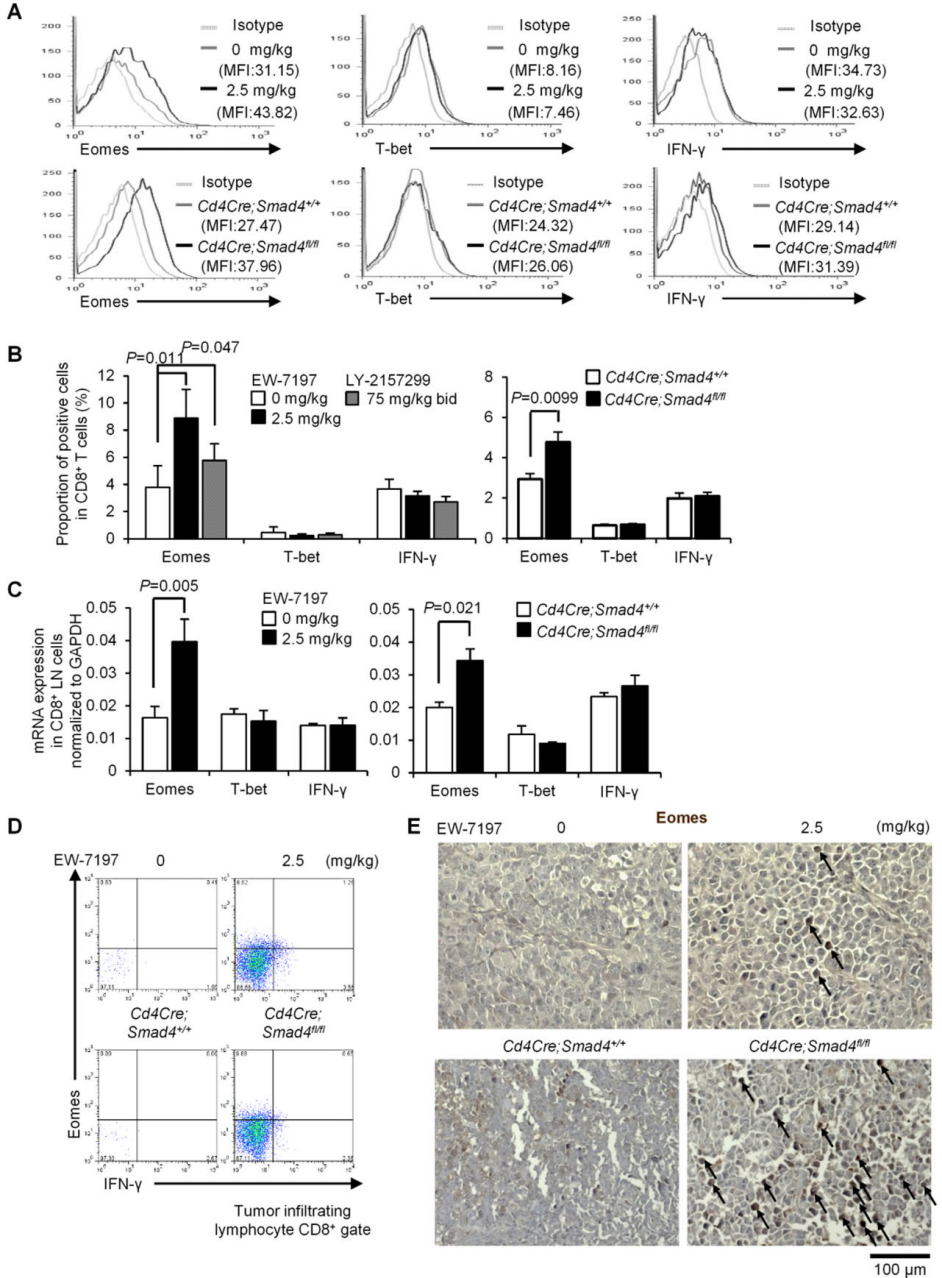
Upregulation of Eomes in CD8^+^ T cells of melanoma-bearing mice by the treatment with ALK5 inhibitors and T-cell-specific Smad4 deletion Data are shown as mean + SEM. *P* values were calculated by 2-tailed unpaired Student's *t*-test. Representative histograms show Eomes, T-bet and IFN-γ expression in CD8^+^ dLN cells with MFI.Graphs show the % of positive cells in CD8^+^ dLN cells of EW-7197 (*n* = 10) or LY-2157299 (*n* = 5) treated or *Cd4Cre;Smad4*^*+/+*^ and *Cd4Cre;Smad4*^*fl/fl*^ (*n* = 10/genotype) melanoma-bearing mice.qPCR analyses for mRNA levels in CD8^+^ dLN cells (*n* = 5/group, *n* = 5/genotype).Representative Eomes/IFN-γ dot plots of CD8^+^ gated TILs. Graphs show the % of positive cells in the Ficoll-enriched cells (*n* = 8/group, *n* = 5/genotype).Representative immunohisochemistry sections of inoculated melanomas (scale bar: 100 μm). Arrows indicate Eomes^+^ cells.

To address underlying mechanisms of enhanced CTL activity by Smad4 downregulation, we examined the master transcription factors for CTLs, T-bet and Eomes. T-bet suppresses metastases (Peng et al, 2004), and TGF-β1 suppresses T-bet and IFN-γ in CD4^+^ T cells (Park et al, 2007). However, expression of neither T-bet nor IFN-γ in CD8^+^ T cells was affected (Fig 5A and B). Instead, we found that Eomes^+^ CD8^+^ T cells increased significantly in dLNs of melanoma-bearing mice by the treatment with EW-7197, LY-2157299 or T-cell-specific Smad4 deletion (Fig 5A, B and Supporting Information Fig S10A–C). Eomes mRNA expression in dLNs and CD8^+^ dLN cells also increased significantly by EW-7197 or T-cell-specific Smad4 deletion (Fig 5C and Supporting Information Fig S10D). Expression of Eomes in CD4^+^ T cells was very low in any melanoma-bearing mice (Supporting Information Fig S11). CD8^+^ TILs in EW-7197-treated or *Smad4*^*−/−*^ mice expressed high levels of Eomes (Fig 5D). Significantly more Eomes^+^ cells infiltrated into the melanomas by EW-7197 or T-cell-specific Smad4 deletion (Fig 5E). Proportions of TIL subsets except T cells in EW-7197-treated or *Smad4*^*−/−*^ mice were unaltered compared with the controls (Supporting Information Fig S12). These data suggest that Smad4-mediated TGF-β signalling suppresses CTLs by specific downregulation of Eomes.

### Anti-melanoma effect of EW-7197 depends on CD8^+^ T cells

**Figure 6:**
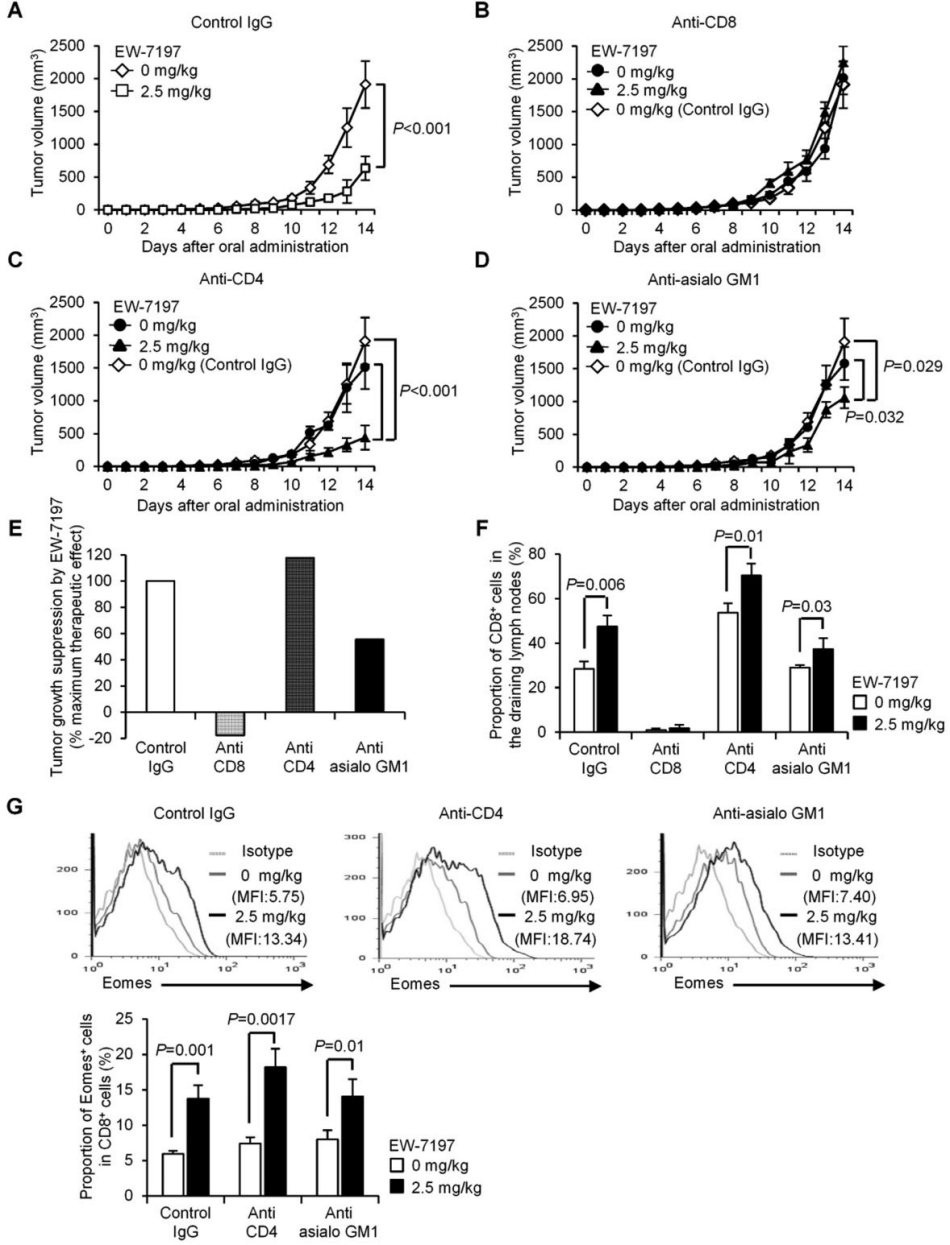
CD8^+^ T cells are necessary for anti-melanoma effect of EW-7197 C57BL/6 mice were i.p. injected with control, anti-CD8, anti-CD4 or anti-asialo GM1 antibody at Day −4, 0, 7 and 14 of melanoma inoculation (Day 0), with vehicle or EW-7197 from 4 days after inoculation of GFP-expressing B16 cells (2 × 10^5^) into the left lower abdomen (*n* = 5–8/group). Data are shown as mean ± SEM. *P* values were calculated by 2-tailed unpaired Student's *t*-test or by two-way ANOVA test. **A–D.** Chronological tumour volumes of the mice treated with the indicated antibodies.**E.** The efficacy of EW-7197 following each antibody treatment was expressed as a % of the maximum therapeutic effect seen in the intact system (control IgG).**F.** The % of CD8^+^ cells in dLNs was determined by flowcytometry.**G.** Histograms show the expression of Eomes in CD8^+^ dLN cells. The graph shows the % of Eomes^+^ in CD8^+^ dLN cells were determined by flowcytometry.

To confirm whether CD8^+^ T cells are necessary for anti-melanoma effect of EW-7197, we deleted CD8^+^, CD4^+^ or NK cells in C57BL/6 mice inoculated with GFP-expressing B16 cells (2 × 10^5^). Intraperitoneal injection of anti-CD8, anti-CD4 or anti-asialo GM1 antibody efficiently deleted the specific cell compartment, respectively (Fig 6B–D and Supporting Information Fig S13A). EW-7197 significantly suppressed tumour growth even with this aggressive protocol (Fig 6A). Deletion of CD8^+^, CD4^+^ or NK cells did not affect tumour growth in the absence of EW-7197 treatment (Fig 6A–D). Anti-tumour effect of EW-7197 was completely abolished on deletion of CD8^+^ cells, rather, EW-7197 slightly exacerbated tumour growth in CD8^+^-deleted mice (Fig 6B and E). In contrast, EW-7197 showed significant anti-tumour efficacy on deletion of CD4^+^ cells or NK cells (Fig 6C–E). In NK-deleted mice, we observed approximately 40% reduction in the efficacy of EW-7197 on tumour growth and CD8^+^ T-cell expansion (Fig 6D–F), suggesting that EW-7197 exerts the efficacy partially via NK cells, similarly to the previous report on the efficacy of the neutralizing anti-TGF-β antibody 1D11 on a mouse 4T1 model of metastatic breast cancer (Nam et al, 2008). Treatment with EW-7197 resulted in a significant increase in CD8^+^ T cells with upregulated Eomes expression in CD4^+^-deleted and NK-deleted mice as well as control (Fig 6F, G and Supporting Information Fig S13B). These data verify the previous reports that anti-tumour effect of the TGF-β antagonism mainly depends on CD8^+^ T cells (Donkor et al, 2011; Gorelik & Flavell, 2001; Nam et al, 2008; Zhang et al, 2005).

### Long-term systemic administration of EW-7197 and T-cell-specific Smad4 deletion maintain normal immune homeostasis

**Figure 7:**
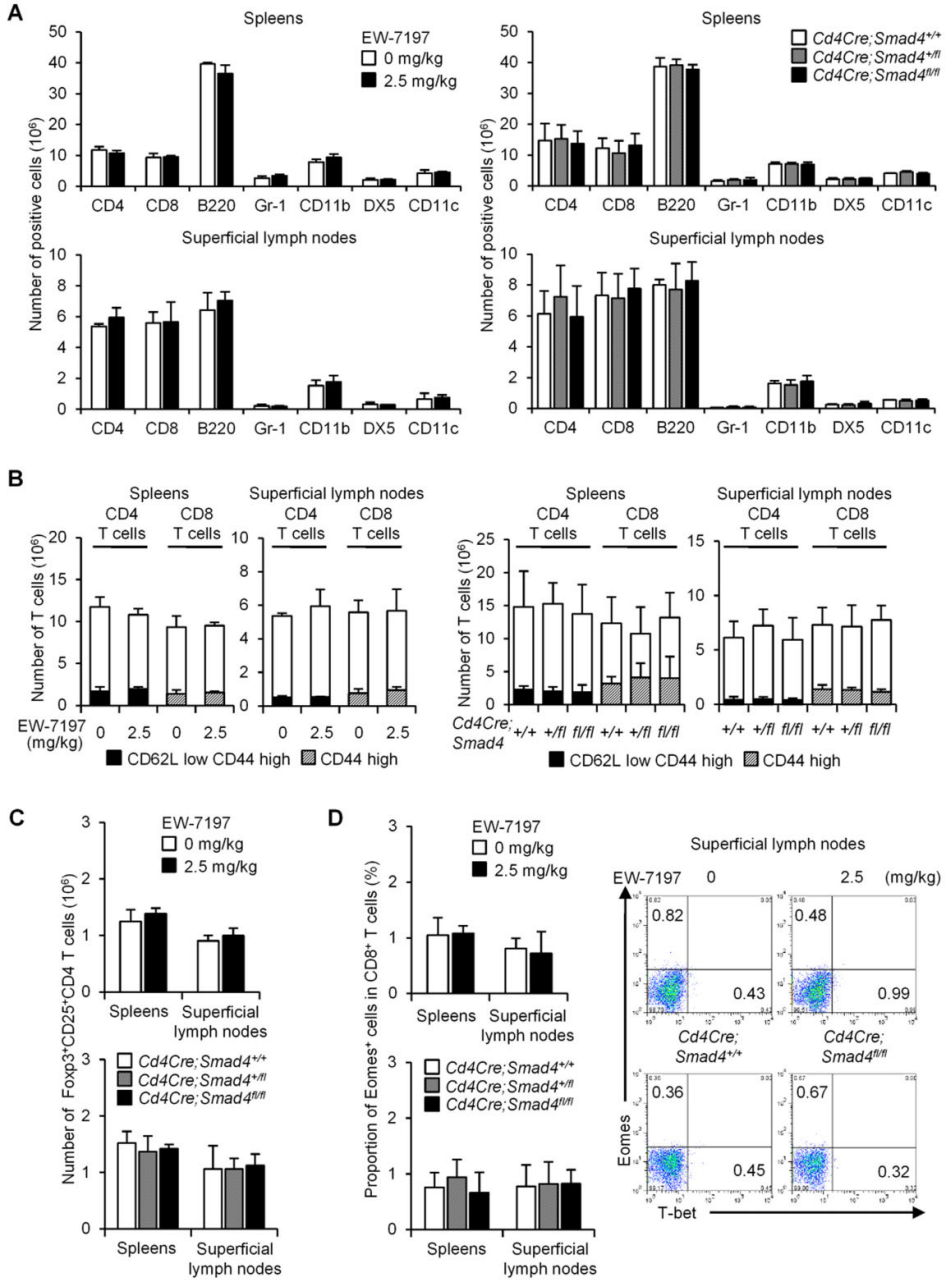
Normal immune homeostasis by long-term systemic administration of EW-7197 or T-cell-specific Smad4 deletion Immune cell populations in the spleens and superficial LNs of C57BL/6 mice treated with vehicle or vehicle containing EW-7197 (2.5 mg/kg daily) for 8 weeks (*n* = 5/group), *Cd4Cre;Smad4*^+/+^, *Cd4Cre;Smad4*^*+/fl*^ and *Cd4Cre;Smad4*^*fl/fl*^ mice (*n* = 15/genotype) at 16 weeks of age were analysed by flowcytometry. Graphs show mean + SEM. No statistical significance was observed by 2-tailed unpaired Student's *t*-test. **A–C.** Graphs show the cell numbers of immune cell subsets, naïve/memory CD4^+^/CD8^+^ T cells, and Foxp3^+^CD25^+^CD4^+^ cells in the spleens and superficial LNs determined by flowcytometry.**D.** Graph shows the % of Eomes^+^ in CD8^+^ gate. Representative dot plots show the expression of Eomes and T-bet in CD8^+^ gate.

We determined whether Smad4 downregulation by ALK5 inhibition or gene deletion causes pro-inflammatory untoward effects because T-cell-specific Smad4 deficient mice with mixed backgrounds (C57BL/6, Sv129 and FVB) develop inflammation and carcinogenesis in gastrointestinal tract (Hahn et al, 2011; Kim et al, 2006). *Cd4Cre;Smad4*^*+/fl*^ mice were backcrossed to C57BL/6 strain for eight generations and confirmed the deletion of the *Smad4* gene in both CD4^+^ and CD8^+^ T cells (Supporting Information Fig S14). C57BL/6 mice were treated with vehicle or vehicle containing EW-7197 (2.5 mg/kg daily) for 8 weeks. The proportions and numbers of immune cells, naïve/memory CD4^+^/CD8^+^ T cells, and Treg in the spleens and superficial LNs of vehicle-treated or Smad4^+/+^ mice were comparable to those of EW-7197-treated mice or *Smad4*^*−/−*^ mice at 16 week-old (Fig 7A–C). Low expression levels of Eomes and T-bet in steady-state CD8^+^ T cells were not altered by EW-7197 or T-cell-specific Smad4 deletion (Fig 7D). Consistent with normal immune homeostasis by lifetime exposure to a soluble TGF-β antagonist (Yang et al, 2002), treatment with EW-7197 for 8 weeks maintained normal immune homeostasis (Fig 7A–D).

Thus, long-term systemic administration of EW-7197 or T-cell-specific Smad4 deletion did not affect systemic immune homeostasis in C57BL/6 mice without melanoma challenge in a specific pathogen-free (SPF) environment.

### TGF-β suppresses Eomes via Smad4 and Smad3 in CD8^+^ T cells

**Figure 8:**
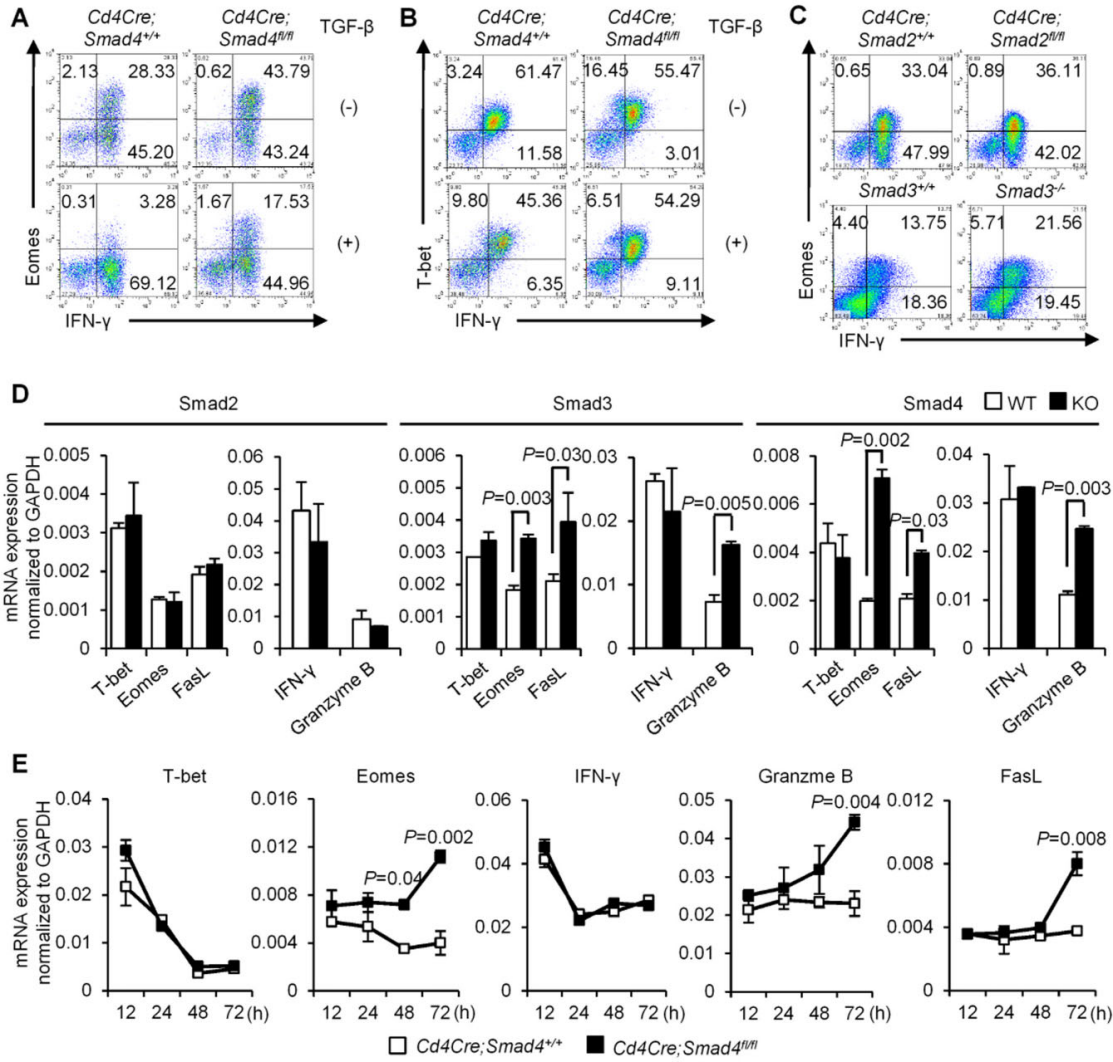
TGF-β signalling through Smad4 and Smad3 suppresses Eomes and the cytolytic molecules in CD8^+^ T cells CD8^+^ cells from the indicated mice were stimulated with anti-CD3/CD28 with or without TGF-β1 for 3 days. Data are shown as mean ± or + SEM. *P* values were calculated by 2-tailed unpaired Student's *t*-test. **A–C.** Representative dot plots show Eomes/IFN-γ, T-bet/IFN-γ in CD8^+^ cells from *Cd4Cre;Smad4*^*+/+*^/*Cd4Cre;Smad4*^*fl/fl*^ mice and Eomes/IFN-γ in CD8^+^ cells from *Cd4Cre;Smad2*^*+/+*^/*Cd4Cre;Smad2*^*fl/fl*^ and *Smad3*^*+/+*^/*Smad3*^*−/−*^ mice (*n* = 5–7/genotype).**D,E.** qPCR analyses (*n* = 5/genotype) for T-bet, Eomes, IFN-γ, granzyme B and FasL mRNA levels in CD8^+^ cells from *Cd4Cre;Smad4*^*fl/fl*^/*Cd4Cre;Smad2*^*fl/fl*^/*Smad3*^*−/−*^/control mice at 72 h and *Cd4Cre;Smad4*^*+/+*^/*Cd4Cre;Smad4*^*fl/fl*^ mice at the indicated time points.

We examined the effect of Smad4 deficiency on the expression of IFN-γ, T-bet and Eomes in CD8^+^ T cells stimulated with anti-CD3 and anti-CD28 antibodies *in vitro*. Consistent with the *in vivo* data, *Smad4*^−/−^ CD8^+^ T cells expressed significantly higher levels of Eomes than those in *Smad4*^+/+^ CD8^+^ T cells (Fig 8A). TGF-β1 (5 ng/ml) completely suppressed Eomes in *Smad4*^+/+^ CD8^+^ T cells, whereas the suppressive effect of TGF-β1 on Eomes was partially impaired in *Smad4*^−/−^ CD8^+^ T cells (Fig 8A). However, TGF-β1 and Smad4 deficiency had only a slight effect on T-bet in CD8^+^ T cells (Fig 8B). Stimulation with phorbol-12-myristate-13-acetate (PMA) and ionomycin showed the same trend (Supporting Information Fig S15).

We activated CD8^+^ T cells from *Cd4Cre;Smad2*^*+/+*^/*Cd4Cre;Smad2*^*fl/fl*^ or *Smad3*^+/+^/*Smad3*^*−/−*^ mice *in vitro* to examine which R-Smad was responsible for Smad4-mediated suppression of Eomes. Eomes^+^ cells increased significantly in the absence of Smad3, but not Smad2 (Fig 8C). Deficiency of Smad3 showed the intermediate effect between deficiency of Smad2 and Smad4 on the increase of Eomes^+^ cells.

We examined the effects of Smad deficiency on mRNA expression of granzyme B and FasL in CD8^+^ T cells because Eomes upregulates these cytolytic molecules (Pearce et al, 2003). Consistent with the *in vivo* expression patterns, Eomes, granzyme B and FasL mRNA levels in *Smad4*^−/−^ CD8^+^ T cells were significantly higher than those in *Smad4*^+/+^ CD8^+^ T cells, whereas T-bet and IFN-γ mRNA levels in *Smad4*^−/−^ CD8^+^ T cells were similar to those in *Smad4*^+/+^ CD8^+^ T cells (Fig 8D). Eomes, granzyme B and FasL mRNA levels were unaltered in *Smad2*^−/−^ CD8^+^ T cells, whereas those in *Smad3*^−/−^ CD8^+^ T cells were intermediate between *Smad4*^−/−^ and *Smad2*^−/−^ CD8^+^ T cells (Fig 8D).

Effector CTL differentiation occurs in two sequential phases, early induction of T-bet and late induction of Eomes (Cruz-Guilloty et al, 2009). Smad4 deficiency did not affect T-bet mRNA, which peaked at 12 h (Fig 8E). By contrast, late induction of Eomes (48, 72 h), granzyme B and FasL mRNA (72 h) was further upregulated in *Smad4*^−/−^ CD8^+^ T cells (Fig 8E). Thus, TGF-β signalling through Smad4 does not affect T-bet even at the early phase. Taken together, TGF-β signalling through Smad4 and Smad3, but not Smad2, suppresses Eomes and the cytolytic molecules.

### Smad4 represses the *Eomes* gene in CD8^+^ T cells

**Figure 9:**
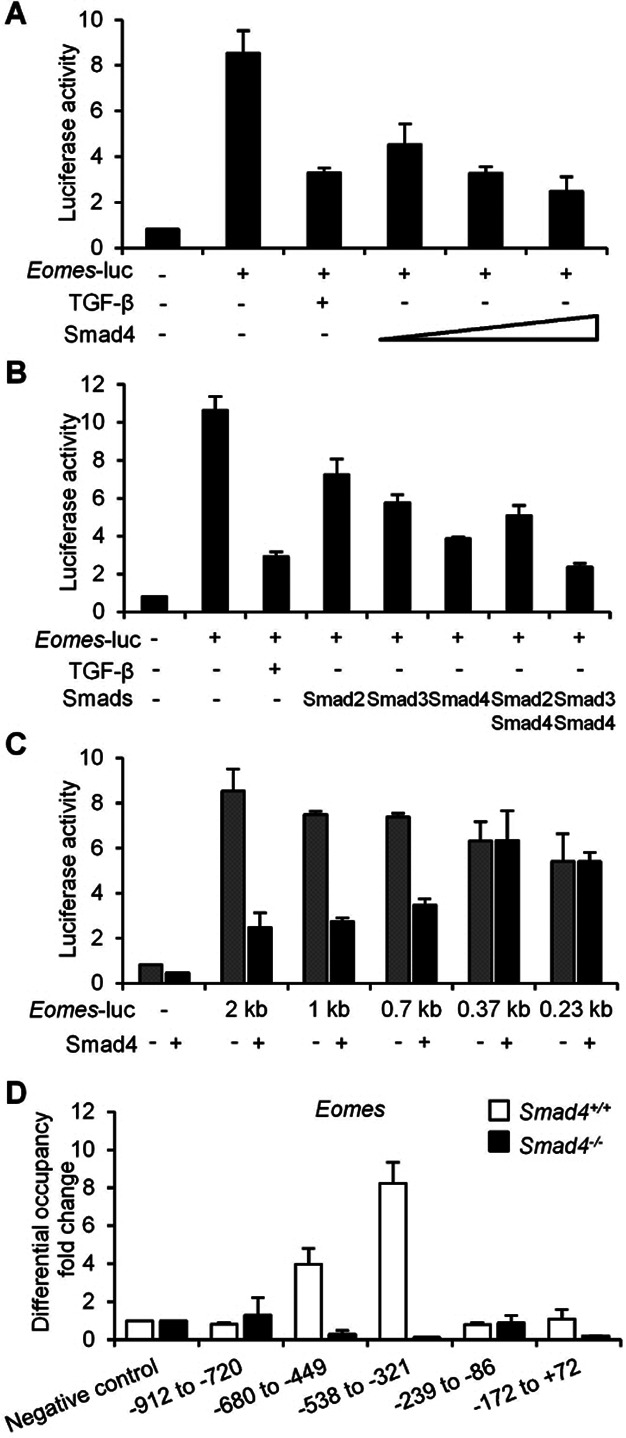
Smad4 binds to the *Eomes* promoter to repress transcription **A–C.** C57BL/6 CD8^+^ cells stimulated with anti-CD3/CD28 for 3 days were transfected with the *Eomes* luciferase reporter construct with various dosages of Smad4, Smad2, Smad3 and Smad4 or the various truncated *Eomes* luciferase reporter constructs with or without Smad4. TGF-β1 was treated as a control.**D.** CD8^+^ cells from *Cd4Cre;Smad4*^*+/+*^ and *Cd4Cre;Smad4*^*fl/fl*^ mice were stimulated with anti-CD3/CD28 for 3 days, lysed and immunoprecipitated with either anti-Smad4 or rabbit IgG. Bound DNA was measured by qPCR using primers specific to the *Eomes* promoter. Graphs show mean + SEM (*n* = 3). Differential occupancy fold changes from four independent experiments are shown.

We next assessed the direct transcriptional regulation of the *Eomes* gene by Smads in CD8^+^ T cells using luciferase assays. Smad4 inhibited Eomes-luc activity (−2.0 kb) in a dose dependent manner (Fig 9A). Smad4 inhibited Eomes-luc activity to the same level as TGF-β1 (5 ng/ml), whereas Smad3 inhibited it to a lesser degree, and Smad2 was inactive (Fig 9B). Smad2 reversed, whereas Smad3 further enhanced, the inhibitory effect of Smad4 on Eomes-luc activity (Fig 9B). Thus, Smad4 is the main repressor and Smad3 is the corepressor of the *Eomes* gene.

To screen the Smad4 binding regions in the *Eomes* promoter, we generated serial truncated luciferase reporter constructs (Fig 9C). Inhibition of luciferase activity by Smad4 was abolished in a −0.37 kb reporter construct, whereas a −0.7 kb construct remained susceptible to Smad4 inhibition (Fig 9C), indicating that the Smad4 binding sites are located between −0.37 kb and −0.7 kb. Screening Smad-binding sequence, CAGAC (Massague et al, 2005) by ChIP showed that Smad4 bound to (−680 to −499) and (−538 to −321) in the *Eomes* proximal promoter (Fig 9D). Specificity of Smad4 pull-down was confirmed by completely abolished enrichment at these sites in *Smad4*^−/−^ CD8^+^ T cells (Fig 9D). Thus, Smad4 binds to the proximal promoter of the *Eomes* gene, thereby repressing its transcription in CD8^+^ T cells.

## DISCUSSION

It has been well documented that systemic TGF-β antagonism mainly targets CD8^+^ T cells in cancer (Nam et al, 2008) and selective blockade of TGF-β signalling in pan T cells or CD8^+^ T cells is sufficient to eradicate tumours (Donkor et al, 2011; Gorelik & Flavell, 2001; Zhang et al, 2005). Meanwhile, the precise molecular mechanisms whereby TGF-β antagonists enhance T-cell-mediated anti-tumour immunity remain unknown. Here, we show that ALK5 inhibition by LY-2157299 and a novel ALK5 inhibitor, EW-7197 induced ubiquitin-mediated degradation of Smad4 protein in immune cells, most profoundly in CD8^+^ T cells. However, pharmacologic ALK5 inhibition *in vitro* and *in vivo* did not affect the total expression levels of Smad4 protein in melanoma cells. TGF-β signalling pathway is controlled by ubiquitin protein modification (De Boeck & ten Dijke, 2012; Izzi & Attisano, 2004). Various E3 Ub ligases, such as Smurfs, WWP1, NEDD4-2, CHIP and SCF target Smad4 for degradation to negatively regulate TGF-β signalling (Li et al, 2004; Moren et al, 2005; Wan et al, 2004). Jab1 antagonizes TGF-β function by inducing uniquitin-mediated degradation of Smad4 (Wan et al, 2002). Although R-Smads are also controlled by ubiquitin-mediated degradation (De Boeck & ten Dijke, 2012; Izzi & Attisano, 2004), ALK5 inhibitors did not reduce R-Smads. Ubiquitnation of proteins by E3 ligases has emerged as an indispensable signalling pathway that regulates T-cell tolerance (Paolino & Penninger, 2009). We investigated the possible involvement of Smurf, because IL-7 modulates TGF-β signalling via Smurf2 activity in CD8^+^ T cells (Pellegrini et al, 2009). However, Smurf1/2 were found to be irrelevant to Smad4 degradation by ALK5 inhibition in CD8^+^ T cells (Supporting Information Fig S8). Future studies are required for elucidating precise mechanisms whereby ALK5 inhibition induces ubiquitin-mediated degradation of Smad4 specifically in CD8^+^ T cells.

We observed that TGF-β antagonism by ALK5 inhibition blocked the intact intracellular TGF-β signalling through R-Smads and Smad4 in B16 melanoma cells, and yet they were resistant to TGF-β. By contrast with B16 melanoma cells, C-terminally unphosphorylated R-Smads by ALK5 inhibition were still capable of translocating into nuclei in the dLNs, especially in CD8^+^ T cells in melanoma-bearing mice. Lymphocytes are activated with T/B-cell receptors together with co-stimulatory molecules, and/or cytokine receptors, which activate the signalling pathways through serine/threonine kinases, such as MAPKs and PKC. These kinases phosphorylate the linker regions or MH1 domains of R-Smads (Chang et al, 2011; Heldin & Moustakas, 2012; Matsuzaki, 2013). Future studies are required to elucidate the roles of R-Smad phosphorylation in the linker regions or MH1 domains and the mechanisms of R-Smad nuclear retention when Smad4 is downregulated in lymphocytes.

EW-7197 and T-cell-specific Smad4 gene targeting enhanced anti-tumour CTL responses with specific upregulation of Eomes in melanoma-bearing mice. CD8^+^ T cells lacking the *Smad2/3/4* genes and the promoter analyses showed that Smad4 was the main repressor of the *Eomes* gene. As reported that Smad2 and Smad3 had distinct regulatory effects in epithelial cells and Th17 cells despite of their high homology (Brown et al, 2007; Martinez et al, 2009, 2010), Smad3, but not Smad2 had an additive effect on transcriptional repression of Eomes by Smad4. By contrast, it has been reported that TGF-β suppresses Eomes via Smad2/3-independent, JNK-dependent signalling in Th17 induction (Ichiyama et al, 2011; Takimoto et al, 2010). Discrepancy between their reports and our study might be due to several reasons: TGF-β signalling pathways to suppress Eomes might be different between CD4^+^ and CD8^+^ T-cell effector subsets, Smad4 was not investigated in their reports, they used T cells from *LckCreSmad2*^*fl/fl*^*Smad3*^−/−^ (Smad2/3-DKO) or *LckCreSmad2*^*fl/fl*^*Smad3*^*+/−*^ (Smad2cKO/Smad3hetero) mice, so that Smad4 alone or Smad4 and haploid expression of Smad3 could still transduce TGF-β signalling to repress the *Eomes* gene according to our findings (Fig 9A and B). They speculated JNK-dependent, Smad2/3-independent pathway from the similar attenuating effect of ALK5 inhibitor, SB431542 and JNK inhibitor, SP600125 on Eomes repression in T cells stimulated with TCR and TGF-β. However, specificity of ALK5 inhibitors for Smad-mediated TGF-β signalling pathway (Akhurst & Hata, 2012; Flavell et al, 2010; Hawinkels & ten Dijke, 2011; Jin et al, 2011) and cooperation of Smad3 and Smad4 with c-Jun/c-Fos to mediate TGF-β-induced transcription (Zhang et al, 1998) suggest that both Smad3/4 and JNK pathways are involved in TGF-β-induced Eomes suppression.

Although TGF-β suppresses the cytolytic genes and IFN-γ by a mechanism involving R-Smads and ATF1 (Thomas & Massague, 2005) and Eomes as well as IFN-γ and cytolytic molecules are regulated by Runx3 (Cruz-Guilloty et al, 2009), Smad4 did not regulate IFN-γ production by CD8^+^ T cells in our model. Because Runx3 is known to cooperate with Smad3/4 to regulate target genes (Pardali et al, 2000; Zhang & Derynck, 2000), Smad4 might be required for Runx3 to regulate IFN-γ, but not Eomes and cytolytic molecules. Recent findings revealed the melanoma-promoting effects of IFN-γ (Cho et al, 2011; Zaidi et al, 2011). Thus, the ability to upregulate CTL functions without affecting IFN-γ would prove safety and efficacy of ALK5 inhibition for anti-melanoma therapy. However, cell-specific regulatory mechanisms of IFN-γ and T-bet by TGF-β remain to be determined because TGF-β suppresses IFN-γ and T-bet via MAPK-dependent, Smad3-independent signalling in CD4^+^ T cells (Park et al, 2007), whereas TGF-β suppresses IFN-γ and T-bet via Smad2/3/4-mediated signalling in NK cells (Tinoco et al, 2009).

Efficacy of ALK5 inhibition on a relatively immunogenic B16 melanoma model depends fully on CD8^+^ T cells because deletion of CD8^+^ T cells resulted in 100% loss in the efficacy of EW-7197 on tumour progression (Fig 6B and E). Although NK cell deletion showed partial reduction in the efficacy of EW-7197 (Fig 6D and E), ALK5 inhibition did not upregulate Eomes in NK cells (data not shown). In a relatively non-immunogenic 4T1 model, anti-TGF-β antibodies suppress metastasis via cooperative effects on multiple cellular components: CD8^+^ T cells, NK cells and tumour cells (Nam et al, 2008). Therefore, immunogenicity of tumours is presumably the crucial factor to affect the potency of TGF-β antagonism on the specific cellular targets in anti-tumour therapy; nevertheless enhancement of CD8^+^ T-cell mediated anti-tumour immune response is the main outcome of TGF-β antagonism even in the non-immunogenic tumour.

TGF-β also regulates effector CD4^+^ T-cell subsets (Li et al, 2006). However, downregulation of Smad4 by neither ALK5 inhibition nor T-cell-specific gene targeting affected any CD4^+^ T-cell subsets in melanoma-bearing mice. Although TGF-β inhibits T-bet (Park et al, 2007) and Eomes (Narayanan et al, 2010) in Th1 cells, Smad4 downregulation had no effect on T-bet and Eomes in CD4^+^ T cells. Similarly with our model, systemic TGF-β antagonism by IN-1130, one of the prototype ALK5 inhibitors in the same structural family as EW-7197 ameliorates experimental autoimmune encephalomyelitis by local actions without affecting systemic peripheral immune reactions including the generation of Th17 (Luo et al, 2007). Concerning Tregs, one of the major suppressors of anti-tumour immune surveillance (Flavell et al, 2010), Smad4-independent development of Tregs in our model and Smad2/3-independent development of nTregs *in vivo* (Gu et al, 2012) indicate that Treg development is Smad-independent. Thus, systemic TGF-β antagonism seems to target the disease-specific major pathogenic immune effector cells in inflammatory lesions without affecting systemic immune homeostasis. Further investigation is required to determine the distinct targets of systemic TGF-β antagonism in various diseases.

One major concern for pharmacologic Smad4 downregulation is the possibility of the gastrointestinal inflammation and spontaneous carcinogenesis that was observed in mice in which Smad4 was targeted in T-cell-specific and systemic inducible routes (Hahn et al, 2011; Karlsson et al, 2007; Kim et al, 2006). However, T-cell-specific Smad4 deletion by *Cd4Cre* recombinase transgene with C57BL/6 background showed a normal phenotype at least by 6 months of age in our SPF facilities. Moreover, even the complete Smad4 knockout in T cells took time to develop carcinogenesis (Hahn et al, 2011; Kim et al, 2006). Considering the short *in vivo* half-life of EW-7197 and maintenance of normal immune homeostasis by lifetime exposure to a soluble TGF-β antagonist (Yang et al, 2002), the risk of gastrointestinal inflammation and carcinogenesis by temporal or intermittent prescription of EW-7197 is expected to be low.

Several ALK5 inhibitors are currently at pre-clinical and clinical stages for various cancers including melanoma (Akhurst & Hata, 2012; Flavell et al, 2010; Hawinkels & ten Dijke, 2011; Mohammad et al, 2011). Because orally administered EW-7197 was more efficacious than LY-2157299 (75 mg/kg bid) against melanoma at a dose as low as 2.5 mg/kg daily, EW-7197 is the good candidate as the next generation ALK5 inhibitor for anti-melanoma therapy.

In summary, ALK5 inhibitors have a potent therapeutic efficacy against melanoma by novel mechanisms: inducing the ubiquitin-mediated degradation of Smad4, thereby relieving suppressive effects of TGF-β on Eomes in CTLs.

## MATERIALS AND METHODS

### Mice

Mice homozygous for a conditional *Smad4* allele (*Smad4*^*loxp/loxp*^; Kim et al, 2006) and *Smad2* allele (*Smad2*^*loxp/loxp*^; Liu et al, 2004) were bred with *Cd4Cre* recombinase transgenic mice (Lee et al, 2001) for the selective deletion of the genes flanked by loxP targeting sequences in thymocytes at the double positive stage. They were backcrossed to C57BL/6 (The Jackson Laboratory) for eight generations. *Smad3*^*+/−*^ mice (Yang et al, 1999) were backcrossed to C57BL/6 for two generations because the probability of *Smad3*^−/−^ dropped to <1% in our facility due to the increased embryonic lethality. All experiments used age-matched mice. All animals were maintained in a SPF environment and used in experiments according to the ethical guidelines for animal experiments and the safety guidelines for gene manipulation experiments at University of Tsukuba, Japan, Tokyo Medical University, Japan, Gachon University, Korea and Konkuk University, Korea under approved animal study protocols.

### B16 melanoma model and treatment with ALK5 inhibitors

Parental B16F1 (B16) cells and B16 cells transfected with the FG12 lentiviral vector expressing green fluorescent protein (GFP) were cultured in DMEM media (Gibco) containing 10% heat-inactivated FBS (Gibco) supplemented with 1% penicillin and streptomycin. Mice (8–12 weeks) were subcutaneously injected with GFP-expressing B16 cells (4 × 10^4^) into the left footpads or with GFP-expressing B16 cells (2 × 10^5^) into the left lower abdomen. Tumour size was measured by a caliper everyday. Volume was calculated by ([short diameter]^2^ × long diameter)/2 (Pedroza-Gonzalez et al, 2011). Resected tumour was weighed. Tumours, dLNs (left axillary, brachial and inguinal), non-dLNs and spleens were harvested for evaluation. EW-7197 (2.5 mg/kg daily) or LY-2157299 (75 mg/kg bid) dissolved in artificial gastric fluid formulation (vehicle; ddH_2_O 900 ml, conc. HCl 7 ml, NaCl 2.0 g, pepsin 3.2 g) was given orally by feeding needle to mice from 4 days after inoculation. To delete CD8^+^, CD4^+^ T cells or NK cells, mice were intraperitoneally injected with LEAF purified anti-CD4 (GK1.5), anti-CD8 (53–6.7; 150 μg/mouse; BioLegend) or anti-asialo GM1 (20 μl/mouse; Wako) antibody on Day −4, 0, 7 and 14 of melanoma inoculation (Day 0). Rat IgG2a κ (150 μg/mouse; BioLegend) was used as a representative control.

### Isolation of TILs

Melanoma infiltrating T cells were isolated following the reported protocol (Watkins et al, 2012). Briefly, B16 melanoma tumours measuring up to 250 mm^2^ were cut into small pieces and incubated in RPMI medium supplemented with 5% FBS, Collagenase type I (200 U/ml; Sigma–Aldrich) and DNase I (100 μg/ml; Sigma–Aldrich) for 30 min at 37°C. Cells were enriched by density gradient centrifugation using Ficoll-Paque 1.084 (GE Healthcare).

### Flowcytometry

Single cell suspensions were prepared from spleens and LNs. After blocking Fc receptors by anti-mouse CD16/CD32, cells were stained with APC-Cy7-anti-CD4, Pacific blue-anti-CD8, PE-anti-CD19, APC-anti-CD11b, PE-anti-Gr-1, APC-anti-CD11c, APC-anti-DX5 and APC-anti-CD45. To measure cytokine production, dLN cells were stimulated with PMA (2.5 ng/ml; Sigma–Aldrich) and ionomycin (2.5 ng/ml; Sigma–Aldrich) with GolgiPlug (BD Pharmingen) for 1–4 h. Cells were fixed, permeabilized with a Cytoperm/Cytofix Kit (eBiosciences). For intracellular staining, PE-anti-T-bet, APC-anti-Eomes, PE-anti-GATA3, PE-anti-RORγt, PE-Cy7-anti-FoxP3, PE-anti-perforin, APC-anti-granzyme B and PE-Cy7-anti-IFN-γ were used. Antibodies were obtained from BD Pharmingen and eBiosciences. The samples were acquired by LSRII (BD Bioscience) and analysed using the FlowJo software (Tree Star).

### Cytotoxicity assay

CD8^+^ cells enriched from the spleens of melanoma-bearing mice by mouse T-cell enrichment column (R&D Systems) and magnetic activated cell separation (MACS) using CD8a microbeads (Miltenyi Biotec) as effector cells (0, 5 × 10^4^, 1 × 10^5^, 2 × 10^5^) were co-cultured with B16 cells (2 × 10^3^) as target cells in U-bottom 96-well plates (NUNC) for 72 h. Annexin V/propidium iodide (PI) (BD Pharmingen) staining in the large FSC/SSC GFP^+^ gate was determined by LSRII.

### Histology

Tumours were harvested and fixed in 10% neutral-buffered formalin. Fixed samples were then dehydrated with 70% ethanol, embedded in paraffin and sectioned at 3 μm. For immunohistochemistry, sections were stained with anti-CD8, anti-Eomes, anti-phospho-Smad3 (Abcam), anti-phospho-Smad2 (Cell Signaling Technology) and anti-Smad4 (Santa Cruz) antibodies in antibody diluent overnight and stained with HRP/DAB method (Dako). Sections were then counterstained with haematoxylin. Slides were observed using an optical microscope, Imager Z1 (Carl Zeiss). Expression of Smad4 in B16 melanomas was quantified using ImageJ software (Image Processing and Analysis in Java, National Institutes of Health, USA).

### Immunocytochemistry

Freshly isolated spleen, dLN cells from melanoma-bearing mice were fixed on the slides by 3.7% formaldehyde. PLA was performed using the Duolink II Fluorescence kit (OLINK) according to the manufacturer's protocol using the rabbit antibodies against: Smad2, Smad3, phospho-Smad2, phospho-Smad3, Smad4 and mouse antibodies against: Smad2/3 and ubiquitin (Cell Signaling Technology, BD Bioscience). In order to detect and quantify endogenous Smad protein expression, single recognitions were performed for each Smad protein by using a combination of target specific rabbit primary antibody and its respective secondary antibodies conjugated with oligonucleotides (PLA probe anti-rabbit PLUS and PLA probe anti-rabbit MINUS). In order to detect close proximity of two proteins (<40 nm), double recognitions were performed by using a combination of two target specific primary antibodies raised in different species (rabbit anti-Smad2 and mouse anti-ubiquitin, rabbit anti-Smad3 and mouse anti-ubiquitin, rabbit anti-Smad4 and mouse anti-ubiquitin, mouse anti-Smad2/3 and rabbit anti-Smad4) and their respective secondary antibodies conjugated with oligonucleotides (PLA probe anti-rabbit PLUS and PLA probe anti-mouse MINUS). Technical negative controls using one primary antibody for each combination of double recognitions showed no background signals. After incubation of the slides with blocking solution for 30 min at 37°C, they were incubated with primary antibodies diluted in the antibody diluent overnight at 4°C, in PLA probe solution for 1 h at 37°C and in ligation-ligase solution for 30 min at 37°C with washing with wash buffer A in the interim of each step. The slides were incubated in amplification-polymerase solution for 100 min at 37°C and then washed in wash buffer B. To co-stain CD8, rat anti-CD8 antibody was added in the antibody diluent with primary antibodies for PLA and the slide were incubated with Alexa Fluor 488 conjugated anti-rat IgG (Abcam) after washing in wash buffer B. Nucleus was stained with DAPI. Then, the slides were dried at room temperature in the dark. Slides were observed using a confocal microscope, LSM700 (Carl Zeiss). PLA signals were quantified using BlobFinder software (Centre for Image Analysis, Uppsala University).

### T-cell stimulation *in vitro*

Suspended whole dLN cells (1 × 10^6^/ml) of melanoma-bearing mice were labelled with CFSE (Invitrogen) for stimulation with H-2D^b^ human gp100 peptide (5 μg/ml, Medical and Biological Laboratories). After 5 days, CFSE dilution of CD8^+^ gate was analysed by flowcytometry. CD8^+^ or CD4^+^ cells (1 × 10^6^ cells/ml) enriched from spleens and LNs by MACS using CD4 or CD8a microbeads were stimulated with plate-coated anti-CD3 (1 µg/ml) and soluble anti-CD28 (3 µg/ml) antibodies (BD Pharmingen) with or without TGF-β1 (5 ng/ml; R&D Systems), EW-7197 (0, 0.5, 1.0, 2.0, 5.0 μM), MG-132 (0.5 μM; Sigma–Aldrich) for 3 days in 50 µM 2-mercaptoethanol containing RPMI 1640 media in 24-well plates (NUNC).

**The paper explained**

PROBLEM:

Among transforming growth factor-β (TGF-β) antagonists to intervene with excessive TGF-β signalling activity in cancer, small molecule ALK5 inhibitors specifically inhibit R-Smad phosphorylation by TGF-β type I receptor. Although CD8^+^ T-cell is the major cell compartment targeted by TGF-β antagonism, precise mechanisms whereby ALK5 inhibitors enhance anti-tumour cytotoxic T-lymphocyte (CTL) activity remain largely unknown.

RESULTS:

We demonstrate that a novel ALK5 inhibitor, EW-7197 (2.5 mg/kg daily) or a representative ALK5 inhibitor, LY-2157299 (75 mg/kg bid) suppress the progression of mouse B16 melanoma with enhanced CTL responses. Notably, systemic ALK5 inhibition in melanoma-bearing mice induces ubiquitin-mediated degradation of Smad4 mainly in CD8^+^ T cells on top of systemic inhibition of R-Smad phosphorylation in immune cells as well as in melanoma. Consistently, T-cell-specific Smad4 deletion is sufficient to suppress melanoma progression. We further identify eomesodermin (Eomes), the essential T-box transcription factor for CTL functions, as a specific target repressed by TGF-β via Smad4 in CD8^+^ T cells.

IMPACT:

Our study reveals novel mechanisms whereby ALK5 inhibitors enhance anti-melanoma immunity on top of their direct systemic inhibitory effect on R-Smad phosphorylation: CD8^+^ T-cell-specific ubiquitin-mediated degradation of Smad4 and derepression of Eomes, thereby enhancing CTL functions. The potent efficacy of a novel orally bioavailable ALK5 inhibitor, EW-7197 against melanoma may improve melanoma management in the clinic.

### Western blotting and *in vivo* ubiquitination assay

Cells lysed with lysis buffer (PBS containing 0.5% Triton X-100, 20 mM HEPES (pH 7.4), 150 mM NaCl, 12.5 mM β-glycerol phosphate, 1.5 mM MgCl_2_, 10 mM NaF, 2 mM DTT, 1 mM NaOV, 2 mM EGTA, 1 mM PMSF and protease inhibitor cocktail) were electrophoresed on 10% SDS–polyacrylamide gel and transferred to PVDF membrane, and probed with antibodies against phospho-Smad2, phospho-Smad3 (Abcam), Smad2, Smad3, Smad4 and β-actin (Santa Cruz). Blots were visualised using an electrochemiluminescence kit (GE Healthcare). Ubiquitinated Smad4 in CD8^+^ dLN cells was detected using an UbiQapture-Q kit (Enzo Life Sciences) according to the manufacturer's protocol. Isolated CD8^+^ dLN cells by MACS were pooled (8 × 10^6^ cells from 10 mice/sample) for lysis. Equal amounts of protein of CD8^+^ LN cells were incubated overnight at 4°C with the UbiQapture-Q matrix beads, which capture mono/poly-ubiquitinated proteins. The matrix was then washed and the ubiquitin–protein conjugates were eluted by addition of PBS and denaturating buffer. Samples were quenched by incubation for 15 min at 4°C and then denaturated by heating at 95°C for 10 min. Proteins were eluted in Laemli's sample buffer, and subsequently processed for Western blotting with anti-Smad4 antibody (Cell Signaling) and the ubiquitin-conjugate antibody supplied by the kit. Ubiquitinated Smad4 in cultured CD8^+^ T cells was detected as previously described (Lee et al, 2011). Briefly, CD8^+^ cells stimulated with anti-CD3/CD28 antibodies (2 × 10^7^ CD8^+^ cells/sample) were harvested and non-covalent protein interactions were dissociated with 1% SDS and boiling for 10 min. Samples were diluted 10 times with lysis buffer and subsequently suspended using a 1 ml syringe. The samples were cleared by centrifugation at 16,000*g* for 10 min. Lysates were incubated with protein A/G agarose beads and with anti-Smad4 antibody (Santa Cruz) at 4°C for 12–16 h. The beads were washed three times with lysis buffer and immunoprecipitates were separated from the beads by adding 2× sample buffer and boiled. SDS–PAGE-separated immunoprecipitates were transferred onto PVDF membranes. The membranes were denatured with denaturation buffer containing 6 M guanidine chloride, 20 mM Tris (pH 7.5), 100 mM PMSF and 5 mM β-mercaptoethanol at 4°C for 30 min and washed three times with TBST. The membranes were blocked with 5% BSA and incubated with anti-ubiquitin-HRP antibody (Biomol).

### Quantitative RT-PCR

Total RNA extracted using Trizol (Invitrogen) was reverse transcribed with a cDNA Reverse Transcription Kit (Invitrogen). Real-time quantitative PCR (triplicate/sample) was performed using an ABI 7900 Analyzer with SYBR Green Master Mix (Applied Biosystems) with the primers listed in Supporting Information Table 1.

### Luciferase assay

The proximal promoter regions of Eomes were generated by PCR from C57BL/6 genomic DNA using the primers listed in Supporting Information Table 2. Products were verified by sequencing and subcloned into the pGL4 luciferase vector (Promega) using *Kpn*I and *Xho*I sites, *Xho*I and *Hind*III sites respectively. The resulting constructs were transfected into *in vitro*-stimulated CD8^+^ cells along with control thymidine kinase-pRL *Renilla* plasmid (Promega) using Amaxa Nucleofector II (Lonza). Flag-tagged Smad4, with or without Flag-tagged Smad2 or Smad3, or an empty pcDNA3 plasmid were co-transfected. At 6 h after transfection, cells were restimulated with anti-CD3 and anti-CD28 antibodies as described above for 4 h and lysed for luminometer measurements.

### Chromatin immunoprecipitation (ChIP)

Chromatin was prepared from 1 × 10^7^ CD8^+^ cells isolated from C57BL/6, *Cd4Cre;Smad4*^*+/+*^ and *Cd4Cre;Smad4*^*fl/fl*^ mice stimulated for 3 days as described above. Immunoprecipitation was performed with anti-Smad4 antibody and rabbit IgG using a ChIP Kit (Cell Signaling). Immunoprecipitated DNA released from the cross-linked proteins was quantified by real-time quantitative PCR with the primers listed in Supporting Information Table 3 and normalized to input DNA.

### Statistics

Statistical analyses were performed using analysis tools provided on the VassarStats statistical computation site (http://vassarstats.net/). Data were analysed using the two-tailed unpaired Student's *t*-test and two-way ANOVA test. A *p*-value <0.05 was considered to indicate statistical significance.

## Supplementary material

As a service to our authors and readers, this journal provides supporting information supplied by the authors. Such materials are peer reviewed and may be re-organized for online delivery, but are not copy-edited or typeset. Technical support issues arising from supporting information (other than missing files) should be addressed to the authors.

Review Process File

**Figure S1.** Detection of lymph node metastsis.

**Figure S2.** TGF-β does not affect apoptosis and cell cycle of B16 cells.

**Figure S3.** Oral administration of EW-7197 suppresses melanoma and LN metastases with enhanced CTL activity.

**Figure S4.** Oral administration of EW-7197 inhibits TGF-β signalling and downregulates Smad4 in spleen cells of melanoma-bearing mice.

**Figure S5.** Oral administration of LY-2157299 inhibits TGF-β signalling and downregulates Smad4 in dLN cells of melanoma-bearing mice.

**Figure S6.** LY-2157299 induces Smad4 ubiquitination in dLN cells of melanoma-bearing mice.

**Figure S7.** R-Smads are not ubiquitinated in dLN cells of EW-7197-treated melanoma-bearing mice.

**Figure S8.** Smurf1 and Smurf2 are not involved in degradation of Smad4 by ALK5 inhibition.

**Figure S9.** T-cell-specific Smad4 deletion suppresses melanoma and LN metastases with enhanced CTL activity.

**Figure S10.** Upregulation of Eomes in CD8^+^ T cells by ALK5 inibition or T-cell-specific Smad4 deletion.

**Figure S11.** Eomes is not expressed in CD4^+^ cells in the dLNs of melanoma-bearing mice.

**Figure S12.** Characterization of TILs.

**Figure S13.** Deletion of CD8^+^ T cells, CD4^+^ T cells, NK cells *in vivo*.

**Figure S14.** T-cell-specific Smad4 deletion.

**Figure S15.** Effect of Smad4 on PMA/ionomycin-stimulated CD8^+^ T cells.

**Table S1.** Primer sequences for quantitative RT-PCR.

**Table S2.** Primer sequences for the proximal promoter regions of Eomes.

**Table S3.** Primer sequences for ChIP.
