# Supplementary material for: Activin receptor-like kinase5 inhibition suppresses mouse melanoma by ubiquitin degradation of Smad4, thereby derepressing eomesodermin in cytotoxic T lymphocytes
Source: EMBO Mol Med. 2013 Oct 11;5(11):1720–39. doi: 10.1002/emmm.201302524 (PMC3840488; doi:10.1002/emmm.201302524)
Supplement: Supplementary file 1 [file emmm0005-1720-SD1.pdf]

EMBO Molecular Medicine

Activin receptor-like kinase5 inhibition suppresses mouse melanoma by ubiquitin degradation of Smad4, thereby derepressing Eomesodermin in cytotoxic T lymphocytes

Jeong-Hwan Yoon, Su Myung Jung, Seok Hee Park, Mitsuyasu Kato, Tadashi Yamashita, In-Kyu Lee, Katsuko Sudo, Susumu Nakae, Jin Soo Han, Ok-Hee Kim, Byung-Chul Oh, Takayuki Sumida, Masahiko Kuroda, Ji-Hyeon Ju, Kyeong Cheon Jung, Seong Hoe Park, Dae-Kee Kim, and Mizuko Mamura

Corresponding author: Mizuko Mamura, Tokyo Medical University

---

Review timeline:

Submission date:	24 January 2013
Editorial Decision:	28 February 2013
Revision received:	19 July 2013
Editorial Decision:	31 July 2013
Revision received:	20 August 2013
Editorial Decision:	23 August 2013
Revision received:	25 August 2013
Accepted:	06 September 2013

Transaction Report:

(Note: With the exception of the correction of typographical or spelling errors that could be a source of ambiguity, letters and reports are not edited. The original formatting of letters and referee reports may not be reflected in this compilation.)

Editors: Céline Carret / Roberto Buccione

1st Editorial Decision 28 February 2013

Thank you for the submission of your manuscript to EMBO Molecular Medicine. We have now heard back from the two referees whom we asked to evaluate your manuscript. Although the referees find the study to be of potential interest, they also raise a number of concerns that need to be addressed in a major revision of your manuscript.

As you will see from the reports below, concerns from both referees are mainly of an experimental nature, but they do insist that better quality and more controlled assays should be performed. In addition, mechanistic insight should be provided. Referee 2 would also like to see a better integration of the results within the accepted literature.

In our view the suggested revisions would render the manuscript much more compelling and interesting to a broad readership. We therefore hope that you will be prepared to undertake the recommended experimental revision.

Please note that it is EMBO Molecular Medicine policy to allow a single round of revision only and that, therefore, acceptance or rejection of the manuscript will depend on the completeness of your responses included in the next, final version of the manuscript.

As you know, EMBO Molecular Medicine has a "scooping protection" policy, whereby similar findings that are published by others during review or revision are not a criterion for rejection. However, I do ask you to get in touch with us after three months if you have not completed your revision, to update us on the status. Please also contact us as soon as possible if similar work is published elsewhere.

I look forward to receiving your revised manuscript.

***** Reviewer's comments *****

Referee #1 (Comments on Novelty/Model System):

This is a highly interesting paper, however some techniques appear not properly controlled. These results need to be validated and consolidated.

Referee #1 (Remarks):

In this report inhibition of TGF-beta type I receptor kinase activity is shown to suppress mouse B16 melanoma progressionby inducing the degradation of Smad4 in CD8+ T-cells. Consistent with this finding, T-cell specific deletion of Smad4 inhibits melanoma progression. Furthermore, T-box transcription factor Eomesodermin was identified as specific target that is repressed by TGF-beta in a Smad3/4-dependent manner in CD8+ cells.

In general the experiments are well performed and conclusions are supported by data.

Specific comments:

1. Poximity ligation (PLA) experiments are shown in Fig. 2 and 3. More experimental detail is needed. At present its unclear how these experiments were performed and controlled. Were no signals obtained when one antibody was added? Differential PSmad2 PLA data (and no effect on total level of Smad2 and Smad3) need to be consolidated with Western blot analysis.
In Figure 3a: When positive signals are obtained with Smad4 antibody and ubiquitin antibody, this does not (necessarily) mean that Smad4 is ubiquitinated. PLA only demonstrates that the proteins are in close proximity. Is there a PLA signal for Smad2 and ubiquitin?

2. The ALK5 inhibition induced ubiquitination of Smad4 as shown in Fig. 3B data do not look convincing. Smad4 ubiquitination ladder can not be seen. Most of the Ub-Smad4 have a lower molecular weight than Smad4?
It is unclear how this assay was performed. It needs to be done under denaturing conditions: otherwise no distinction can be made between Smad4 proteins that have been covalently modified by ubiquitin and protents that interact with Smad4 (noncovalently) that are ubiquitinated. This assay needs to be repeated and performed in a different manner.

3. The mechanism for selective Smad4 degradation in CD8 cells is unclear. The authors could investigate the possible involvement of Smurf.

4. Is Eome protein differentially expressed in CD8 cells in vivo upon Smad4 deletion in CD8+ T cells or upon ALK5 inhibition?

Referee #2 (Comments on Novelty/Model System):

The model chosen is fine but would have been improved by analysis of TIL rather than LN cells, purification of specific immune subsets by flow cytometry prior to gene expression analysis, and more detailed proof that the anti-tumor effects are fully due to the impact on CD8 T-cells.

Referee #2 (Remarks):

The manuscript "Activin receptor-like kinase5 inhibition suppresses mouse melanoma by ubiquitin degradation of Smad4, thereby derepressing Eomesodermin in cytotoxic T lymphocytes" details a complex mechanism by which ALK5 inhibition blocks elements of TGF-b signalling leading to improved anti-tumor CTL responses.

Overall this is a good manuscript worthy of publication; however, there are few items of concern generally:
1) A more concrete demonstration that the effects of the inhibitors on restricting tumor growth are fully attributable to their effects on the CD8 T-cell compartment are needed. Minimally, immunocompromised (RAG-KO, Nude etc) mice bearing tumors could be treated showing no change in tumor growth. An ideal experiment might be to antibody deplete CD8, CD4, NK1.1 cells and show in each case how much of the efficacy of the inhibitors is lost.
2) The authors need to discriminate between increases in cytotoxic or t-box transcription factors at the gene expression level in the overall node vs. on a per cell basis. In a few cases they insinuate these have increased "in CD8 cells in the dLN" when they have shown that the numbers of CD8 cells in the node have been increased substantially - thus we don't know if the higher Granzyme RNA (for example) is due to more CD8 cells, a higher per cell expression of Granzyme, or both. The flow plots generally following the RNA analysis are helpful and show changes on a per cell basis. In the future, the authors may wish to sort pure populations of CD8 cells from the dLN for gene expression analysis which would allow them to discriminate between changes in per cell expression level vs. changes in the density of CD8 cells in the overall LN. We hope the authors in the future will also consider analyzing TIL instead of dLN cells as the results can often be quite distinct.
3) The authors assert that degradation of Smad4 leads to de-repression of Eomesodermin; however, Ichiyama and Yoshimura (2011) published that TGF-b mediated repression of Eomes was through a JNK driven, Smad-independent (at least Smad2 and 3 independent) pathway. The authors need to more thoroughly describe how their data integrates with the published pathway.
4) Lack of induction of IFN-g in concert with Eomes induction is unusual given that Eomes (+Runx3) drives IFN-g expression moreso than it does Granzyme B. I believe the lack of IFN-g induction seen by the authors in Figure 6 and elsewhere may be due to their activation of their T-cells in vitro using aCD3/aCD28 beads. These beads are the best reagents for measuring proliferative changes; however, PMA/Ionomycin stimulation is generally superior for studying in vitro IFN-g production.

Minor Specifics:
In the abstract the sentence starting with "Notably, progression....melanoma-bearing mice" is a run-on and unclear and should be re-worked.
Calling Eomes "the essential T-box transcription factor for CTL functions" in the abstract is overstated given that Intelkofer and Reiner showed that Eomes-/- CTL are fully functional.
In the introduction "heteromeric" is a not word. Perhaps heterodimeric?
It would be nice to hear at least a speculative explanation for why Alk5 inhibition leads to Smad4 downregulation in CD8 T-cell but not CD4 T-cells or B16 tumor cells as this mechanism is critical to the postulated effect of the inhibitors.

1st Revision - authors' response 19 July 2013

We would like to thank the editor and referees for their precious comments. We described the details of the experimental procedures and performed the suggested experiments. We have included the comments by the referees in bold, which are followed by our response.

Referee 1.

Instructive comments and suggestions by the referee led us to elaborate on the detection of endogenous ubiquitinated Smad4 in $CD8^+$ T cells.

1. Poximity ligation (PLA) experiments are shown in Fig. 2 and 3. More experimental detail is needed. At present its unclear how these experiments were performed and controlled. Were no signals obtained when one antibody was added?

We clarified the protocol of PLA by detailed explanation in Materials and Methods, Immunocytochemistry, page 20-21 in the revised version. We performed single recognition assay using Duolink II Fluorescence kit (OLINK) with one primary antibody corresponding to each protein (rabbit anti-Smad2, rabbit anti-Smad3, rabbit anti-phospho-Smad2, rabbit anti-phospho-Smad3, and rabbit anti-Smad4) and the secondary anti-rabbit antibodies conjugated with oligonucleotides (PLA probe anti-rabbit PLUS and PLA probe anti-rabbit MINUS) to clearly detect and quantify the low level-endogenous proteins in primary lymph node cells freshly isolated from the melanoma-bearing mice. We used two different primary antibodies raised from different species against each protein, the secondary anti-rabbit antibody and the secondary anti-mouse antibody conjugated with oligonucleotides (PLA probe anti-rabbit PLUS and PLA probe anti-mouse MINUS) to detect the interaction of two proteins (rabbit anti-Smad2 and mouse anti-ubiquitin, rabbit anti-Smad3 and mouse anti-ubiquitin, rabbit anti-Smad4 and mouse anti-ubiquitin, mouse anti-Smad2/3 and rabbit anti-Smad4). CD8 was stained with conventional staining method using primary anti-CD8 antibody and secondary Alexa Fluor 488 conjugated anti-rat IgG.

Differential PSmad2 PLA data (and no effect on total level of Smad2 and Smad3) need to be consolidated with Western blot analysis.

Western blot analysis confirmed the PLA data showing the inhibitory effect of EW-7197 on phosphorylation of Smad2/3 without affecting total level of Smad2/3 in lymph nodes or in $CD8^+$ T cells in melanoma-bearing mice (Fig 2G in the revised version, 2 independently pooled samples/group).

In Figure 3a: When positive signals are obtained with Smad4 antibody and ubiquitin antibody, this does not (necessarily) mean that Smad4 is ubiquitinated. PLA only demonstrates that the proteins are in close proximity. Is there a PLA signal for Smad2 and ubiquitin?

PLA did not detect the signals (close proximity <40 nm) for interaction between Smad2 and ubiquitin or interaction between Smad3 and ubiquitin (Supporting Information Fig S7 in the revised version).

2. The ALK5 inhibition induced ubiquitination of Smad4 as shown in Fig. 3B data do not look convincing. Smad4 ubiquitination ladder cannot be seen. Most of the Ub-Smad4 have a lower molecular weight than Smad4?

It is unclear how this assay was performed. It needs to be done under denaturing conditions: otherwise no distinction can be made between Smad4 proteins that have been covalently modified by ubiquitin and protents that interact with Smad4 (non-covalently) that are ubiquitinated. This assay needs to be repeated and performed in a different manner.

We clarified the protocol to detect low-level endogenous ubiquitinated Smad4 in the limited amount of samples from the draining lymph node $CD8^+$ cells of melanoma-bearing mice using an UbiQapture-Q kit (Enzo Life Sciences) (Materials and Methods, page 22 in the revised version). We repeated the experiment to clearly show the Ub-Smad4 with higher molecular weight than Smad4 (Fig 3B in the revised version). Because only the limited number of $CD8^+$ cells could be obtained from dLNs (<10^6 $CD8^+$ cells/mouse, 5-7 mice were pooled for one sample), we could not perform other assays. The UbiQapture kit relies on a proprietary peptide that mimics a UBA (ubiquitin associating) domain from another protein bound to the resin, according to the manufacturer. In theory, it is possible that the UbiQapture matrix might pull down also proteins that interact with a ubiquitinated proteins bound to it, as the referee pointed out, but if these co-pulled down proteins are not covalently ubiquitinated, they will not show up in the WB analysis.

To address the referee's comment, we performed immunoprecipitation with anti-Smad4 antibody to detect ubiquitinated Smad4 under denaturing condition using primary mouse $CD8^+$ cells (2×10^7 $CD8^+$ cells/sample) stimulated in vitro in the presence or absence of EW-7197 or MG132 (Fig 3D, Materials and Methods in page 22-23 in the revised version). We dissociated non-covalent protein interactions with 1% SDS and boiling for 10 min for this assay as following the referee's comment.

3. The mechanism for selective Smad4 degradation in CD8 cells is unclear. The authors could investigate the possible involvement of Smurf.

In addition to the discussion of possible involvement of Smurf and other E3 ligases in page 12 in the first version, we performed the experiments to knockdown Smurf1 and/or Smurf2 by shRNA in $CD8^+$ cells as following the referee's suggestion. We found that knockdown of Smurf1/2 alone or in combination did not affect down-regulation of Smad4 protein by EW-7197 (Supporting Information Fig S8 in the revised version). We confirmed Smurf1/2 knockdown by quantitative RT-PCR. Western blot with the antibodies against Smurf1 or Smurf2 (Santa Cruz) failed to detect the endogenous proteins in mouse primary $CD8^+$ T cells (data not shown). Irrelevance of Smurf1/2 in ALK5 inhibition-induced ubiquitin-mediated degradation of Smad4 was described in page 8 in the result section and in page 14 in the discussion section in the revised version.

4. Is Eome protein differentially expressed in CD8 cells in vivo upon Smad4 deletion in CD8+ T cells or upon ALK5 inhibition?

In addition to the data showing the normal T cell homeostasis in vivo upon T cell-specific Smad4 deletion or upon ALK5 inhibition in SPF environment in Fig 4 in the first version, we confirmed the low expression of Eomes protein in $CD8^+$ T cells as well as normal homeostasis of immune cells in 16 week-old *Cd4Cre;Smad4*$^{+/+}$, *Cd4Cre;Smad4*$^{+/fl}$, and *Cd4Cre;Smad4*$^{fl/fl}$ mice or in C57BL/6 mice treated with gastric juice or EW-7197 for 8 weeks in the absence of melanoma challenge (Fig 7 and in page 10-11 in the revised version).

Referee 2.

We deeply appreciate the reviewer's instructive comments. We performed all the suggested experiments, which significantly improved our manuscript.

1) A more concrete demonstration that the effects of the inhibitors on restricting tumor growth are fully attributable to their effects on the CD8 T-cell compartment are needed. Minimally, immunocompromised (RAG-KO, Nude etc) mice bearing tumors could be treated showing no change in tumor growth. An ideal experiment might be to antibody deplete CD8, CD4, NK1.1 cells and show in each case how much of the efficacy of the inhibitors is lost.

In addition to the discussion on $CD8^+$ T-cell compartment as the main target of TGF-β antagonism with the previous reports in the references (Donkor et al, 2011; Gorelik et al, 2001; Nam et al, 2008; Zhang et al, 2005), we performed the primarily recommended experiments to delete $CD8^+$, $CD4^+$ or NK cells by anti-CD8, anti-CD4, or anti-asialo GM1 antibody, respectively (deletion of specific cell compartment was confirmed in Fig 6F and Supporting Information Fig 13). As shown in Fig 6A, 6B, and 6E and page 10 in the revised version, we confirmed that the anti-melanoma effect of the ALK5 inhibitor was completely abolished by deletion of $CD8^+$ cells. EW-7197 showed significant anti-tumor efficacy in $CD4^+$-deleted or NK-deleted mice (Fig 6C and 6D). Effect of EW-7197 on tumor growth and $CD8^+$ T cell expansion was slightly reduced in NK-deleted mice (Fig. 6D and 6F). The efficacy of EW-7197 following each antibody treatment was calculated as a % of the maximum therapeutic effect observed in the group treated with control IgG (Fig 6E). These data are consistent with the previous report by Nam et al, 2008, showing that the efficacy of neutralizing anti-TGF-β antibody on a 4T1 mammary tumor model mainly depends on $CD8^+$ T cells.

2) The authors need to discriminate between increases in cytotoxic or t-box transcription factors at the gene expression level in the overall node vs. on a per cell basis. In a few cases they insinuate these have increased "in CD8 cells in the dLN" when they have shown that the numbers of CD8 cells in the node have been increased substantially - thus we don't know if the higher Granzyme RNA (for example) is due to more CD8 cells, a higher per cell expression of Granzyme, or both. The flow

© EMBO

plots generally following the RNA analysis are helpful and show changes on a per cell basis. In the future, the authors may wish to sort pure populations of CD8 cells from the dLN for gene expression analysis which would allow them to discriminate between changes in per cell expression level vs. changes in the density of CD8 cells in the overall LN.

We confirmed that increases in Eomes and cytolytic molecules at the gene expression level were both in the overall node and on a per cell basis. We sorted out $CD8^+$ cells from the dLNs to repeat quantitative RT-PCR (Fig 1E, 4E, and 5C). Gene expression patterns in sorted $CD8^+$ dLN cells were similar to those in the whole dLNs with 5- to 10-fold increases in the expression levels due to the enrichment of $CD8^+$ cells (Supporting Information Fig 3D, 3E, 9C, and 10D). Because of the limited space, we show the overlay histograms in Fig 5A and show the dot plots in Supporting Information Fig S10A and B. These results obtained from quantitative RT-PCR and dot plots show that both mRNA and protein levels of Eomes and cytolytic molecules in $CD8^+$ cells were increased on a per cell basis. Thus, increase in Eomes and cytolytic molecules at the mRNA expression level in the whole dLNs was due to the changes in both per cell expression level and the density of $CD8^+$ cells (Fig 1C and 4C).

We hope the authors in the future will also consider analyzing TIL instead of dLN cells as the results can often be quite distinct.

We investigated TILs as following the referee's advice. TILs were remarkably increased in EW7197-treated or *Cd4Cre;Smad4*$^{fl/fl}$ mice, which were barely observed in the vehicle-treated control or wild type control *Cd4Cre;Smad4*$^{+/+}$ mice (Supporting Information Fig S3F and S9D in the revised version, more than 1,000,000 events/sample acquired). Consistent with the immunohistochemistry detecting the melanoma-infiltrating $CD8^+$ T cells only in EW7197-treated or *Cd4Cre;Smad4*$^{fl/fl}$ mice (Fig1H, 4H, and 5D in the first version, Fig 1I, 4I, and 5E in the revised version), $CD8^+$ T cells were not detected in TILs isolated from the vehicle-treated control or wild type control *Cd4Cre;Smad4*$^{+/+}$ mice, whereas significant $CD8^+$ T cells expressing high levels of Eomes were present in TILs isolated from EW7197-treated or *Cd4Cre;Smad4*$^{fl/fl}$ mice (Fig 1H, 4H, and 5D). Proportions of the immune cell subsets in TILs showed no significant differences among the groups (Supporting Information Fig S12), although absolute numbers of TILs were different.

3) The authors assert that degradation of Smad4 leads to de-repression of Eomesodermin; however, Ichiyama and Yoshimura (2011) published that TGF-b mediated repression of Eomes was through a JNK driven, Smad-independent (at least Smad2 and 3 independent) pathway. The authors need to more thoroughly describe how their data integrates with the published pathway.

We added more detailed discussion on the report by Ichiyama and Yoshimura (2011) in page 15 in the revised version: "By contrast, it has been reported that TGF-β suppresses Eomes via Smad2/3-independent, JNK-dependent signalling in Th17 induction (Takimoto et al, 2010; Ichiyama et al, 2011). Discrepancy between their reports and our study might be due to several reasons: TGF-β signalling pathways to suppress Eomes might be different between $CD4^+$ and $CD8^+$ T cell effector subsets, Smad4 was not investigated in their reports, they used T cells from *LckCreSmad2*$^{fl/fl}$*Smad3*$^{-/-}$ (Smad2/3-DKO) or *LckCreSmad2*$^{fl/fl}$*Smad3*$^{+/-}$ (Smad2cKO/Smad3hetero) mice, so that Smad4 alone or Smad4 and haploid expression of Smad3 could still transduce TGF-β signalling to repress the *Eome*s gene according to our findings (Fig 9A and B). They speculated JNK-dependent, Smad2/3-independent pathway from the similar attenuating effect of ALK5 inhibitor, SB431542 and JNK inhibitor, SP600125 on Eomes repression in T cells stimulated with TCR and TGF-β. However, specificity of ALK5 inhibitors for Smad-mediated TGF-β signalling pathway (Akhurst & Hata, 2012; Flavell et al, 2010; Hawinkels et al, 2011; Jin et al, 2011) and cooperation of Smad3 and Smad4 with c-Jun/c-Fos to mediate TGF-β-induced transcription (Zhang et al, 1998) suggest that both Smad3/4 and JNK pathways are involved in TGF-β-induced Eomes suppression."

If TGF-β ligand antagonists, such as neutralizing anti-TGF-β antibodies and soluble TGF-β II receptor, which block all the intracellular TGF-β signalling pathways showed the same attenuating effect with JNK inhibitor on Eomes suppression, whereas ALK5 inhibitors, which specifically block Smad pathway failed to show such an attenuating effect, one could conclude that TGF-β suppresses Eomes via JNK-dependent, Smad-independent signalling. The report by Zhang et al is the important reference to show the mergence of Smad pathway and the downstream of JNK.

4) Lack of induction of IFN-g in concert with Eomes induction is unusual given that Eomes (+Runx3) drives IFN-g expression more so than it does Granzyme B. I believe the lack of IFN-g induction seen by the authors in Figure 6 and elsewhere may be due to the activation of the T-cells in vitro using aCD3/aCD28 beads. These beads are the best reagents for measuring proliferative changes; however, PMA/Ionomycin stimulation is generally superior for studying in vitro IFN-g production.

We confirmed that expression patterns of IFN-γ (mRNA and protein) in *Smad4*$^{-/-}$ or *Smad4*$^{+/+}$ CD8^{+} T cells stimulated with PMA and ionomycin for 6, 24, and 48 h (Fig S15 and page 11 in the revised version) were similar to those in CD8^{+} T cells stimulated with anti-CD3/CD28 antibodies for 72 h in vitro (Fig 7A, B, D, and E in the first version, Fig 8 A, B, D, and E in the revised version), and those in CD8^{+} T cells from the draining lymph nodes of melanoma-bearing mice, which were re-stimulated with PMA and ionomycin for 1-4 h ex vivo (Fig 6A-C in the first version, Fig 5A-C in the revised version). We did not show the data of CD8^{+} T cells stimulated with PMA and ionomycin for 72 h, because most of the cells were dead.

As the referee suggested that Runx3 cooperates with Eomes in transcription of IFN-γ and the cytolytic molecules, Runx3 also cooperates with Smad3/4 to regulate human germ-line IgA genes (Pardali et al, 2000; Zhang et al, 2000). We discussed that the possible mechanism of the discrepancy in the expression of Eomes and IFN-γ in CD8^{+} T cells might be that the cooperation of Runx3 with Smad4 is required for Eomes to induce IFN-γ, but not to induce the cytolytic molecules (Discussion, page 15 in the revised version).

Minor Specifics:

In the abstract the sentence starting with "Notably, progression....melanoma-bearing mice" is a run-on and unclear and should be re-worked.

We have amended the text in the abstract by dividing the sentence into two sentences (the second and fifth sentences in the revised abstract).

Calling Eomes "the essential T-box transcription factor for CTL functions" in the abstract is overstated given that Intelkofer and Reiner showed that Eomes-/- CTL are fully functional.

We deleted the word "essential" and corrected the description "the T-box transcription factor regulating CTL functions" in the abstract.

In the introduction "heteromeric" is a not word. Perhaps heterodimeric?

R-Smad-Smad4 complexes can be heterodimers or heterotrimers. Please refer to the references such as Massague et al, 2005, Brown et al, 2007, Hawinkels et al, 2011, which use the word "heteromeric" to describe R-Smad-Smad4 complexes.

It would be nice to hear at least a speculative explanation for why Alk5 inhibition leads to Smad4 down-regulation in CD8 T-cell but not CD4 T-cells or B16 tumor cells as this mechanism is critical to the postulated effect of the inhibitors.

Among the candidate E3 ligases to induce ubiquitin-mediated degradation of Smad4 in CD8^{+} T cells, which we discussed in page 12 in the first version, it has been reported that IL-7 modulates TGF-β signalling via Smurf2 activity in CD8^{+} T cells (Pellegrini et al, 2009). At the referee 1's suggestion, we investigated the possible involvement of Smurf1/2. However, knockdown of Smurf1 and/or Smurf2 by shRNA did not affect EW-7197-induced Smad4 down-regulation in CD8^{+} T cells (Supporting Information Fig S8 and page 7 in the revised version). Thus far, too little is known about ubiquitination of Smads in immune cells to speculate as to the possible mechanisms of our finding more than the discussions in page 14 in the revised version.

2nd Editorial Decision 31 July 2013

Thank you for the submission of your revised manuscript to EMBO Molecular Medicine. We have now heard back from the two Reviewers whom we asked to re-evaluate your revised manuscript.

You will see that while Reviewer 2 is now satisfied, Reviewer 1 points to remaining issues that prevent us from considering publication at this time.

Specifically, Reviewer 1 has some important remaining concerns on the PLA and ubiquitination experiments. S/he disagrees on the interpretation of the PLA signal and the quality of controls for these experiments. Furthermore, this Reviewer is concerned that only very little if any effect of EW-7197 is observed on the subcellular distribution of Smads and that the effect of EW-7197 on ubiquitination of Smad4 does not appear to be specific. Reviewer 1 also notes that dose-dependency is not apparent from Figure 3D and would like to understand why the proteasome inhibitor does not substantially potentiate the Smad4 smear. This Reviewer also lists other very important experimental shortcomings and requests for clarification that require your action.

Although we would normally not allow a second revision, I am prepared in this case, to give you another opportunity to improve your manuscript, with the understanding that the Reviewer's concerns must be fully addressed with additional experimental data where appropriate and that next version of the manuscript will undergo a third and final round of review with the Reviewer.

As you know, EMBO Molecular Medicine has a "scooping protection" policy, whereby similar findings that are published by others during review or revision are not a criterion for rejection. However, I do ask you to get in touch with us after three months if you have not completed your revision, to update us on the status. Please also contact us as soon as possible if similar work is published elsewhere

I look forward to seeing a revised form of your manuscript as soon as possible.

***** Reviewer's comments *****

Referee #1 (Remarks):

The authors have improved their manuscript. However, I am not convinced by the the PLA and ubiquitination experiments and the conclusions that are drawn from them.

Specific comments:
1. It is still unclear whether single primary antibody incubations were performed as negative controls. When only one primary antibody is taken along this should not give significant PLA signals.
2. A PLA signal is interpreted by authors as interaction. This is incorrect. A positive signal in PLA with antibodies against two proteins only tells us that the proteins are in close proximity.
3. Figure 2. TGF-beta-induced Smad2/3 phosphorylation and Smad2, Smad3 and Smad4 heteromeric complex formation and nuclear accumulation is inhibited by TGF-beta receptor kinase inhibitors. Why do the authors observe only very little to no effect of EW-7197 on the subcellular distribution of Smads?
4. In Figure 3B and D EW-7197 induces a more intense smear of poly-ubiquitinated Smad4 in CD8+ cells. The same assay should be repeated with CD4+ cells in which the Smad4 protein downregulation is not observed.
5. Figure 3B, lower panel. This figure shows that upon Ew-7197 treatment also the total number of proteins that get ubiquitinated increases. Thus the effect of EW-7197 on ubiquitination of Smad4 is not specific. The authors need to comment upon this.
6. Figure 3C. CD4+ cells are not indicated in this figure.
7. Figure 3F. The tumor cells do respond to TGF-beta as shown by Smad2 phosphorylation in the left panel. However, how do the authors explain why there is no effect on the subcellular distribution of Smad4 upon TGF-beta or EW-7197 treatment? An expected result would have been that upon TGF-beta treatment more Smad4 PLA spots are detected in the nucleus, and the reverse

upon EW-7197 treatment.
8. Figure 3D. The dose dependent is not apparent from this figure: there is no difference in Smad4 down regulation with 2.0 versus the 5.0 dose. It is unclear why proteasome inhibitor does not (greatly) potentiate the detection of a Smad4 smear.
9. 9. Figure 3E. Does EW-7197 have an effect on Smad4 subcellular distribution?

Referee #2 (Remarks):

I'm very satisfied with the revisions performed by the authors. The quality and interpretability of the manuscript has improved significantly and I consider it appropriate for publication.

2nd Revision - authors' response 20 August 2013

Response to Referee 1

We have done our best to respond to the referee's important remaining concerns on the first revised version. We especially thank the referee for the important advices on the experiments to detect ubiquitination of Smad4. We have included the comments by the referee in bold, which are followed by our response in the order of the contents of the concerns raised by the referee: PLA (1, 2), ubiquitination experiments (4, 5, 8), subcellular distributions of Smads (3, 7, 9), and others (6). Corrections in the second revised version are underlined and marked with red.

<PLA: comment 1, 2>

1. It is still unclear whether single primary antibody incubations were performed as negative controls. When only one primary antibody is taken along this should not give significant PLA signals.

As the referee pointed out, we had confirmed no background signals when one of the primary antibodies was omitted in double recognitions (rabbit anti-Smad2, rabbit anti-Smad3, rabbit anti-Smad4, mouse anti-ubiquitin, mouse anti-Smad2/3, respectively, in combination with PLA probe anti-rabbit PLUS, PLA probe anti-mouse MINUS and rat anti-CD8 antibody). Please refer to the explanation in line 8-9, page 22 and the images in the attached original data.

2. A PLA signal is interpreted by authors as interaction. This is incorrect. A positive signal in PLA with antibodies against two proteins only tells us that the proteins are in close proximity.

We have corrected the descriptions of all the corresponding parts, in which we used to follow the descriptions by the manufacturer and the published papers in the previous versions (http://www.olink.com/products/duolink/applications/protein-interactions, http://www.olink.com/products/duolink/publications). Accordingly, we added the explanation in line 15-17, page 7: "To confirm whether Smad4 in close proximity with ubiquitin by the treatment with EW-7197 is ubiquitinated, endogenous ubiquitinated Smad4 was captured by UbiQapture matrices". We corrected the descriptions by replacing "interactions" with "close proximity". Please refer to the corrected parts: line 17-18 and line 22 in page 6, line 11, 14, and 15-19 in page 7 in Results section, line 3, page 22 in Materials and Methods section, Figure 2A-F, Figure 3A in Figure legends in the second revised version.

<Ubiquitination experiments: 4, 5, 8>
4. In Figure 3B and D EW-7197 induces a more intense smear of poly-ubiquitinated Smad4 in CD8+ cells. The same assay should be repeated with CD4+ cells in which the Smad4 protein down-regulation is not observed.

We repeated the experiments using CD8- and CD8+ dLN cells for Figure 3B and the experiments using CD4+ and CD8+ cells for Figure 3D. We used two independent samples for each group of EW-7197 treatment (0 and 2.5 mg/kg) for better verification (Figure 3B). As shown, we confirmed that the treatment with EW-7197 induced more intense smears of ubiquitinated Smad4 in CD8+ cells than those in CD8- cells (Figure 3B) and CD4+ cells (Figure 3D). Accordingly, we added the descriptions in Result sections in line 17-18, line 24-25, page 7, and Figure legend 3D: "Ubiquitination of Smad4 was enhanced significantly in $CD8^+$ dLN cells by the treatment with EW-7197, whereas it was not altered in $CD8^-$ dLN cells (Fig 3B)", "EW-7197 induced ubiquitination of Smad4 accompanied with protein downregulation in activated $CD8^+$ T cells, but not $CD4^+$ T cells in a dose dependent manner (Fig 3D)", and "IP-Western blot shows endogenous ubiquitinated Smad4 in $CD4^+$ and $CD8^+$ cells stimulated with anti-CD3/CD28 with/without EW-7197 and/or MG-132 for 3 days".

5. Figure 3B, lower panel. This figure shows that upon Ew-7197 treatment also the total number of proteins that get ubiquitinated increases. Thus the effect of EW-7197 on ubiquitination of Smad4 is not specific. The authors need to comment upon this.

We repeated the experiments to blot with anti-Ub antibody. We used two independent samples for each group of EW-7197 treatment (0 and 2.5 mg/kg) as explained above and confirmed no significant differences in the total ubiquitinated proteins among the samples. We paid close attention to the lysate concentration (8×10^6 cells from 10 mice/sample, in page 23, Western blotting and in vivo ubiquitination assay in Materials and Methods section, 5×10^6 cells from 5-7 mice/sample were used in the previous version) the exposure time for the repeated experiments.

8. Figure 3D. The dose dependent is not apparent from this figure: there is no difference in Smad4 down regulation with 2.0 versus the 5.0 dose. It is unclear why proteasome inhibitor does not (greatly) potentiate the detection of a Smad4 smear.

It was presumably due to the relatively small numbers of the primary T cells used for immunoprecipitation in the first revised version (10^7 cells in 1 ml of IP buffer/sample). We repeated the IP experiments with more T cells (5×10^7 cells in 1 ml of IP buffer/sample) to provide the clearer results with a more intense Smad4 smear upon MG132 treatment (upper panel). Repeated Western blots with anti-Smad4 antibody showed the dose-dependent Smad4 down-regulation (middle panel). Please also refer to the original gels attached to the first revised version showing the dose-dependency between 2.0 and 5.0 μM.

<Subcellular distributions of Smads: 3, 7, 9>

We added the more detailed descriptions about the subcellular localization of Smads in page 8 and the discussion on the differences of subcellular distributions of Smads in lymph node cells and in B16 melanoma cells in page 14-15 in the second revised version.

3. Figure 2. TGF-beta-induced Smad2/3 phosphorylation and Smad2, Smad3 and Smad4 heteromeric complex formation and nuclear accumulation is inhibited by TGF-beta receptor kinase inhibitors. Why do the authors observe only very little to no effect of EW-7197 on the subcellular distribution of Smads?

This comment is on <u>the subcellular distribution of Smad2 and Smad3 in the draining lymph node cells of melanoma-bearing mice.</u>

As the referee pointed out, most of C-terminally unphosphorylated Smad2 and Smad3 proteins were still located in the nuclei of draining lymph node cells when Smad4 was down-regulated by the oral treatment with EW-7197. Lymphocytes are activated with intensive stimuli through T/B cell receptors in combination with co-stimulatory molecules, and/or cytokine receptors, which activate the signalling pathways through serine/threonine kinases, such as MAPKs and PKC. Although these kinases phosphorylate the linker regions or MH1 domains of R-Smads, very little is known about the signalling networks between these serine/threonine kinases and Smads in lymphocytes. We added the discussion on the nuclear localization of C-terminally unphosphorylated R-Smads in the

draining lymph node cells of melanoma-bearing mice treated with EW-7197 in page 14-15 with the references (Chang et al, 2011; Heldin et al, 2012; Matsuzaki, 2013).

7. Figure 3F. The tumor cells do respond to TGF-beta as shown by Smad2 phosphorylation in the left panel. However, how do the authors explain why there is no effect on the subcellular distribution of Smad4 upon TGF-beta or EW-7197 treatment? An expected result would have been that upon TGF-beta treatment more Smad4 PLA spots are detected in the nucleus, and the reverse upon EW-7197 treatment.

This comment is on <u>the subcellular distribution of Smad4 in B16 cells in culture.</u>

We lowered the intensity of green fluorescence in B16 cells transfected with GFP to show the clearer subcellular distribution of Smad4 red PLA dots. We also quantified the expression of Smad4 in the cytoplasms and nuclei using BlobFinder software, which proved the expected tendency: more Smad4 PLA dots in the nuclei upon TGF-β treatment (the expression ratio of nucleus to cytoplasm: 3.48) and more Smad4 PLA dots in the cytoplasms upon EW-7197 treatment (the expression ratio of nucleus to cytoplasm: 0.36). Please refer to Figure legend 3F.

9. Figure 3E. Does EW-7197 have an effect on Smad4 subcellular distribution?

This comment is on <u>the subcellular distribution of Smad4 in B16 melanomas in vivo.</u>

We quantified the expression of Smad4 protein in the cytoplasms and nuclei detected by HRP/DAB method using ImageJ software (explained in Histology, Materials and Methods in page 21). EW-7197 inhibited the nuclear translocation of Smad4 in B16 melanomas as shown in the images and the graph in Figure 3E (the expression ratio of nucleus to cytoplasm: 0.32) in the second revised version. Please refer to Figure legend 3E.

<Others>

6. Figure 3C. CD4+ cells are not indicated in this figure.

Please see the upper blots labelled "$CD4^+$ cells".

3rd Editorial Decision 23 August 2013

Thank you for the submission of your revised manuscript to EMBO Molecular Medicine. We have now received the enclosed report from the referee who was asked to re-assess it. As you will see this reviewer is now supportive and I am pleased to inform you that we will be able to accept your manuscript pending the following final amendments:

-Please comment on the referee's concern, include this in the main text of the manuscript, and correct Figure 3B legend.

- Please indicate in Fig. 2G that the data presented does not come from a single blot but is assembled on figure by adding a vertical black bar crossing the blot.

-It appears that Supporting Information Figure 14 is also an assembled figure made of several panels put together, please visually clarify and ideally, provide the original gels.

Please submit your revised manuscript within two weeks. I look forward to seeing a revised form of your manuscript as soon as possible.

***** Reviewer's comments *****

Referee #1 (Remarks):

The authors have improved the manuscript,and answered most of my concerns.

Comments on Fig. 3B:
Upper panel: Smad4 is indicated on the left side; is this Smad4 modified by ubiquitin (non ubiquitinated Smad4 monomer should not be detected with the procedure that is used)? Is this band due to non-specific sticking of Smad4 or is this non-modified Smad4 pulled down when in complex with ubiquitinated Smad4 and gets separated and detected upon Western blot analysis?

Lower panel Fig. 3B is not explained in Fig. 3B legend.

3rd Revision - authors' response 25 August 2013

Response to Referee 1

We deeply appreciate all the precious comments and instructions by the referee during the whole process of review. We have included the comments by the referee in bold, which are followed by our response. We thank the referee again for the precious time and consideration.

Comments on Fig. 3B:
Upper panel: Smad4 is indicated on the left side; is this Smad4 modified by ubiquitin (non ubiquitinated Smad4 monomer should not be detected with the procedure that is used)? Is this band due to non-specific sticking of Smad4 or is this non-modified Smad4 pulled down when in complex with ubiquitinated Smad4 and gets separated and detected upon Western blot analysis?

As the referee pointed out, this method is not supposed to detect non-ubiquitinated proteins. We had meant to indicate the molecular weight of Smad4 (70 kD), but not non-ubiquitinated Smad4 in the previous version.

We have deleted the indication of Smad4 (pointing a bit lower than the lowest band in the previous version) in the figure to avoid misunderstandings. Instead, we have added the information of molecular weight of Smad4 in the figure legend.

Lower panel Fig. 3B is not explained in Fig. 3B legend.

We thank the referee for careful reading and instructions. Explanation for the lower panel has been included in the figure legend.
