# Supplementary material for: Activin receptor-like kinase5 inhibition suppresses mouse melanoma by ubiquitin degradation of Smad4, thereby derepressing eomesodermin in cytotoxic T lymphocytes
Source: EMBO Mol Med. 2013 Oct 11;5(11):1720–39. doi: 10.1002/emmm.201302524 (PMC3840488; doi:10.1002/emmm.201302524)
Supplement: Supplementary file 2 [file emmm0005-1720-SD2.pdf]

Supporting Information

Supporting Materials and Methods -- Page 2

Supporting Materials and Methods -- Page 3

Supporting Information Table I. -- Page 4

Supporting Information Table II. --- Page 5

Supporting Information Table III. -- Page 6

Supporting Information Figure 1. -- Page 7

Supporting Information Figure 2. -- Page 8

Supporting Information Figure 3. -- Page 9

Supporting Information Figure 4. -- Page 10

Supporting Information Figure 5. -- Page 11

Supporting Information Figure 6. -- Page 12

Supporting Information Figure 7. -- Page 13

Supporting Information Figure 8. -- Page 14

Supporting Information Figure 9. -- Page 15

Supporting Information Figure 10. -- Page 16

Supporting Information Figure 11. --- Page 17

Supporting Information Figure 12. --- Page 18

Supporting Information Figure 13. --- Page 19

Supporting Information Figure 14. --- Page 20

Supporting Information Figure 15. --- Page 21

Detection of cell cycle and apoptosis of B16 cells

B16 cells were cultured in the absence or presence of TGF-β (0, 0.5, 1.0, 5.0 ng/ml) and/or EW-7197 (0, 0.25, 0.5, 1.0 μM). For cell cycle analysis, DNA content was determined by Propidium iodide (PI) (BD Pharmingen). Briefly, B16 cells were fixed by cold 80% ethanol overnight at -20°C. Fixed cells were washed twice in phosphate-buffered saline (PBS), 1% bovine serum albumin (BSA), and then resuspended in PI solution in PBS containing RNase A (0.1 mg/ml; Sigma-Aldrich). Phosphatidylserine exposure was measured using Annexin V Apoptosis Detection Kit APC (eBioscience). B16 cells were resuspended in 1× binding buffer (10^5 cells/100 μL) and incubated with Annexin V-APC for 15 minutes at room temperature in the dark. Cells were washed, resuspended in 1× binding buffer (200 μl). Propidium iodide (PI) staining solution was added into each sample and analyzed by flowcytometry immediately.

Knockdown of Smurf1 and Smurf2 by shRNA

The short hairpin RNA (shRNA) sequences specific for endogenous *Smurf1* and *Smurf2* are described previously (Lee YS et al, 2011). To generate shRNAs specific for endogenous *Smurf1* and *Smurf2*, double-stranded oligomers containing restriction enzyme sites, a sense sequence, and a loop sequence with its antisense sequence were designed and cloned into *Age*I and *Eco*RI sites of the pLKO-puro vector (Clontech). Lentiviruses expressing each shRNA were produced by a lentiviral packaging system from Invitrogen. Lentivirus expressing mutant *Gfp* shRNA was used as a negative control for lentivirus infection. Expression levels of *Smurf1* and *Smurf2* mRNA were measured by real-time quantitative PCR with the primers: Smurf1 5'-TGCCATCAGCAGATTGAAAG-3', 5'-GTTCCTTCGTTCTCCAGCAG-3', Smurf2 5'-GTGAAGAGCTCGGTCCTTTG-3', 5'-TCGCTTGTATCTTGGCACTG-3'

Table I. Primer sequences for quantitative RT-PCR

Gene	Sense primer	Antisense primer
Gapdh	TGGTGAAGGTCGGTGTGAAC	CCATGTAGTTGAGGTCAATGAAGG
T-bet	GCCAGGGAACCG CTTATATG	GACGATCATCTGGGTCACATTGT
Eomes	TGAATGAACCTTCCAAGACTCAGA	GGCTTGAGGCAAAGTGTTGACA
Ifng	ACTGGCAAAAGGATGGTGAC	GACCTGTGGGTTGTTGACCT
Gzmb	GGACTGCAAAGACTGGCTTC	ATAACATTCTCGGGGCACTG
Prf1	TTTCGCCTGGTACAAAAACC	AGGGCTGTAAGGACCGAGAT
Fasl	CATCACAACCACTCCCACTG	GTTCTGCCAGTTCCTTCTGC
Smad4	GCAGCTCTTGGATGAAGTCC	GGCAGCAAACACATCTCTCA

Table II. Primer sequences for the proximal promoter regions of Eomes

Eomes promoter	Sense primer	Antisense primer
-2.0 kb	TTTGGTACCCTGTGCCACGCCAGCGTTTCC	AAACTCGAGGCTTTAGCGAATCGCAGACGG
-0.7 kb	TTTGGTACCATGTTCGCAGACTTCAAACCC	AAACTCGAGGCTTTAGCGAATCGCAGACGG
-0.37 kb	TTTGGTACCTGTGAGTGTAGGGGTCCTGA	AAACTCGAGGCTTTAGCGAATCGCAGACGG
-0.23 kb	TTTGGTACCTTTCTTGCGGAAGGAAAGG	AAACTCGAGGCTTTAGCGAATCGCAGACGG

Table III. Primer sequences for ChIP

**Eomes promoter**	**Sense primer**	**Antisense primer**
-912 to -720	AGTCTCAACAATGGGGTCGT	CGTGTGAGTGTGCATGTCTG
-680 to -449	GGGTCGTAGAAACCCTAGAAATCA	GAGACAGGGTCTCCCATGGA
-538 to -321	ATCCTCCACAGACATGCACA	GTTCTTAGCCCCAGGGAGAC
-239 to -86	AGAGCACTGGGTGCTACTGGTT	CCCTTCGCTCCCAGCAT
-172 to +72	CTGCCCTCTCCACGCCAGGT	AAACAGCAGGGCAGGAGCCG

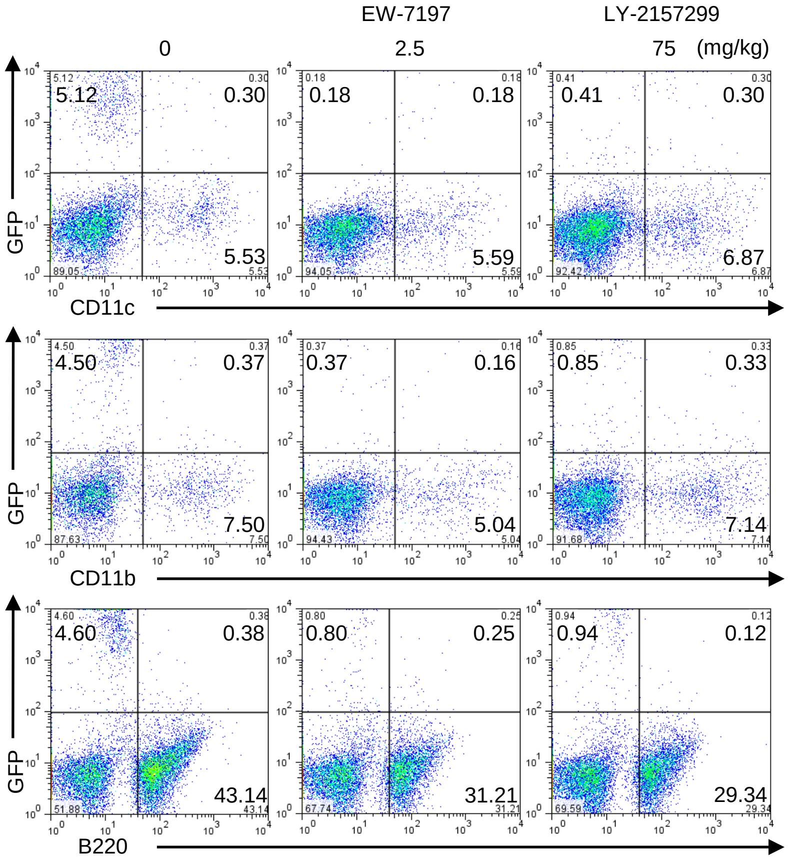

Supporting Information Figure 1. Detection of lymph node metastsis. Dot plots by FACS analyses show GFP⁺CD11c⁻, GFP⁺CD11b⁻, and GFP⁺B220⁻ cells in the dLNs of melanoma-bearing mice three weeks after inoculation.

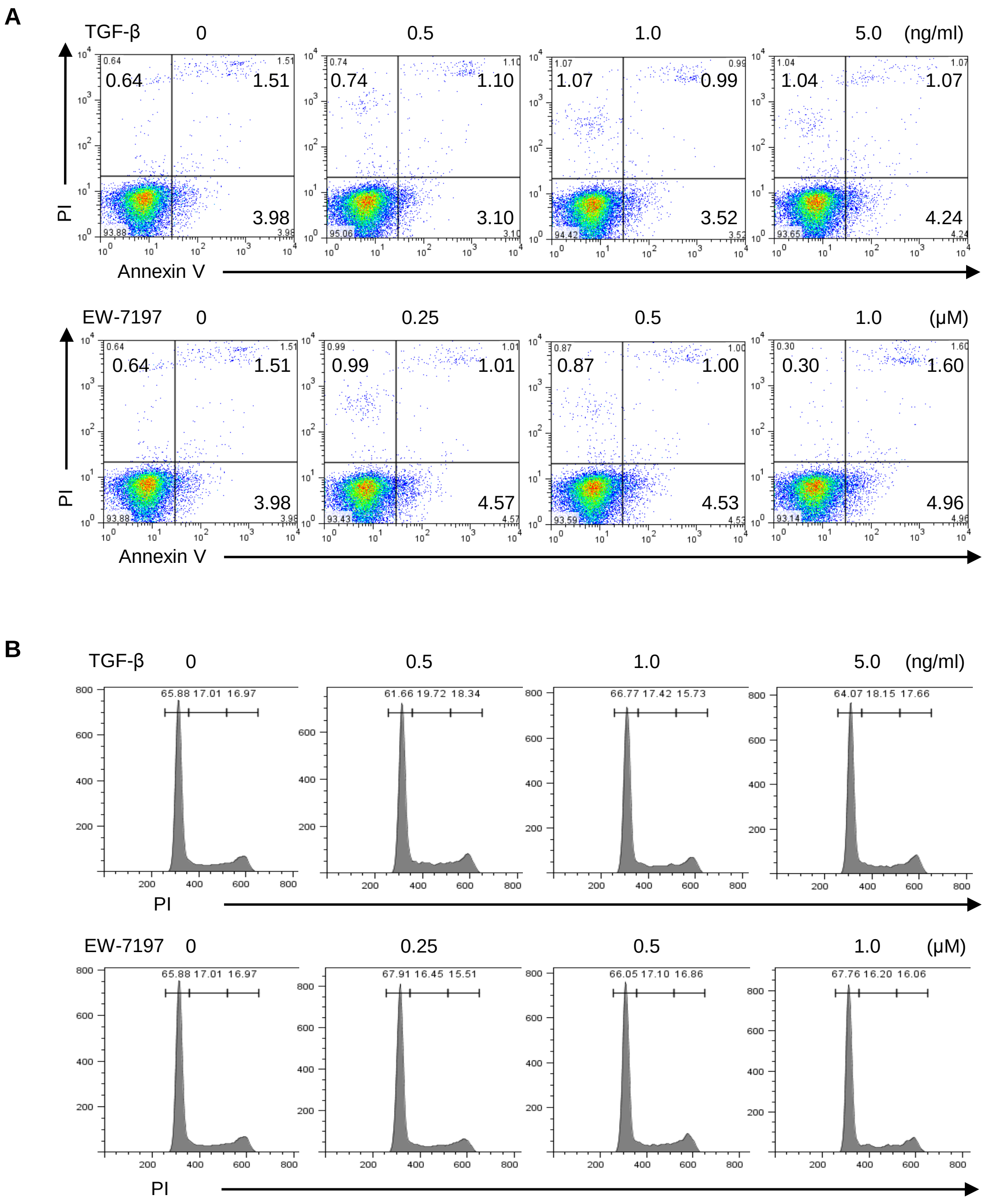

Supporting Information Figure 2. TGF-β does not affect apoptosis and cell cycle of B16 cells. B16 cells were treated with TGF-β (0, 0.5, 1.0, 5.0 ng/ml) or EW-7197 (0, 0.25, 0.5, 1.0 μM) for 72 h. **A.** Apoptosis was determined by Annexin V and PI staining. **B.** Cell cycle was determined by PI staining using flowcytometry.

Supporting Information Figure 3

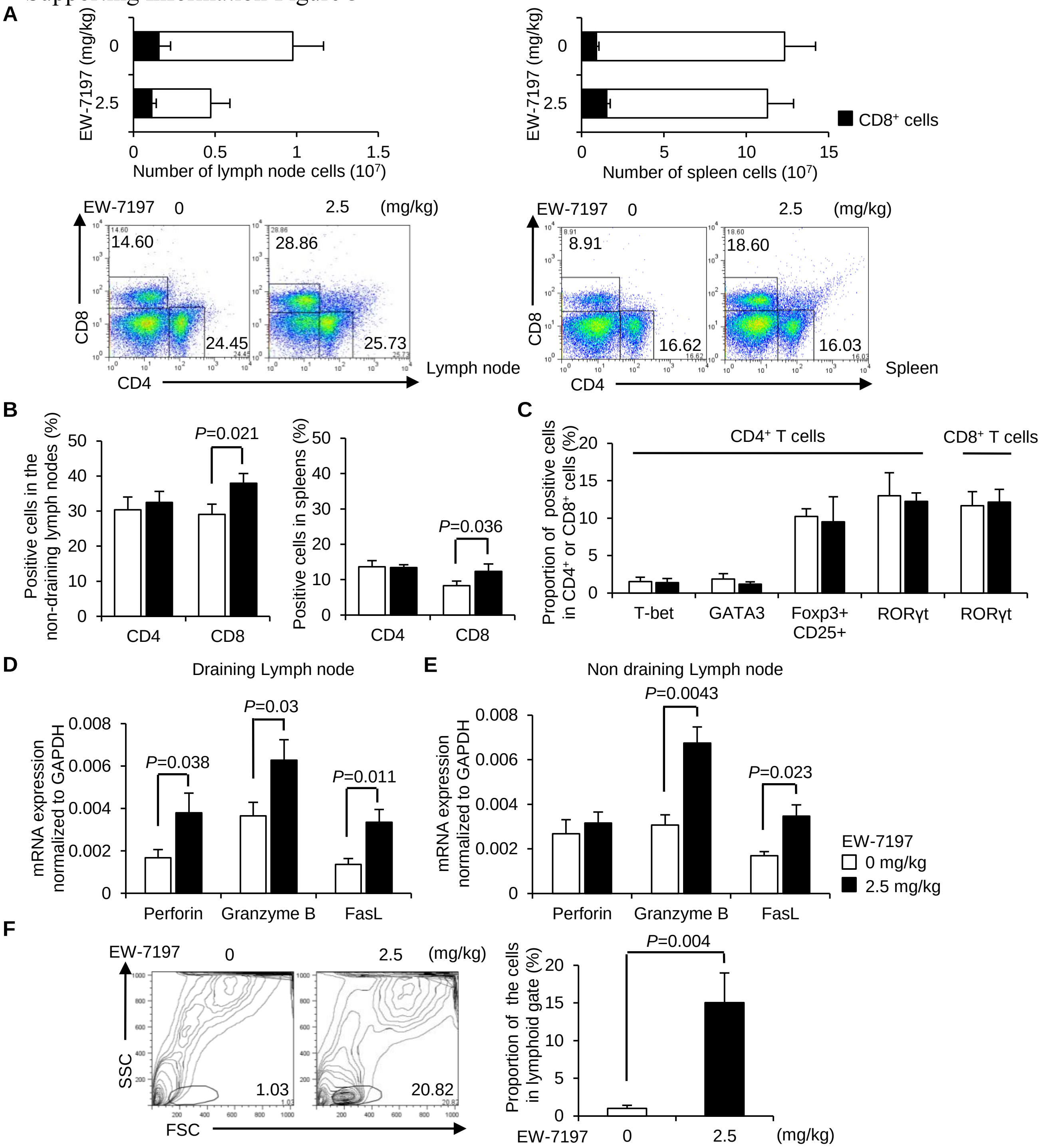

Supporting Information Figure 3. Oral administration of EW-7197 suppresses melanoma and LN metastases with enhanced CTL activity. Data are shown as mean + SEM (n = 5-10/group). P values were calculated by 2-tailed unpaired Student's t test. **A.** Total and CD8$^+$ cell numbers of LNs and spleens in the melanoma-bearing mice. Representative dot plots of CD4/8 are shown. **B.** Percentages of CD4/8 subsets in the non-dLNs and spleens. **C.** Percentages of effector T cell subsets in the dLNs. **D,E.** qPCR analyses for mRNA levels of the cytolytic molecules in the dLNs and the non-dLNs. **F.** Representative FSC/SSC contour plots of enriched tumor-infiltrated cells by density gradient centrifugation.

Supporting Information Figure 4

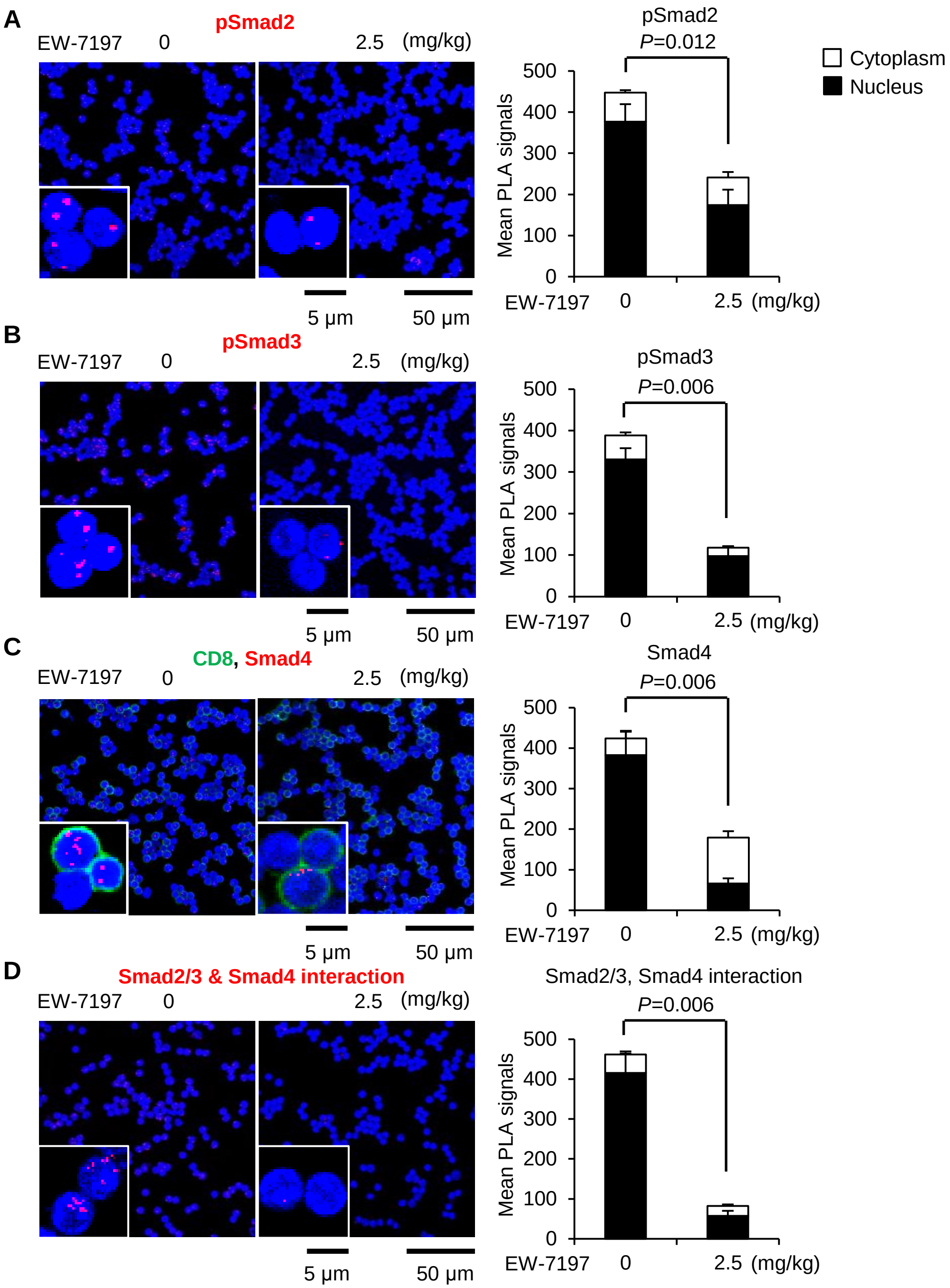

Supporting Information Figure 4. Oral administration of EW-7197 inhibits TGF-β signaling and downregulates Smad4 in spleen cells of melanoma-bearing mice. A-D. Expression of phospho-Smad2, phospho-Smad3, Smad4, and the interaction between Smad2/3 and Smad4 from EW-7197-treated mice was determined by PLA. Images were acquired by confocal microscope, LSM700 (scale bars: 5 μm, 50 μm). Graphs show the quantification of the red dots expressed in nucleus and cytoplasm. Data are shown as mean + SEM (n = 5/group). P values were calculated by 2-tailed unpaired Student's t test.

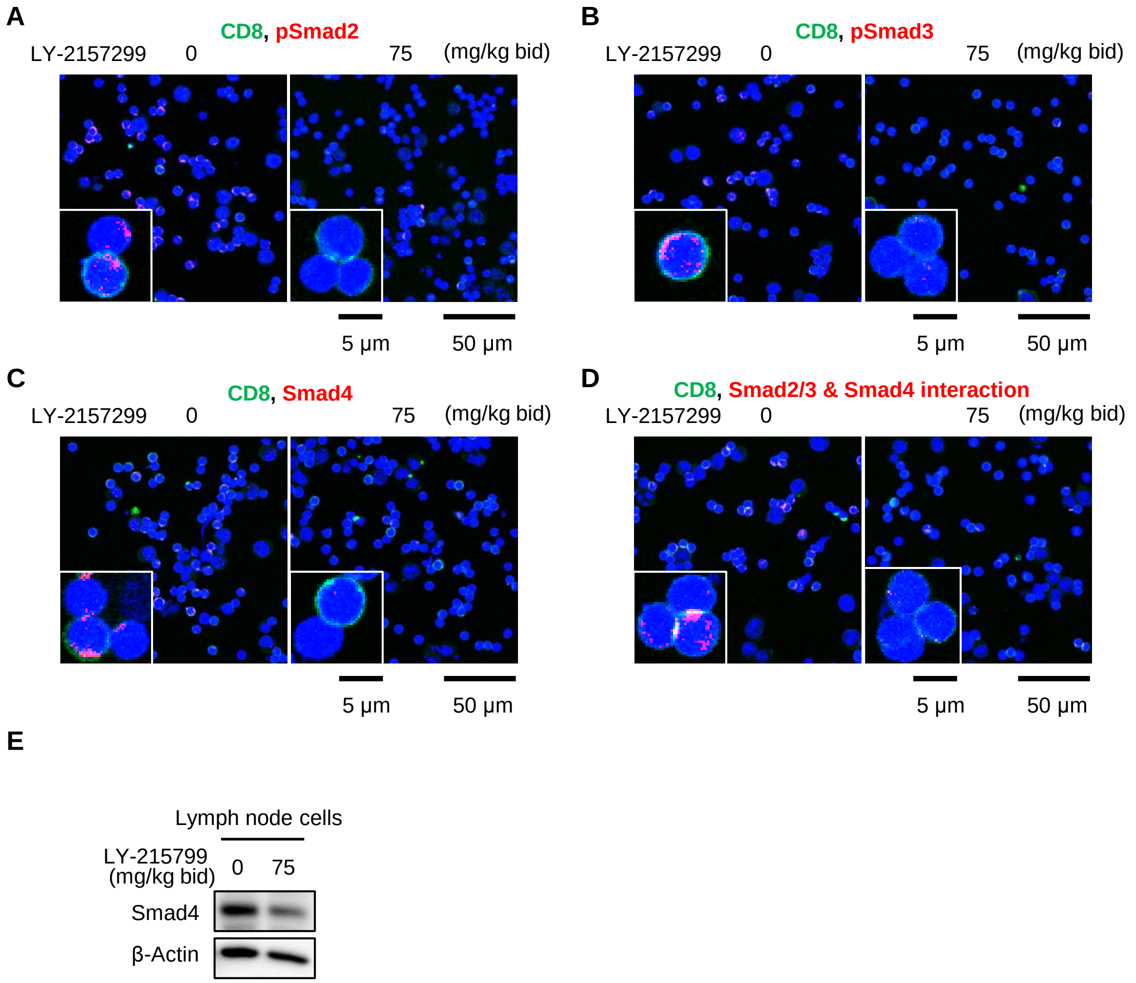

Supporting Information Figure 5. Oral administration of LY-2157299 inhibits TGF-β signaling and downregulates Smad4 in dLN cells of melanoma-bearing mice. **A-D.** Expression of phospho-Smad2, phospho-Smad3, Smad4, and the interaction between Smad2/3 and Smad4 in dLN cells from vehicle- or LY-2157299-treated mice was determined by PLA. Images were acquired by confocal microscope, LSM700 (scale bars: 5 µm, 50 µm). **E.** Smad4 and β-Actin in dLN cells from vehicle- or LY-2157299-treated mice were detected by Western blotting.

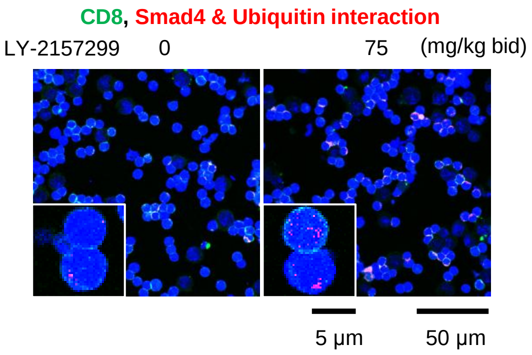

Supporting Information Figure 6. LY-2157299 induces Smad4 ubiquitination in dLN cells of melanoma-bearing mice. Interaction between Smad4 and ubiquitin in dLN cells of vehicle- or LY-2157299-treated melanoma-bearing mice was determined by PLA. Images were acquired by confocal microscope, LSM700 (scale bars: 5 µm, 50 µm).

Supporting Information Figure 7

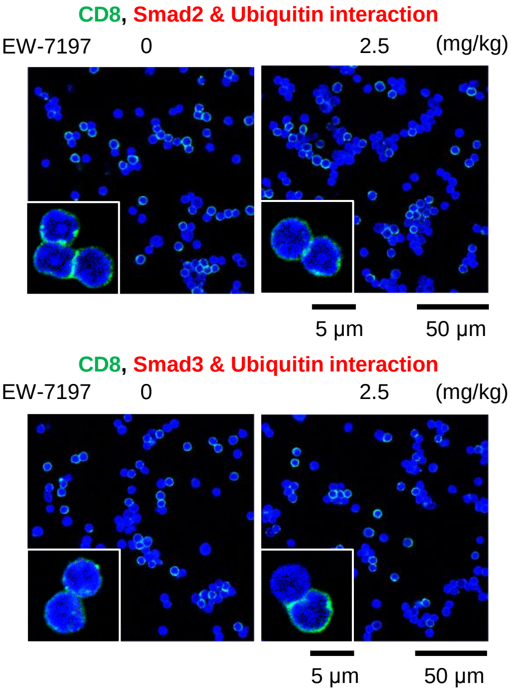

Supporting Information Figure 7. R-Smads are not ubiquitinated in dLN cells of EW-7197-treated melanoma-bearing mice. Interaction between Smad2 and ubiquitin or Smad3 and ubiquitin in dLN cells of vehicle- or EW-7197-treated melanoma-bearing mice was determined by PLA. Images were acquired by confocal microscope, LSM700 (scale bars: 5 µm, 50 µm).

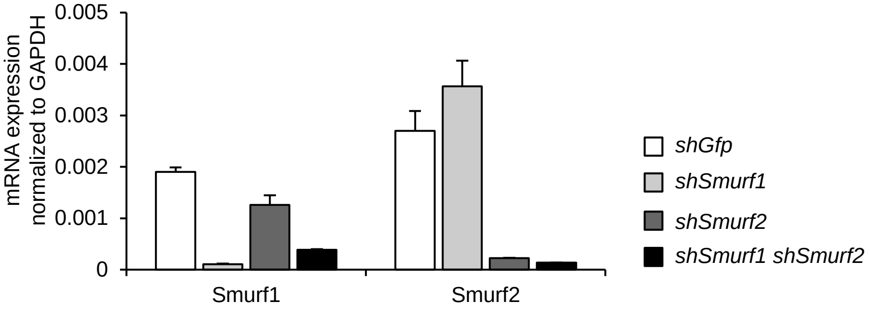

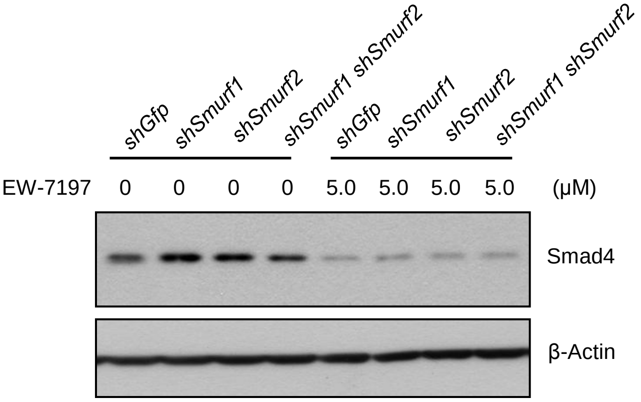

Supporting Information Figure 8. Smurf1 and Smurf2 are not involved in degradation of Smad4 by ALK5 inhibition. Knockdown of Smurf1 and/or Smurf2 by shRNA was confirmed by qPCR. Western blots show Smad4 and β-Actin in $CD8^+$ T cells stimulated with anti-CD3/28 antibodies with or without EW-7197 in vitro.

Supporting Information Figure 9

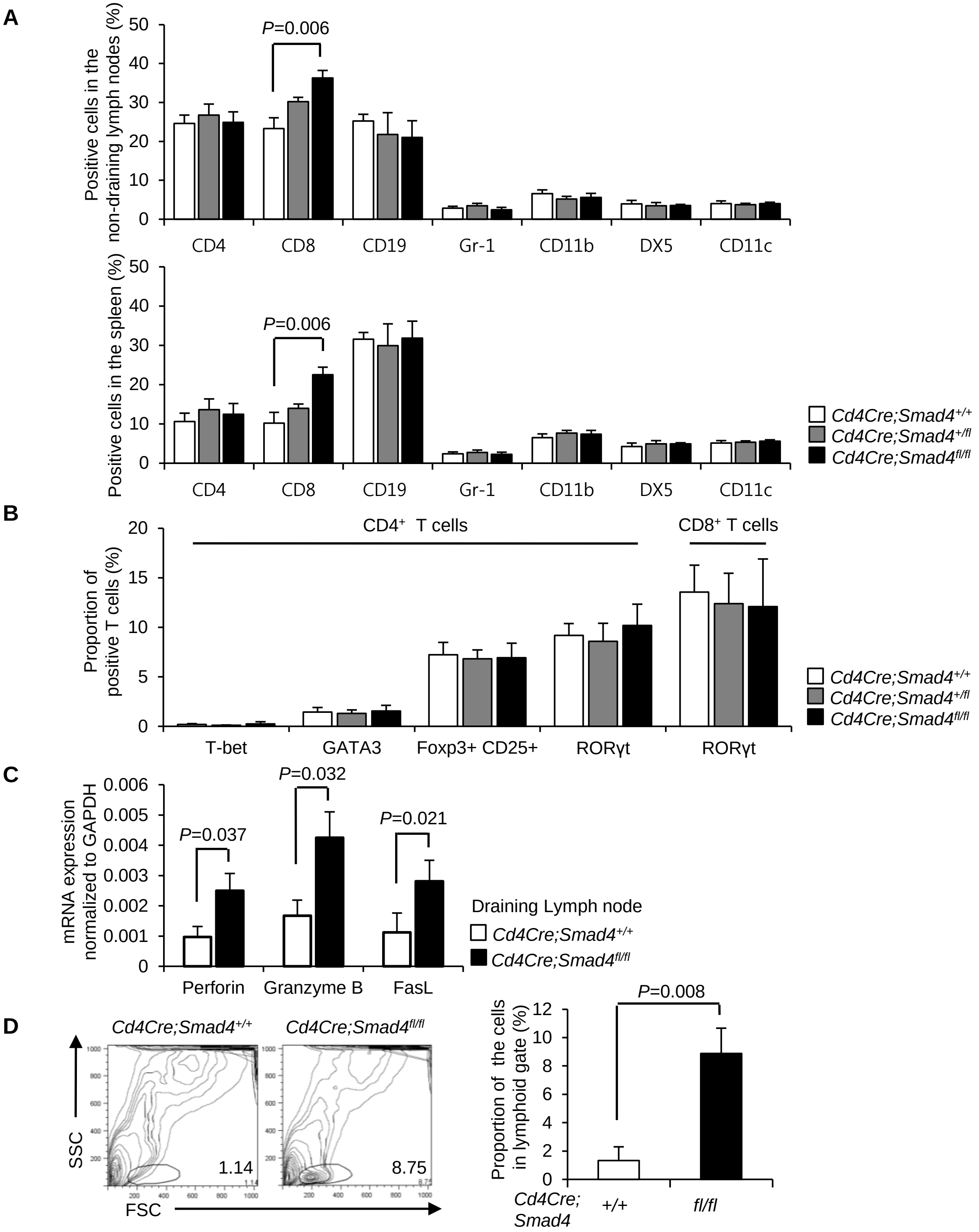

Supporting Information Figure 9. T cell-specific Smad4 deletion suppresses melanoma and LN metastases with enhanced CTL activity. Data are shown as mean + SEM (*n* = 25/group). *P* values were calculated by 2-tailed unpaired Student's *t* test. **A.** Percentages of immune cell subsets in the non-dLNs and spleens (*n* = 25/genotype). **B.** Percentages of effector T cell subsets in the dLNs. **C.** qPCR analyses for mRNA levels of the cytolytic molecules in the dLNs. **D.** Representative FSC/SSC contour plots of enriched tumor-infiltrated cells by density gradient centrifugation.

Supporting Information Figure 10

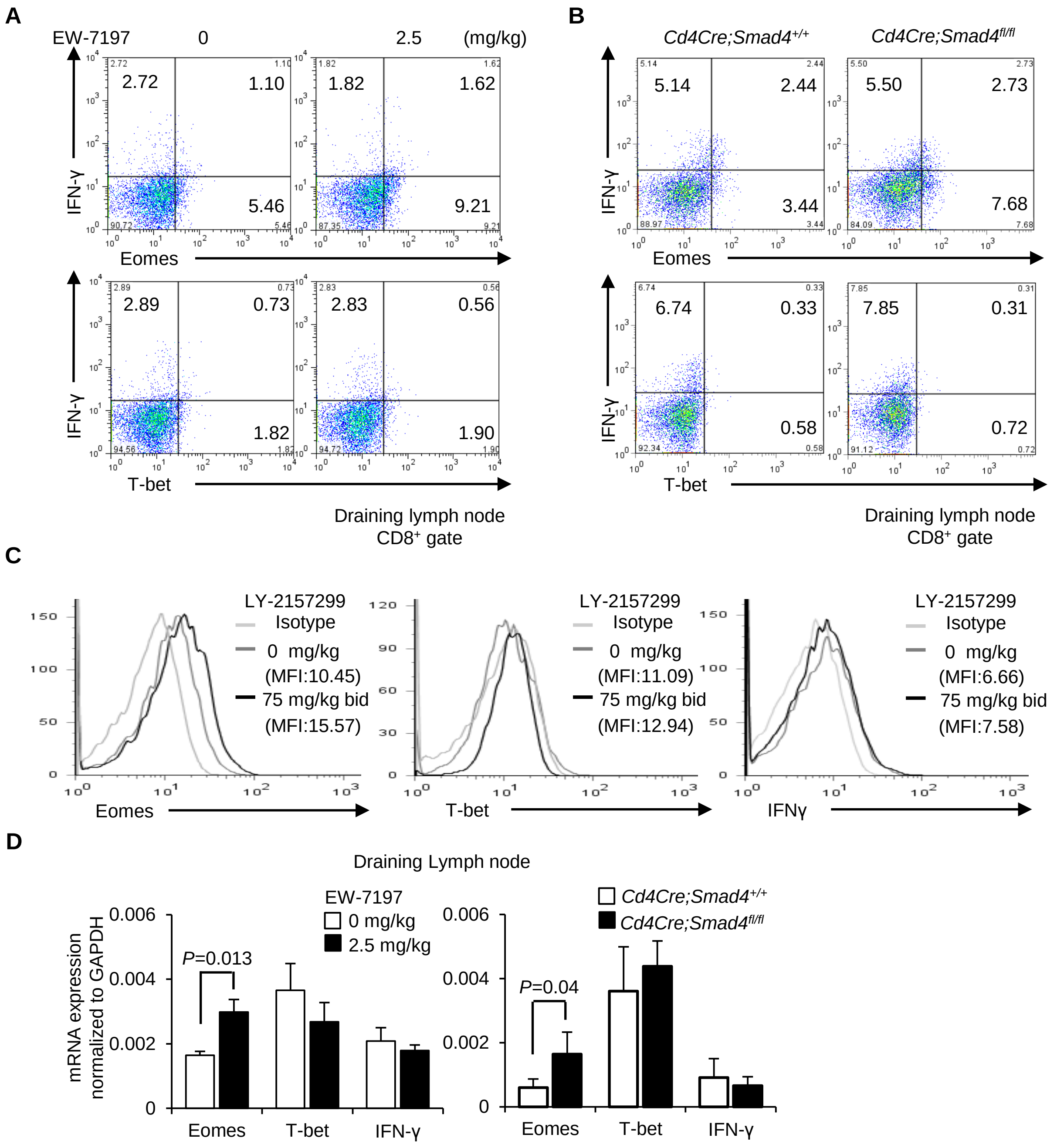

Supporting Information Figure 10. Upregulation of Eomes in CD8^{+} T cells by ALK5 inibition or T cell-specific Smad4 deletion. A. Representative dot plots show the expression of Eomes, T-bet, and IFN-γ in dLN CD8^{+} cells of vehicle- or EW-7197-treated melanoma-bearing mice. **B.** Representative dot plots show the expression of Eomes, T-bet, and IFN-γ in dLN CD8^{+} cells of *Cd4Cre;Smad4$^{+/+}$* or *Cd4Cre;Smad4$^{fl/fl}$* mice. **C.** Representative histograms show the expression of Eomes, T-bet, and IFN-γ in dLN CD8^{+} cells of vehicle- or LY-2157299-treated melanoma-bearing mice. **D.** qPCR analyses for mRNA levels in dLN cells of vehicle- or EW-7197-treated melanoma-bearing and *Cd4Cre;Smad4$^{+/+}$* or *Cd4Cre;Smad4$^{fl/fl}$* mice (n = 10/group, n = 10/genotype).

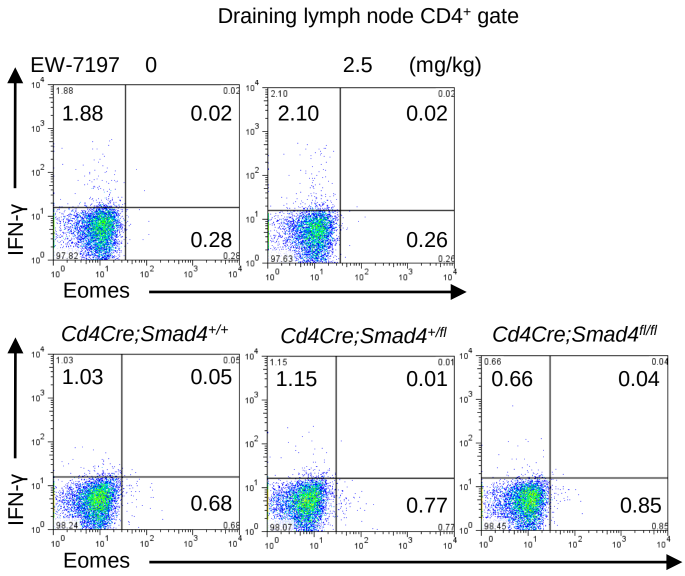

Supporting Information Figure 11. Eomes is not expressed in CD4+ cells in the dLNs of melanoma-bearing mice. Representative dot plots show the expression of IFN-γ and Eomes in CD4+ cells from vehicle-treated, EW-7197-treated, *Cd4Cre;Smad4+/+*, *Cd4Cre;Smad4+/fl*, and *Cd4Cre;Smad4fl/fl* mice.

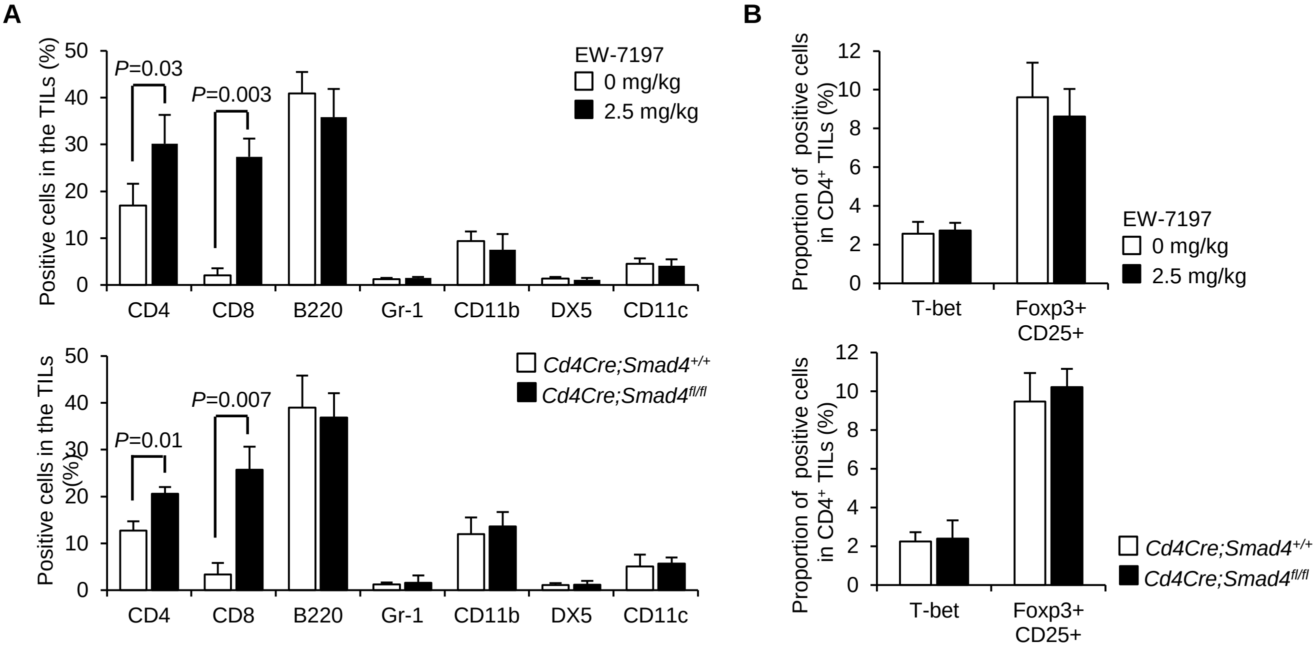

Supporting Information Figure 12. Characterization of TILs. TILs subsets in melanomas of control, EW-7197-treated or *Smad4*$^{-/-}$ mice were determined by flowcytometry. Graphs show the percentages of positive cells. **A.** Percentages of immune cell subsets in TIL gates were determined by flowcytometry. **B.** Percentages of T-bet^{+} or Foxp3^{+}CD25^{+} in CD4^{+} TIL gates were determined by flowcytometry.

Supporting Information Figure 13

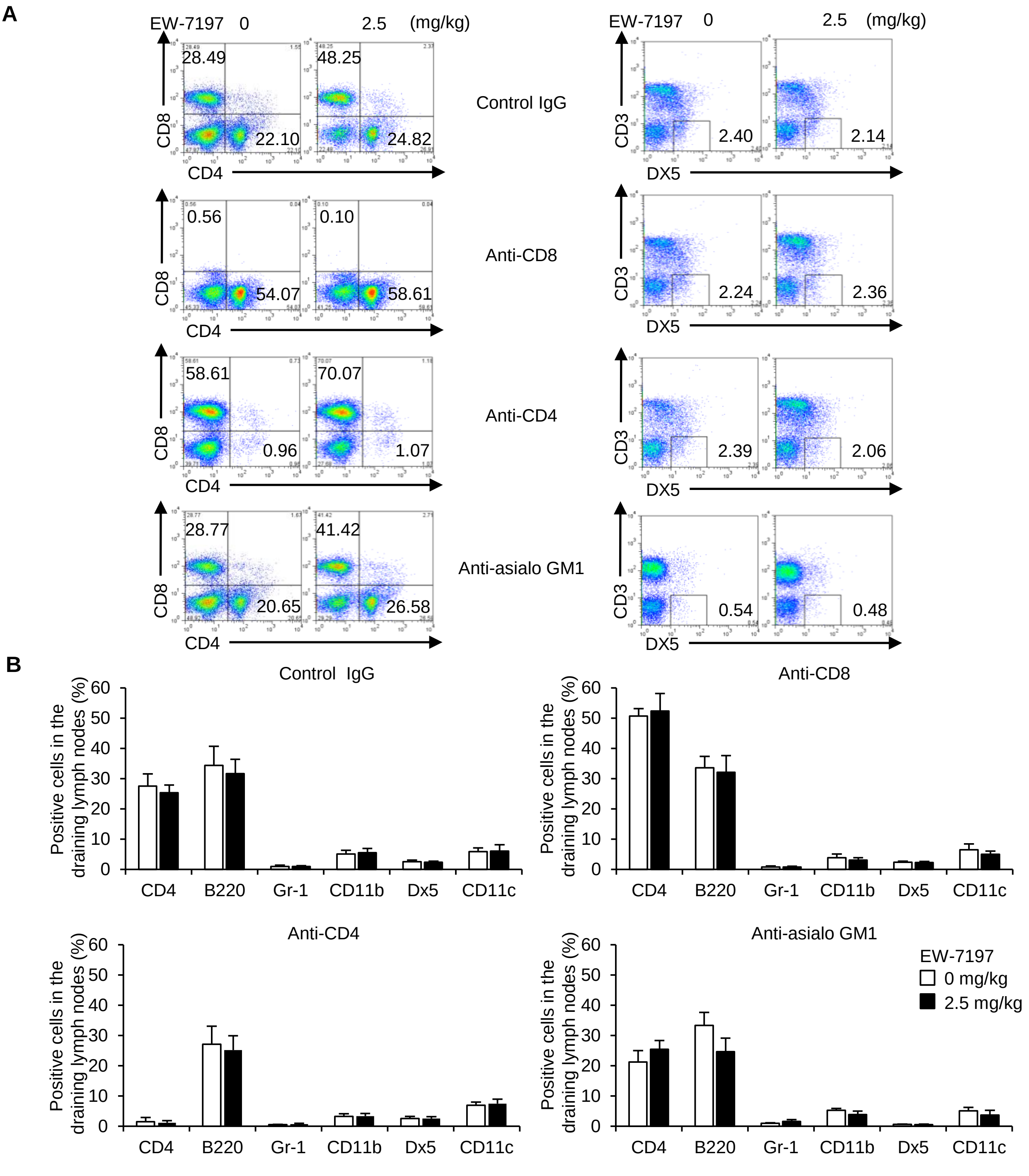

Supporting Information Figure 13. Deletion of CD8+ T cells, CD4+ T cells, NK cells in vivo. C57BL/6 mice were i.p. injected with control, anti-CD8, anti-CD4, or anti-asialo GM1 antibody at day -4, 0, 7, and 14 of melanoma inoculation (day 0), with vehicle or EW-7197 from 4 days after inoculation of GFP-expressing B16 cells (2×10^5) into the left lower abdomen (n = 5-8/group). Data are shown as mean + SEM. **A.** Representative dot plots are shown for each antibody treatment. **B.** Graphs show the percentages of immune cell subsets.

Supporting Information Figure 14

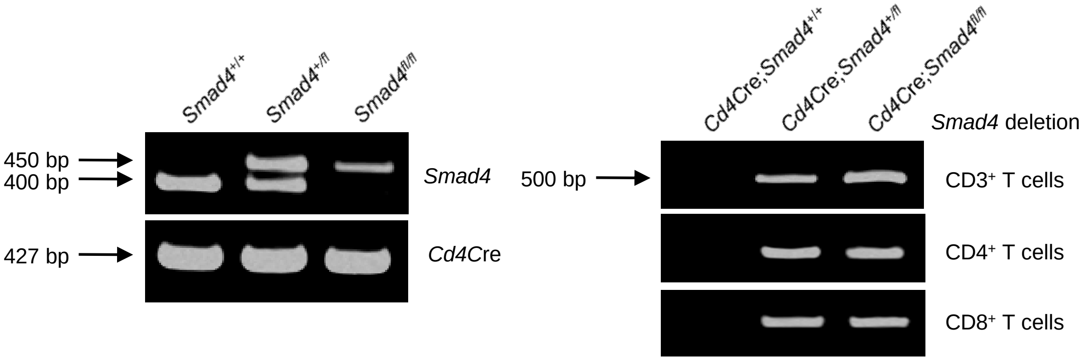

Supporting Information Figure 14. T cell-specific Smad4 deletion. Genomic DNA was obtained from $CD3^{+}$ T cells, $CD4^{+}$ T cells, and $CD8^{+}$ T cells sorted by MACS from the spleens and superficial LNs of *Cd4Cre;Smad4*$^{+/+}$, *Cd4Cre;Smad4*$^{+/fl}$, and *Cd4Cre;Smad4*$^{fl/fl}$ mice. Deletion of the *Smad4* gene was confirmed by PCR by the primers reported in the references (Kim et al, 2006; Lee et al, 2001; Yang et al, 1999).

Supporting Information Figure 15

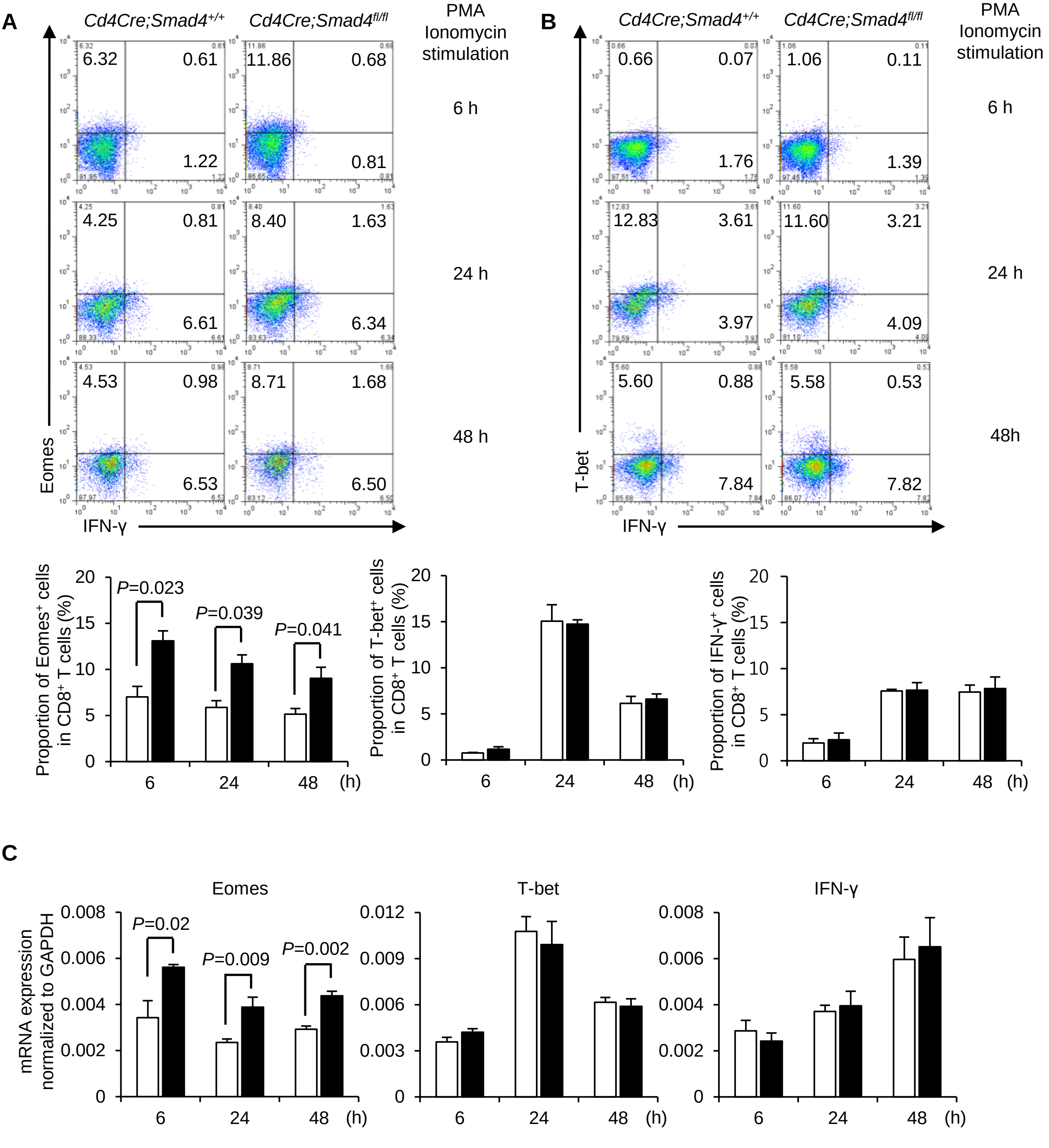

Supporting Information Figure 15. Effect of Smad4 on PMA/ionomycin-stimulated CD8$^+$ T cells. CD8$^+$ cells from the indicated mice were stimulated with PMA and ionomycin for 3 days. **A, B.** Representative dot plots show Eomes/IFN-γ, T-bet/IFN-γ in CD8$^+$ cells from *Cd4Cre;Smad4*$^{+/+}$/*Cd4Cre;Smad4*$^{fl/fl}$ mice (n = 2/genotype). **C.** qPCR analyses for Eomes, T-bet, and IFN-γ mRNA levels in CD8$^+$ cells from *Cd4Cre;Smad4*$^{+/+}$/*Cd4Cre;Smad4*$^{fl/fl}$ mice (n = 2/genotype).
