# Supplementary material for: Activin receptor-like kinase5 inhibition suppresses mouse melanoma by ubiquitin degradation of Smad4, thereby derepressing eomesodermin in cytotoxic T lymphocytes
Source: EMBO Mol Med. 2013 Oct 11;5(11):1720–39. doi: 10.1002/emmm.201302524 (PMC3840488; doi:10.1002/emmm.201302524)

Figure 2G

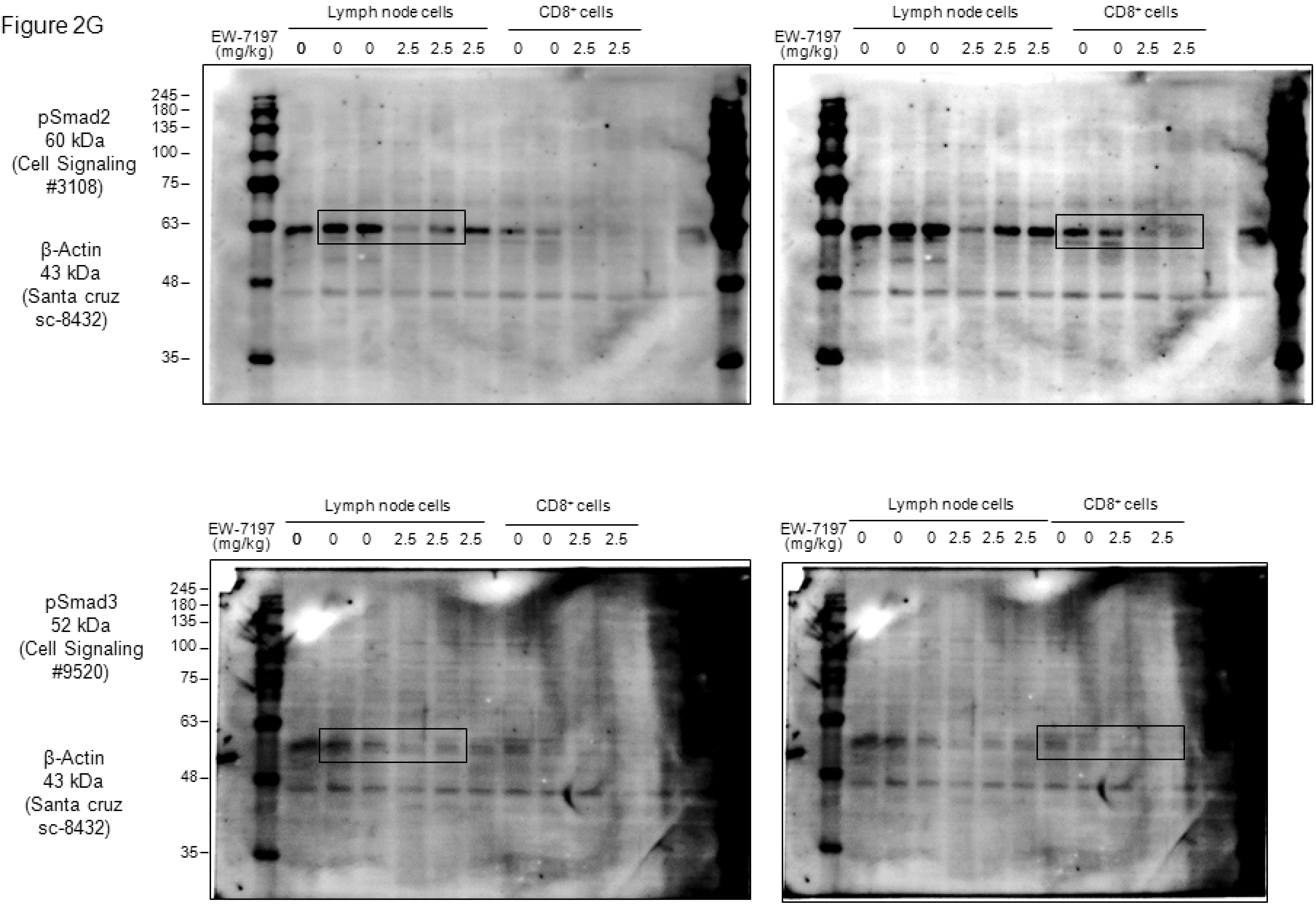
Lymph node cells
CD8+ cells
EW-7197 (mg/kg)
0 0 0 2.5 2.5 2.5 0 0 2.5 2.5
pSmad2
60 kDa
(Cell Signaling #3108)
β-Actin
43 kDa
(Santa cruz sc-8432)
245
180
135
100
75
63
48
35
Lymph node cells
CD8+ cells
EW-7197 (mg/kg)
0 0 0 2.5 2.5 2.5 0 0 2.5 2.5
Lymph node cells
CD8+ cells
EW-7197 (mg/kg)
0 0 0 2.5 2.5 2.5 0 0 2.5 2.5
pSmad3
52 kDa
(Cell Signaling #9520)
β-Actin
43 kDa
(Santa cruz sc-8432)
245
180
135
100
75
63
48
35
Lymph node cells
CD8+ cells
EW-7197 (mg/kg)
0 0 0 2.5 2.5 2.5 0 0 2.5 2.5

Figure 2G

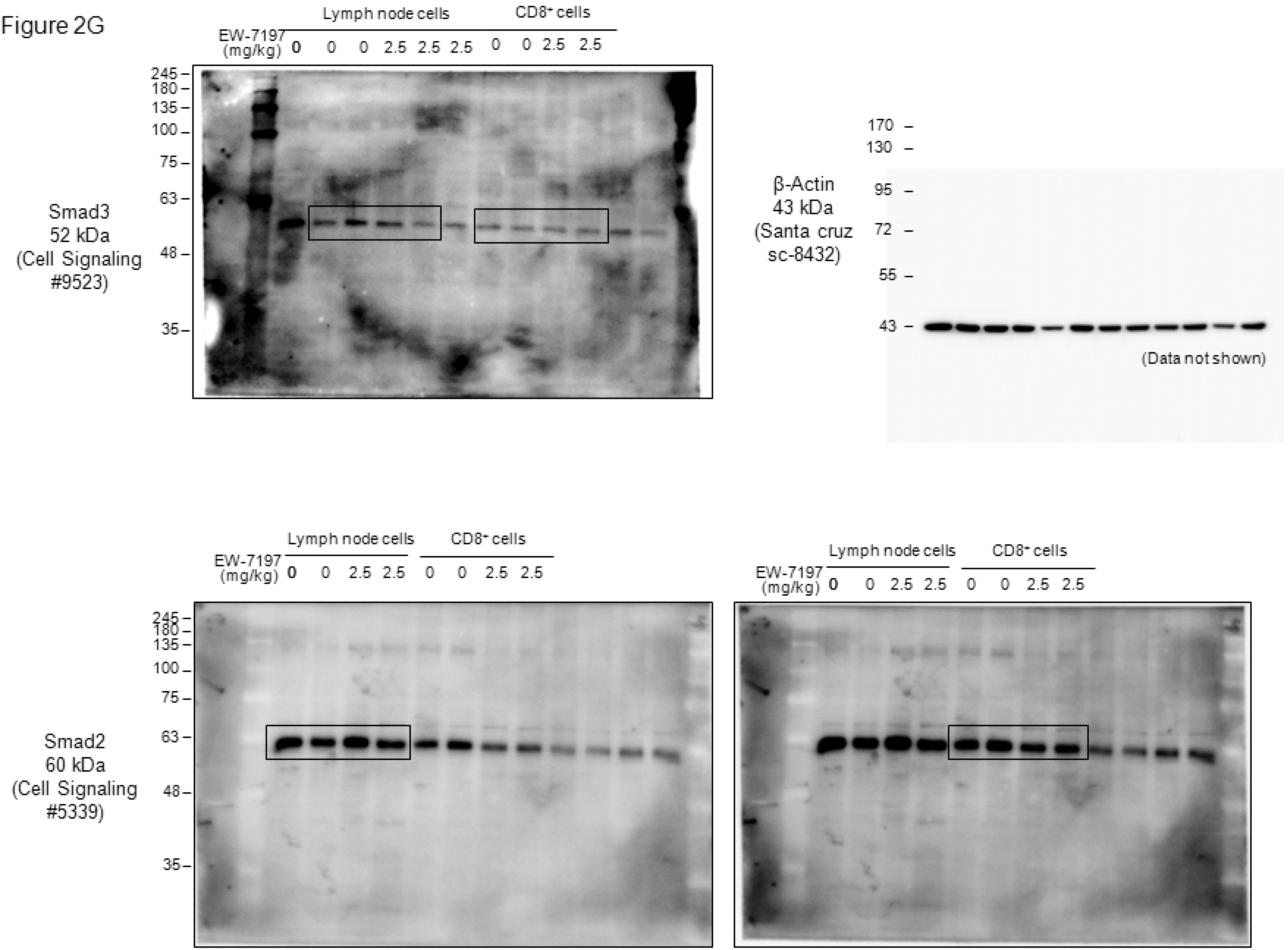

Lymph node cells
CD8+ cells
EW-7197 (mg/kg) 0 0 0 2.5 2.5 2.5 0 0 2.5 2.5
245 180 135 100 75 63 48 35
Smad3
52 kDa
(Cell Signaling #9523)
β-Actin
43 kDa
(Santa cruz sc-8432)
170 130 95 72 55 43
(Data not shown)
Lymph node cells
CD8+ cells
EW-7197 (mg/kg) 0 0 2.5 2.5 0 0 2.5 2.5
Smad2
60 kDa
(Cell Signaling #5339)

Figure 2G

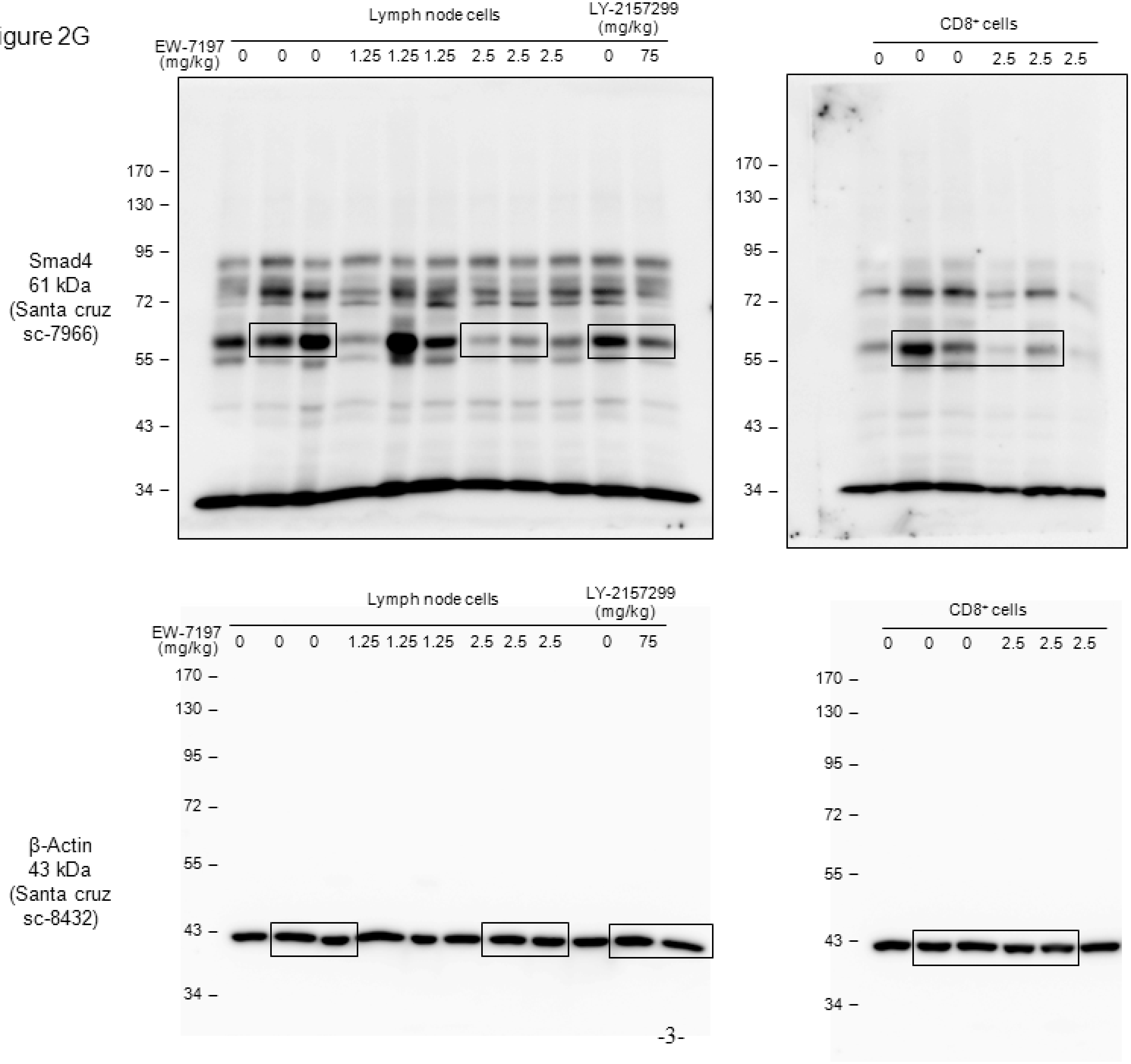
Lymph node cells
LY-2157299 (mg/kg)
EW-7197 (mg/kg)
0 0 0 1.25 1.25 1.25 2.5 2.5 2.5 0 75
CD8+ cells
0 0 0 2.5 2.5 2.5
170
130
95
72
55
43
34
Smad4
61 kDa
(Santa cruz sc-7966)
β-Actin
43 kDa
(Santa cruz sc-8432)

Figure 3B

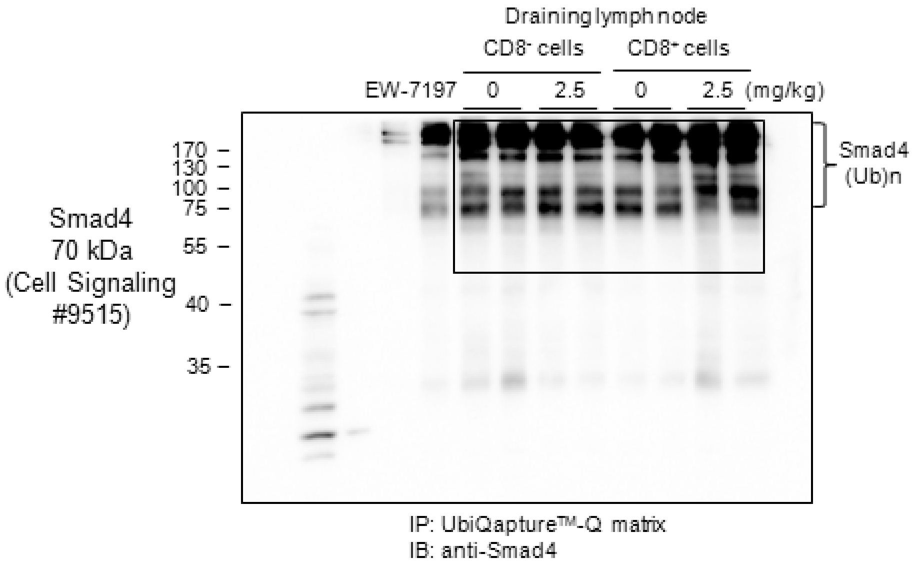
Draining lymph node
CD8- cells
CD8+ cells
EW-7197
0
2.5
0
2.5
(mg/kg)
Smad4
70 kDa
(Cell Signaling
#9515)
170
130
100
75
55
40
35
Smad4
(Ub)n
IP: UbiQapture™-Q matrix
IB: anti-Smad4

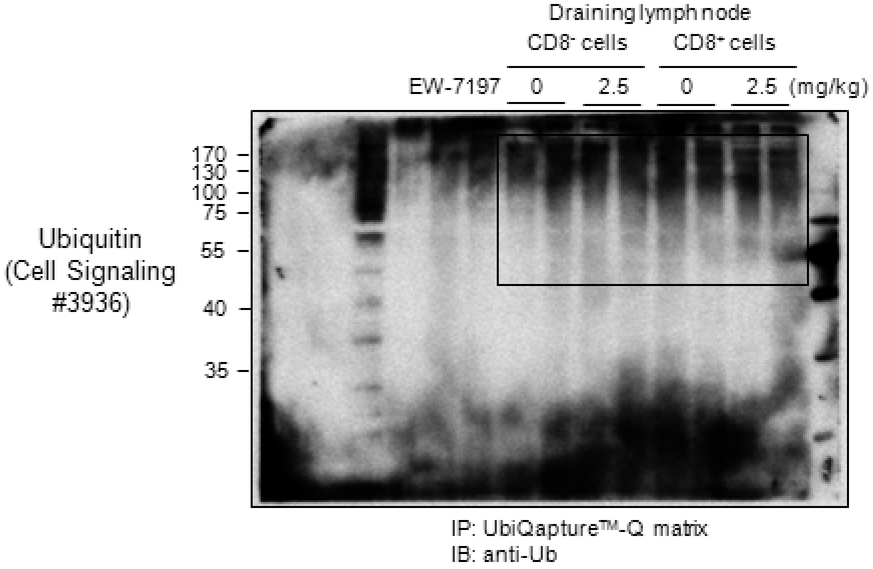
Draining lymph node
CD8- cells
CD8+ cells
EW-7197
0
2.5
0
2.5
(mg/kg)
Ubiquitin
(Cell Signaling
#3936)
170
130
100
75
55
40
35
IP: UbiQapture™-Q matrix
IB: anti-Ub

Figure 3C

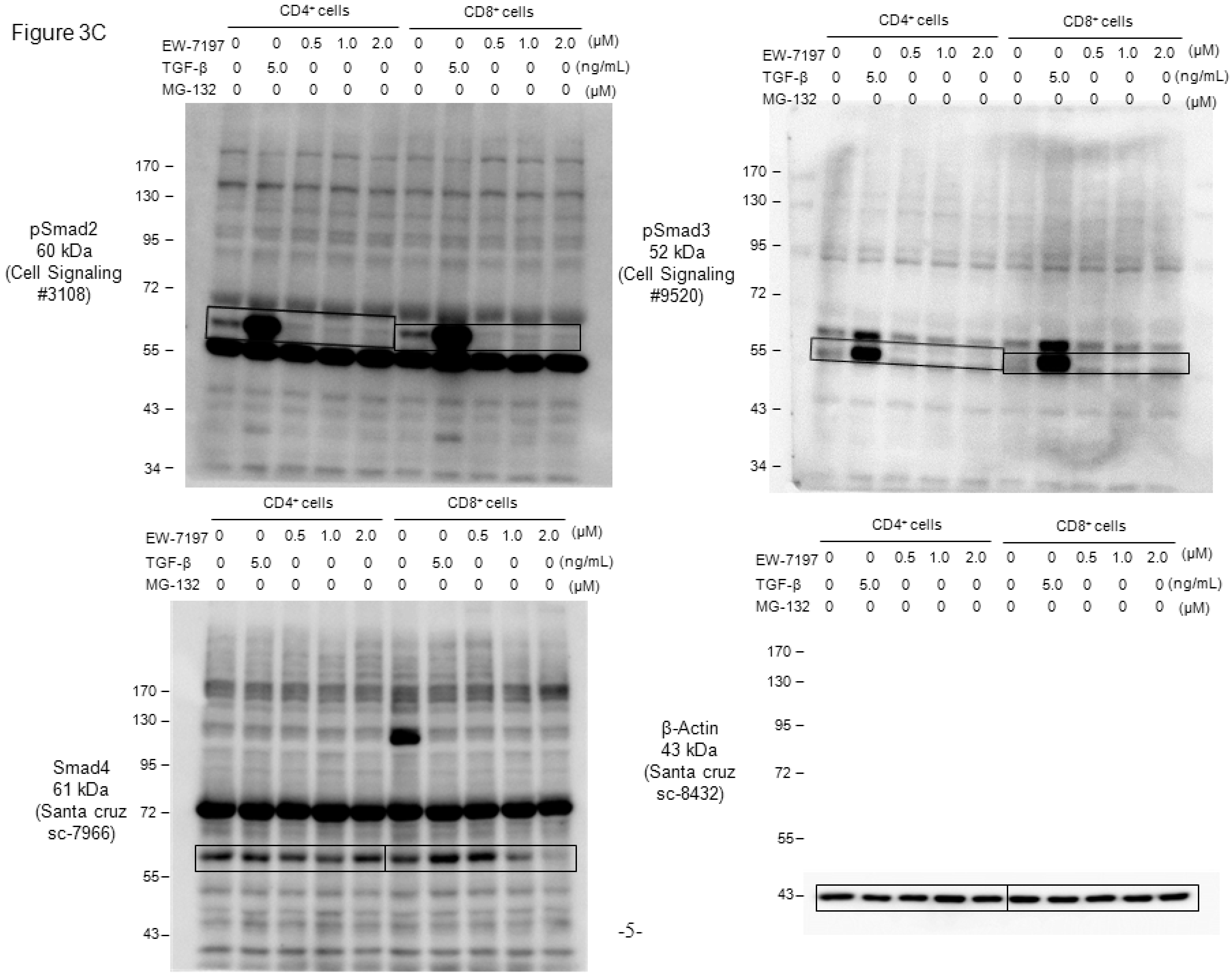
CD4⁺ cells
CD8⁺ cells
EW-7197 0 0 0.5 1.0 2.0 0 0 0.5 1.0 2.0 (μM)
TGF-β 0 5.0 0 0 0 0 5.0 0 0 0 (ng/mL)
MG-132 0 0 0 0 0 0 0 0 0 0 (μM)
170
130
95
72
55
43
34
pSmad2
60 kDa
(Cell Signaling
#3108)
pSmad3
52 kDa
(Cell Signaling
#9520)
Smad4
61 kDa
(Santa cruz
sc-7966)
β-Actin
43 kDa
(Santa cruz
sc-8432)

Figure 3C

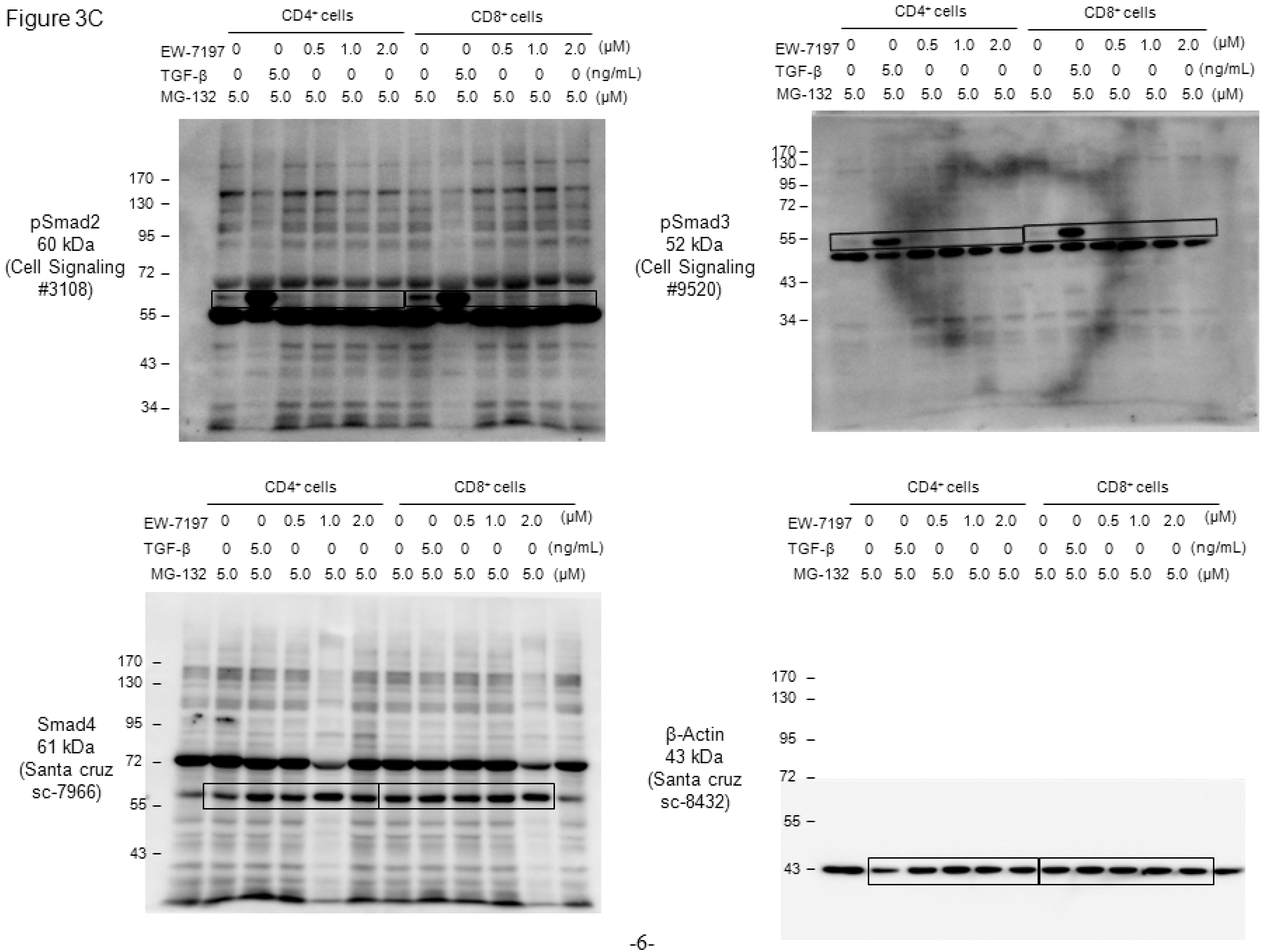

CD4⁺ cells
CD8⁺ cells
EW-7197 0 0 0.5 1.0 2.0 0 0 0.5 1.0 2.0 (μM)
TGF-β 0 5.0 0 0 0 0 5.0 0 0 0 (ng/mL)
MG-132 5.0 5.0 5.0 5.0 5.0 5.0 5.0 5.0 5.0 5.0 (μM)
pSmad2
60 kDa
(Cell Signaling
#3108)
170
130
95
72
55
43
34
CD4⁺ cells
CD8⁺ cells
EW-7197 0 0 0.5 1.0 2.0 0 0 0.5 1.0 2.0 (μM)
TGF-β 0 5.0 0 0 0 0 5.0 0 0 0 (ng/mL)
MG-132 5.0 5.0 5.0 5.0 5.0 5.0 5.0 5.0 5.0 5.0 (μM)
pSmad3
52 kDa
(Cell Signaling
#9520)
170
130
95
72
55
43
34
CD4⁺ cells
CD8⁺ cells
EW-7197 0 0 0.5 1.0 2.0 0 0 0.5 1.0 2.0 (μM)
TGF-β 0 5.0 0 0 0 0 5.0 0 0 0 (ng/mL)
MG-132 5.0 5.0 5.0 5.0 5.0 5.0 5.0 5.0 5.0 5.0 (μM)
Smad4
61 kDa
(Santa cruz
sc-7966)
170
130
95
72
55
43
CD4⁺ cells
CD8⁺ cells
EW-7197 0 0 0.5 1.0 2.0 0 0 0.5 1.0 2.0 (μM)
TGF-β 0 5.0 0 0 0 0 5.0 0 0 0 (ng/mL)
MG-132 5.0 5.0 5.0 5.0 5.0 5.0 5.0 5.0 5.0 5.0 (μM)
β-Actin
43 kDa
(Santa cruz
sc-8432)
170
130
95
72
55
43

Figure 3D

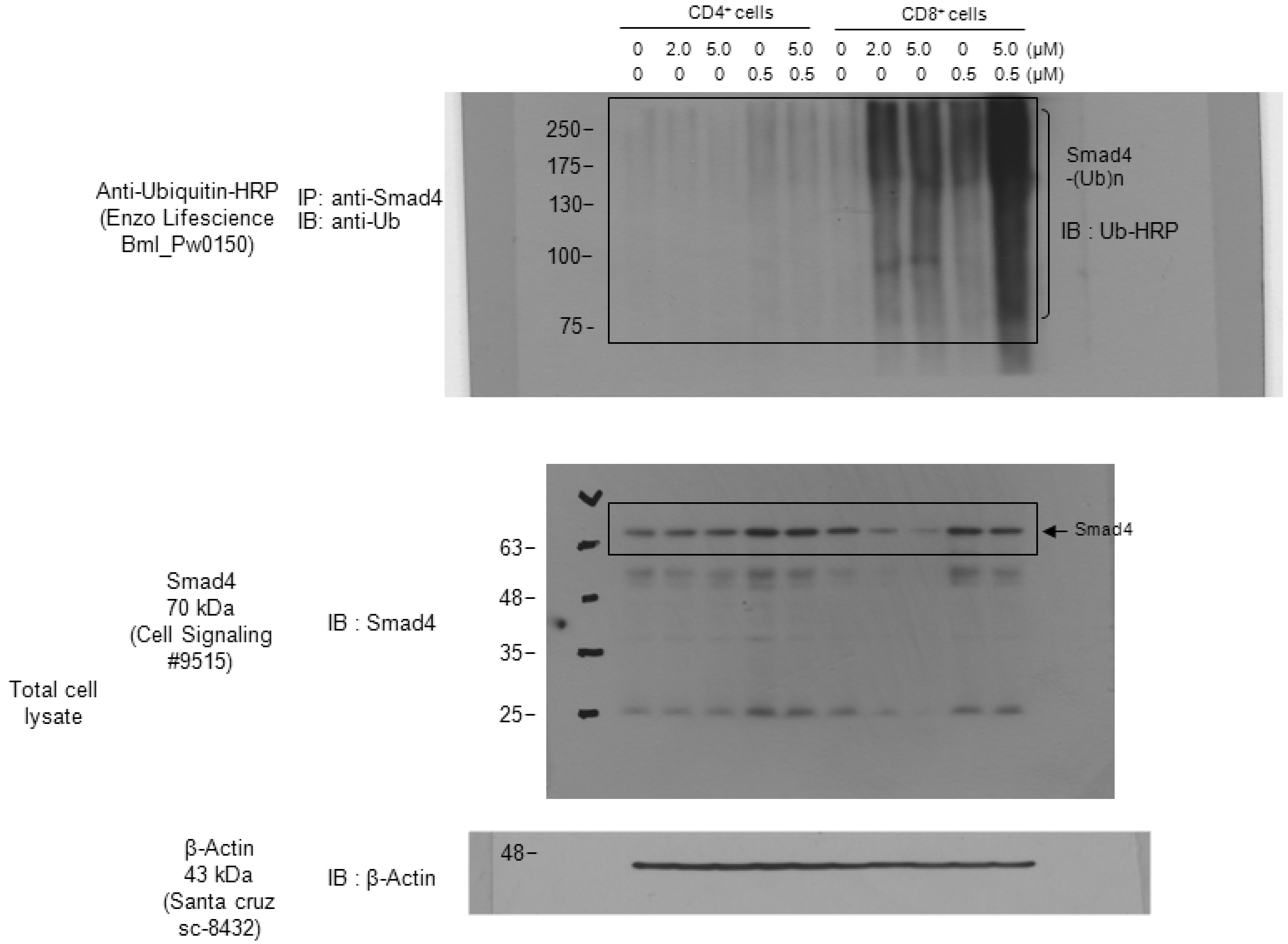
CD4+ cells
CD8+ cells
0 2.0 5.0 0 5.0 0 2.0 5.0 0 5.0 (μM)
0 0 0 0.5 0.5 0 0 0 0.5 0.5 (μM)
Anti-Ubiquitin-HRP
(Enzo Lifescience
Bml_Pw0150)
IP: anti-Smad4
IB: anti-Ub
250-
175-
130-
100-
75-
Smad4
-(Ub)n
IB : Ub-HRP
Smad4
70 kDa
(Cell Signaling
#9515)
IB : Smad4
63-
48-
35-
25-
Smad4
Total cell
lysate
β-Actin
43 kDa
(Santa cruz
sc-8432)
IB : β-Actin
48-

Combination with PLA probe anti-rabbit PLUS, PLA probe anti-mouse MINUS and rat anti-CD8 antibody
(Technical negative controls for Figure 2E, 3A and Supporting Information Figure 7)

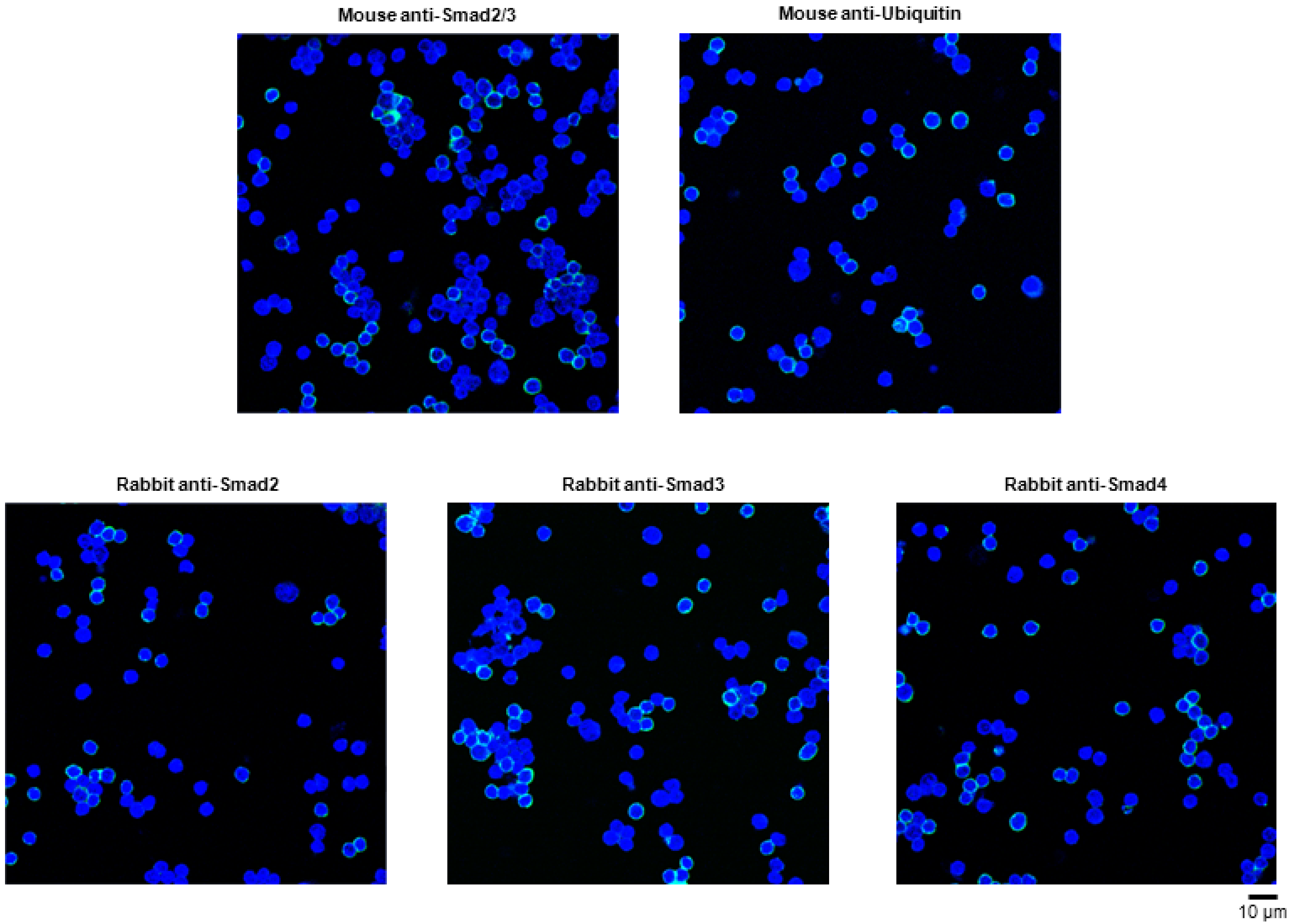

Supporting Information Figure 14

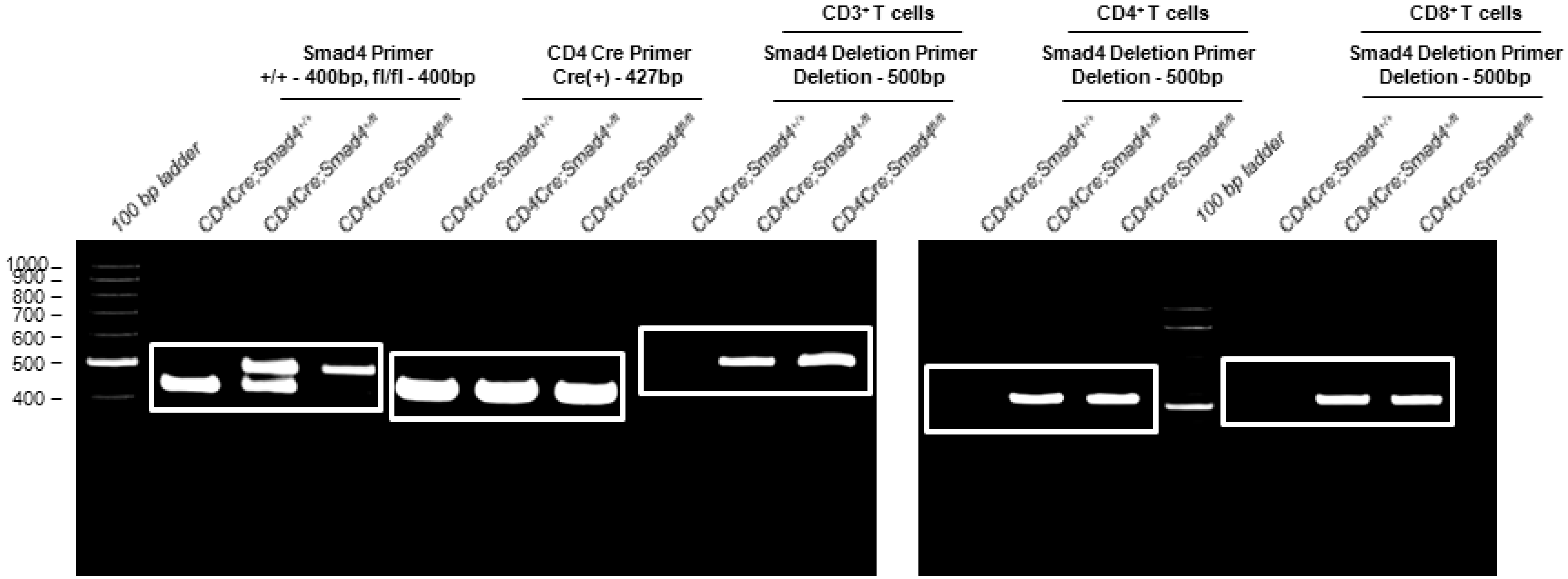

Supplement: Supplementary file 3 [file emmm0005-1720-SD3.pdf]
